# Gestational weight gain in low-income and middle-income countries: a modelling analysis using nationally representative data

**DOI:** 10.1136/bmjgh-2020-003423

**Published:** 2020-11-11

**Authors:** Dongqing Wang, Molin Wang, Anne Marie Darling, Nandita Perumal, Enju Liu, Goodarz Danaei, Wafaie W Fawzi

**Affiliations:** 1 Department of Global Health and Population, Harvard University TH Chan School of Public Health, Boston, Massachusetts, USA; 2 Department of Epidemiology, Department of Biostatistics, Harvard University TH Chan School of Public Health, Boston, Massachusetts, USA; 3 Institutional Centers for Clinical and Translational Research, Boston Children's Hospital, Boston, Massachusetts, USA; 4 Department of Global Health and Population, Department of Epidemiology, Harvard University TH Chan School of Public Health, Boston, Massachusetts, USA; 5 Department of Global Health and Population, Department of Epidemiology, Department of Nutrition, Harvard University TH Chan School of Public Health, Boston, Massachusetts, USA

**Keywords:** epidemiology, maternal health, public health

## Abstract

**Introduction:**

Gestational weight gain (GWG) has important implications for maternal and child health and is an ideal modifiable factor for preconceptional and antenatal care. However, the average levels of GWG across all low-income and middle-income countries of the world have not been characterised using nationally representative data.

**Methods:**

GWG estimates across time were computed using data from the Demographic and Health Surveys Program. A hierarchical model was developed to estimate the mean total GWG in the year 2015 for all countries to facilitate cross-country comparison. Year and country-level covariates were used as predictors, and variable selection was guided by the model fit. The final model included year (restricted cubic splines), geographical super-region (as defined by the Global Burden of Disease Study), mean adult female body mass index, gross domestic product per capita and total fertility rate. Uncertainty ranges (URs) were generated using non-parametric bootstrapping and a multiple imputation approach. Estimates were also computed for each super-region and region.

**Results:**

Latin America and Caribbean (11.80 kg (95% UR: 6.18, 17.41)) and Central Europe, Eastern Europe and Central Asia (11.19 kg (95% UR: 6.16, 16.21)) were the super-regions with the highest GWG estimates in 2015. Sub-Saharan Africa (6.64 kg (95% UR: 3.39, 9.88)) and North Africa and Middle East (6.80 kg (95% UR: 3.17, 10.43)) were the super-regions with the lowest estimates in 2015. With the exception of Latin America and Caribbean, all super-regions were below the minimum GWG recommendation for normal-weight women, with Sub-Saharan Africa and North Africa and Middle East estimated to meet less than 60% of the minimum recommendation.

**Conclusion:**

The levels of GWG are inadequate in most low-income and middle-income countries and regions. Longitudinal monitoring systems and population-based interventions are crucial to combat inadequate GWG in low-income and middle-income countries.

**Key questions**

**What is already known?**

- Gestational weight gain (GWG) is an important determinant of maternal and child health and an emerging modifiable factor for preconceptional and antenatal care.
- Previous studies suggest a high burden of inadequate GWG in certain low-income and middle-income countries and regions.
- National and regional levels of GWG across all low-income and middle-income regions have not been characterised.

**What are the new findings?**

- We present the first effort to estimate the GWG levels across all low-income and middle-income countries and regions of the world.
- We find that Latin America and Caribbean had the highest average GWG, whereas Sub-Saharan Africa and North Africa and Middle East had the lowest average GWG.
- We also report a major burden of inadequate GWG in most low-income and middle-income countries and regions.

**What do the new findings imply?**

- Longitudinal monitoring systems and population-based interventions are needed to combat inadequate GWG in low-income and middle-income countries.

## Introduction

Adequate gestational weight gain (GWG) is an important measure of maternal health during pregnancy, with critical implications for maternal and newborn outcomes. Inadequate GWG has been associated with adverse pregnancy outcomes, including low birth weight,^1–5^ preterm birth,^1 3 4 6^ small for gestational age^1 2 4 6 7^ and fetal and neonatal death.^8 9^ On the other hand, excessive weight gain during pregnancy has also been linked with large for gestational age,^1 4 6 7 10^ caesarean delivery^1 4 6 11^ and postpartum weight retention.^1 2 12 13^ Emerging evidence suggests that excessive GWG may also increase the risk of overweight and obesity of children in their future lives.^12 14–17^

The National Academy of Medicine, formerly the Institute of Medicine (IOM), recommends that women of normal weight (body mass index (BMI) of 18.5–24.9 kg/m^2^) before pregnancy gain 11.5–16 kg of weight during pregnancy and that women who are underweight (BMI less than 18.5 kg/m^2^) before pregnancy gain 12.5–18 kg during pregnancy (with corresponding recommendations for overweight and obese women as well).^1^ The IOM recommendations are intended for use in antenatal care for women in high-income settings and can serve as benchmarks to understand the status of GWG in low-income and middle-income countries (LMICs) and to compare across countries.^18^ Previous work has examined the patterns of GWG using cohorts in high-income settings in Europe, North America and Oceania.^19^ The levels and adequacies of GWG have been assessed in only a few LMICs, and previous work has suggested a high burden of inadequate GWG in such settings.^18 20–23^ In the mean time, with the nutrition transition to unhealthy dietary patterns, the increasing prevalence of sedentary lifestyle and the rising trend of maternal overweight and obesity in LMICs,^24 25^ the burden of excessive GWG is also likely to increase.

GWG is an important modifiable factor for antenatal care^26^ and is increasingly considered for preconceptional interventions for improving pregnancy outcomes.^27^ Interventions promoting adequate GWG have the potential to improve maternal, fetal and child outcomes across the world and particularly in low-income or middle-income countries. There is a gap in our understanding of GWG levels and the burden of inadequate and excessive GWG in resource-limited settings. Filling this major gap is important to inform the design of potential interventions during pregnancy to promote optimal weight gain. Therefore, we aimed to characterise the average levels of GWG across all low-income and middle-income countries and regions of the world.

## Methods

### Data source

We used nationally representative data from the Demographic and Health Surveys (DHS) Program, which has been conducted in over 90 LMICs.^28 29^ The DHS collects key information on population, health and nutrition. It includes one measure of weight among women of reproductive age. For this analysis, we focused on women who were pregnant at the time of the survey (age range: 15–49 years). Gestational month was self-reported as time since the last menstrual period (in complete days, weeks or months), and supplemented by self-reported duration of pregnancy (in complete months) whenever the time since the last menstrual period was not available. Longitudinal weight data or data on prepregnancy weight were not available in the DHS Program.

### Estimating GWG from data source

To take advantage of the repeatedly collected cross-sectional data in the DHS, we used the methodology developed by Coffey,^18^ who previously calculated the average total GWG in India and Sub-Saharan Africa around the year 2005 using the DHS data. The cross-sectional gestational weight measures were regressed on the gestational months of each measurement using an ordinary least squares regression model, as shown below.

(1) $$GestationalWeight_{i}=\alpha +\beta \times GestationalMonth_{i}+[ControlVariables{]}_{i}$$

We restricted the model to weight measures collected during the second and third trimesters (ie, gestational month ≥3) while accounting for the complex sampling designs appropriate for each data set (cluster, stratification and sampling weights). The control variables included maternal age, total years of education, total number of children ever born, rural/urban residence and household wealth index in quintiles to increase the precision of the estimates and correct for possible selection into gestational age reporting.^18^ The addition of maternal height as a control variable did not considerably change the final estimates. We used the linear model because the trajectories of GWG within the second and third trimesters are not far from linearity,^18 30–32^ and the addition of non-linear terms to the model did not contribute considerably to model fit. As a result, β represents the average monthly weight gain in the second and third trimesters for each data set. Then, we computed the mean total GWG for the entire pregnancy as:

(2) (1+P)×6.5×β

where *P* represents the assumed weight gain during the first trimester relative to the second and third trimesters (as a percentage) and was extracted from the GWG charts of the LifeCycle Consortium.^19^ The constant 6.5 represents the duration of the second and third trimesters in months for a full-term pregnancy (40^0/7^ weeks−14^0/7^ weeks=26 weeks=6.5 months). We used this approach to compute the mean total GWG (in kg) for each available country in each available year. To reflect the uncertainty due to sampling variability, we computed 95% CI as (1+P)×6.5×(β±1.96×SE_β_), where SE_β_ represents the SE estimate of β.^18^ The DHS data sets included and the estimates from each data set are available in online supplemental appendix 1.

10.1136/bmjgh-2020-003423.supp1 Supplementary data

### Analytical framework

Drawing on the methodology used in previous studies that estimated the regional and national levels of maternal outcomes,^33–36^ we used a hierarchical mixed-effects modelling approach to estimate the mean total GWG for the year 2015 for all countries to facilitate cross-national comparisons. We constructed a two-level hierarchical model using the estimated total GWG (point estimates from the DHS data) as the dependent variable, and year and country-level covariates as the independent variables. Specifically, we included fixed intercept, fixed effect for year, fixed effect for geographical region, fixed effects for potential country-level predictors, random (country-specific) intercepts and random (country-specific) effects for year. The dependent variable, mean total GWG for a full-term pregnancy, had a fairly normal distribution that did not improve after log-transformation, thus was not transformed. We compared models with only a linear term for year and with additional restricted cubic splines terms for year using different numbers (3, 4 or 5) and locations of knots. We determined the functional form for year by choosing the model with the smallest Bayesian information criterion (BIC). We defined the geographical regions as the super-regions used in the Global Burden of Disease (GBD) Study.^37^ The GBD super-regions included (1) Sub-Saharan Africa; (2) Latin America and Caribbean; (3) Southeast Asia, East Asia and Oceania; (4) South Asia; (5) North Africa and Middle East; and (6) Central Europe, Eastern Europe and Central Asia.

### Selection of country-level covariates

The potential country-level covariates included neonatal mortality rate, low birthweight rate, proportion of women receiving four or more antenatal care visits, caesarean section rate, mean adult female BMI, gross domestic product (GDP) per capita, Gini index, Human Development Index (HDI), total fertility rate, adolescent fertility rate, percentage of adolescents aged 15–19 years out of all women of reproductive age, and adult female literacy rate. These covariates were predictors that may be associated with mean GWG at the population level. It is of note that the purpose of the hierarchical models was to build prediction instead of conduct causal estimation. Therefore, the country-level covariates were not necessarily causal determinants of GWG at the individual level, and some covariates may not temporarily precede GWG.

We obtained data of the country-level predictors from publicly available databases (online supplemental appendix 2), which also included estimates from the same DHS surveys that gave rise to the GWG estimates. We excluded candidate covariates with a high degree (>30%) of missing data during the years when gestational weight data were available in the DHS. The covariates excluded due to high levels of missingness were low birthweight rate, proportion of women receiving four or more antenatal care visits, caesarean section rate, Gini index, and adult female literacy rate. The remaining covariates had minimal missingness, and we imputed missing values using linear interpolation between existing data points for the country whenever there is a missing value.^34 36^ For missing years prior to the first data point or after the last data point, we used the first or last data point as long as it was within 5 years of the missing year. Missing years beyond 5 years of observed data were set to missing; this resulted in one country (Burkina Faso) missing the information at 1 year (1993) for HDI.

10.1136/bmjgh-2020-003423.supp2 Supplementary data

We examined the correlations among the country-level covariates using the variance inflation factor (VIF) and removed HDI, which had a VIF greater than 10. We then used the BIC to further guide variable selection by maximising the predictive power and avoiding overfitting. We removed one predictor at a time from the full model, starting with the predictor with the largest BIC from univariate analysis. If removing a predictor resulted in a lower BIC (ie, improved model fit), we dropped that predictor from the model. If the BIC became higher after removing a predictor (ie, reduced model fit), we retained the predictor in the model.^35^ We cycled through all the predictors once to arrive at the final model. The country-level covariates selected for the final model were mean adult female BMI, GDP per capita (natural log-transformed) and total fertility rate. Replacing the single covariate of mean adult female BMI with the percentages of women in each BMI category (underweight, normal weight, overweight and obese) as three separate covariates (with one category excluded as the reference) did not improve model fit.

$$MeantotalGWG_{ij}=\beta_{0}+\beta_{1}\times g(year{)}_{ij}+h(super-region{)}_{i}+$$

$$\beta_{2}\times (meanadultfemaleBMI{)}_{ij}+\beta_{3}\times ln(GDPpercapita{)}_{ij}+$$

(3)$\beta_{4}\times (totalfertilityrate{)}_{ij}+b_{0i}+b_{1i}\times g(year{)}_{ij}$

The final hierarchical model is summarised in equation (3), where *i* and *j* refer to country and time point, respectively; *g(year)* represents a restricted cubic spline function for year with three knots (quartiles) placed at years 1998, 2005.5 and 2012; and *h(super-region)* represents a function associated with dummy variables representing GBD super-regions.

### Statistical analysis

To obtain national estimates, we used the parameter estimates (fixed effects and random effects) from the final hierarchical model to calculate the mean total GWG for a full-term pregnancy for each country in 2015. The year 2015 was chosen to minimise extrapolation, as 2015 was the latest year available for many countries in the DHS surveys. To summarise the estimates at the super-regional and regional levels, we weighted the national estimates from the hierarchical model by the total number of births in 2015 of each country from the World Population Prospects 2019.^38^

We used non-parametric bootstrapping to generate estimates of uncertainty. We drew 1000 bootstrap samples from the estimation data set, repeated the estimation process using each bootstrap sample and used the parameter estimates obtained from each sample to generate a new set of estimates. For countries not included in the bootstrap sample and thus did not have country-specific random effect estimates, we used the variance of random effects to randomly draw the country-specific effects.^33–35^ For countries that did not have any data available, we assumed the country-specific random effect to be zero and used the fixed effect estimates only.^33–35^ We considered all countries classified as low-income, lower-middle-income or upper-middle-income by the World Bank for the 2020 fiscal year, which was the most updated classification at the time of analyses.

To further account for the uncertainty from using the cross-sectional DHS data in the estimation of GWG, we used a multiple imputation approach to derive the final variance estimators.^39^ Specifically, for each country in each year available in the DHS, we randomly generated a new estimate from a normal distribution based on the original point estimate and the SE estimate. We generated 10 pseudo data sets and used bootstrapping, as described above, on each pseudo data set. Thus, we generated 10 pseudo point estimates of mean total GWG and 10 bootstrapped SE estimates for each country. We then used the 10 sets of point estimates and SEs to derive the final SE,^39^ which was then used to compute the 95% uncertainty range (UR). We compared all estimates against the IOM recommendations as benchmarks; the specific GWG values recommended in the IOM guideline were not used in the analyses and thus did not affect the estimation in any way. We downloaded the DHS data from the DHS Program website (www.dhsprogram.com). All analyses were conducted using SAS V.9.4. Statistical codes are available on request.

### Patient and public involvement

It was not possible to involve patients or the public in this research.

## Results

From among all 137 LMICs, the DHS Program had gestational weight data from 67 countries across 206 country-years, with 114 country-years coming from Sub-Saharan Africa (table 1). Gestational weight data in the DHS were evenly distributed across the second and third trimesters; 58% and 42% of gestational weight measures were from second and third trimesters, respectively (excluding first-trimester weights). Seventy countries did not participate in the DHS Program or did not have any gestational weight data in the DHS. There was fair to good proportional representation for Sub-Saharan Africa (36 out of 46 countries), South Asia (4 out of 5), and Latin American and Caribbean (10 out of 25). Relatively fewer countries were covered in Southeast Asia, East Asia and Oceania (4 out of 25), North Africa and Middle East (5 out of 15), and Central Europe, Eastern Europe and Central Asia (8 out of 21). Notably, no DHS data were available for any LMICs in East Asia (ie, China and North Korea) or Oceania.

**Table 1:** Number of countries and data points included in the analysis by Global Burden of Disease super-region and region

	Countries (n)	Countries with at least one data point in the DHS (n)	Total data points in the DHS (n)	Countries without data in the DHS* (n)
Sub‐Saharan Africa	46	36	114	10
Central Sub-Saharan Africa	6	4	7	2
Eastern Sub-Saharan Africa	15	11	43	4
Southern Sub-Saharan Africa	6	5	13	1
Western Sub-Saharan Africa	19	16	51	3
Latin America and Caribbean	25	10	32	15
Andean Latin America	3	2	12	1
Caribbean	11	3	9	8
Central Latin America	8	4	10	4
Tropical Latin America	3	1	1	2
Southeast Asia, East Asia and Oceania	25	4	9	21
East Asia	2	0	0	2
Southeast Asia	12	4	9	8
Oceania	11	0	0	11
Central Europe, Eastern Europe and Central Asia	21	8	14	13
Central Asia	9	6	11	3
Central Europe	8	1	2	7
Eastern Europe	4	1	1	3
South Asia	5	4	16	1
North Africa and Middle East	15	5	21	10
Total	137	67	206	70

*Estimates for South Sudan and Kosovo could not be computed due to lack of sufficient covariate data in 2015.

DHS, Demographic and Health Surveys.

**Figure 1:**
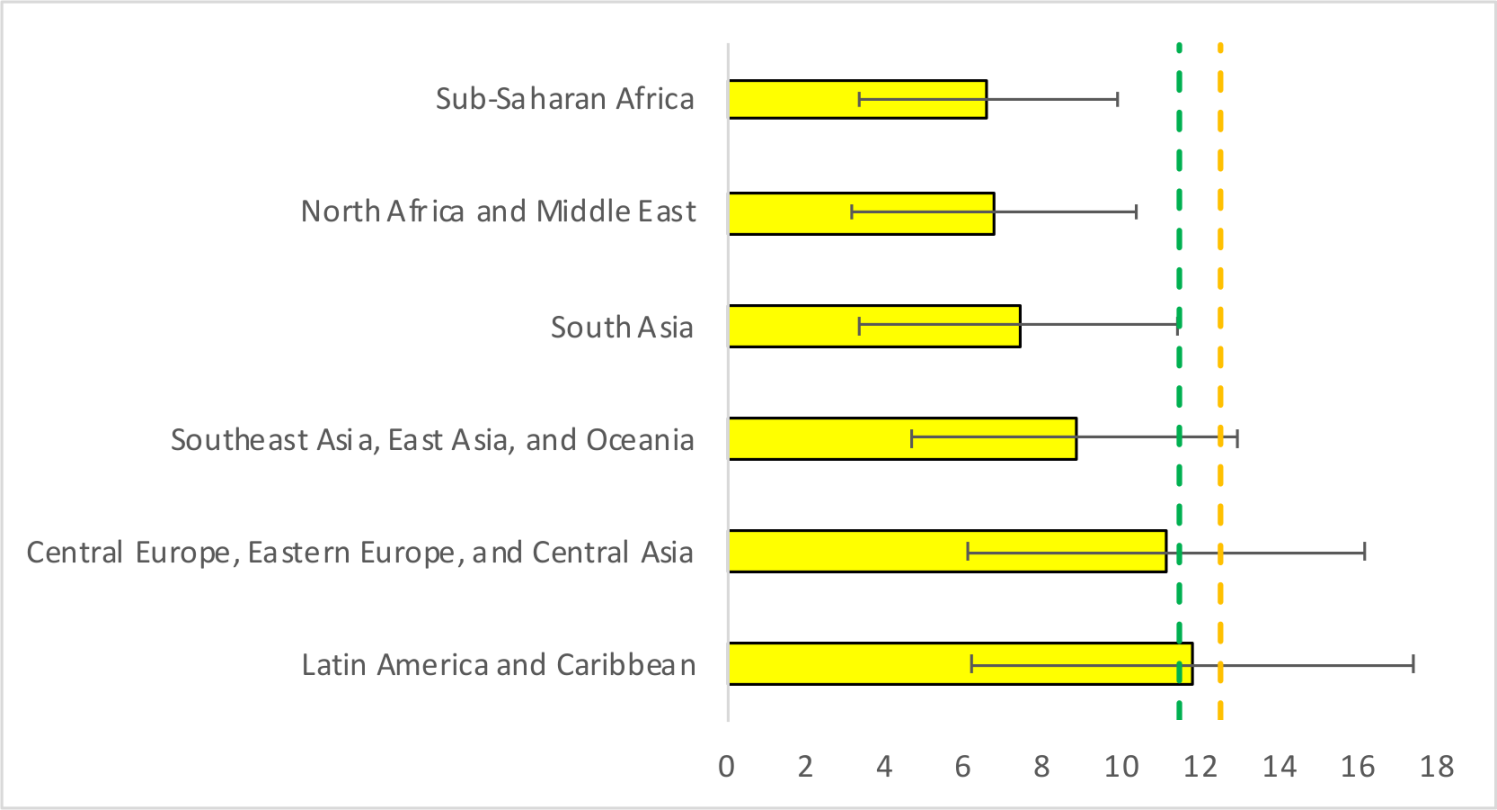
Estimated gestational weight gain in 2015 by Global Burden of Disease super-region. The green and orange dashed lines represent the minimum Institute of Medicine recommendations of total gestational weight gain for normal-weight (11.5 kg) and underweight (12.5 kg) women, respectively.

At the super-regional level, pregnant women in Latin America and Caribbean, and Central Europe, Eastern Europe and Central Asia had the highest estimated GWG. However, Latin America and Caribbean (11.8 kg (95% UR: 6.2, 17.4)) barely met the minimum recommendation of 11.5 kg set by the IOM for normal-weight women, while Central Europe, Eastern Europe and Central Asia (11.2 kg (95% UR: 6.2, 16.2)) met 97% of the recommendation. Women in Sub-Saharan Africa (6.6 kg (95% UR: 3.4, 9.9)) and North Africa and Middle East (6.8 kg (95% UR: 3.2, 10.4)) had the lowest estimated GWG; these super-regions met less than 60% of the minimum GWG recommendation for normal-weight women. Women in Southeast Asia, East Asia and Oceania (8.8 kg (95% UR: 4.7, 12.9)) and in South Asia (7.4 kg (95% UR: 3.4, 11.4)) met 77% and 64% of the minimum recommendation for normal-weight women, respectively (table 2 and figure 1).

**Table 2:** Regional estimates of mean total gestational weight gain in 2015 by Global Burden of Disease super-region and region*

	Estimated mean GWG (kg)
Sub‐Saharan Africa	6.6 (3.4, 9.9)
Central Sub-Saharan Africa	5.3 (1.3, 9.4)
Eastern Sub-Saharan Africa	6.2 (2.9, 9.5)
Southern Sub-Saharan Africa	8.6 (1.8, 15.4)
Western Sub-Saharan Africa	7.2 (3.2, 11.2)
Latin America and Caribbean	11.8 (6.2, 17.4)
Andean Latin America	10.2 (5.6, 14.9)
Caribbean	11.3 (5.5, 17.2)
Central Latin America	11.1 (7.0, 15.1)
Tropical Latin America	13.2 (2.3, 24.1)
Southeast Asia, East Asia and Oceania	8.8 (4.7, 12.9)
East Asia	9.1 (4.8, 13.5)
Southeast Asia	8.4 (4.6, 12.3)
Oceania	7.7 (3.0, 12.5)
Central Europe, Eastern Europe and Central Asia	11.2 (6.2, 16.2)
Central Asia	9.9 (3.9, 15.9)
Central Europe	12.0 (6.8, 17.2)
Eastern Europe	12.1 (6.9, 17.4)
South Asia	7.4 (3.4, 11.4)
North Africa and Middle East	6.8 (3.2, 10.4)

*Values are the estimated mean gestational weight gain (GWG) in 2015, with uncertainty ranges in parentheses. Country-level SEs of the point estimates from the Demographic and Health Surveys data were accounted for using a multiple imputation approach.

**Figure 2:**
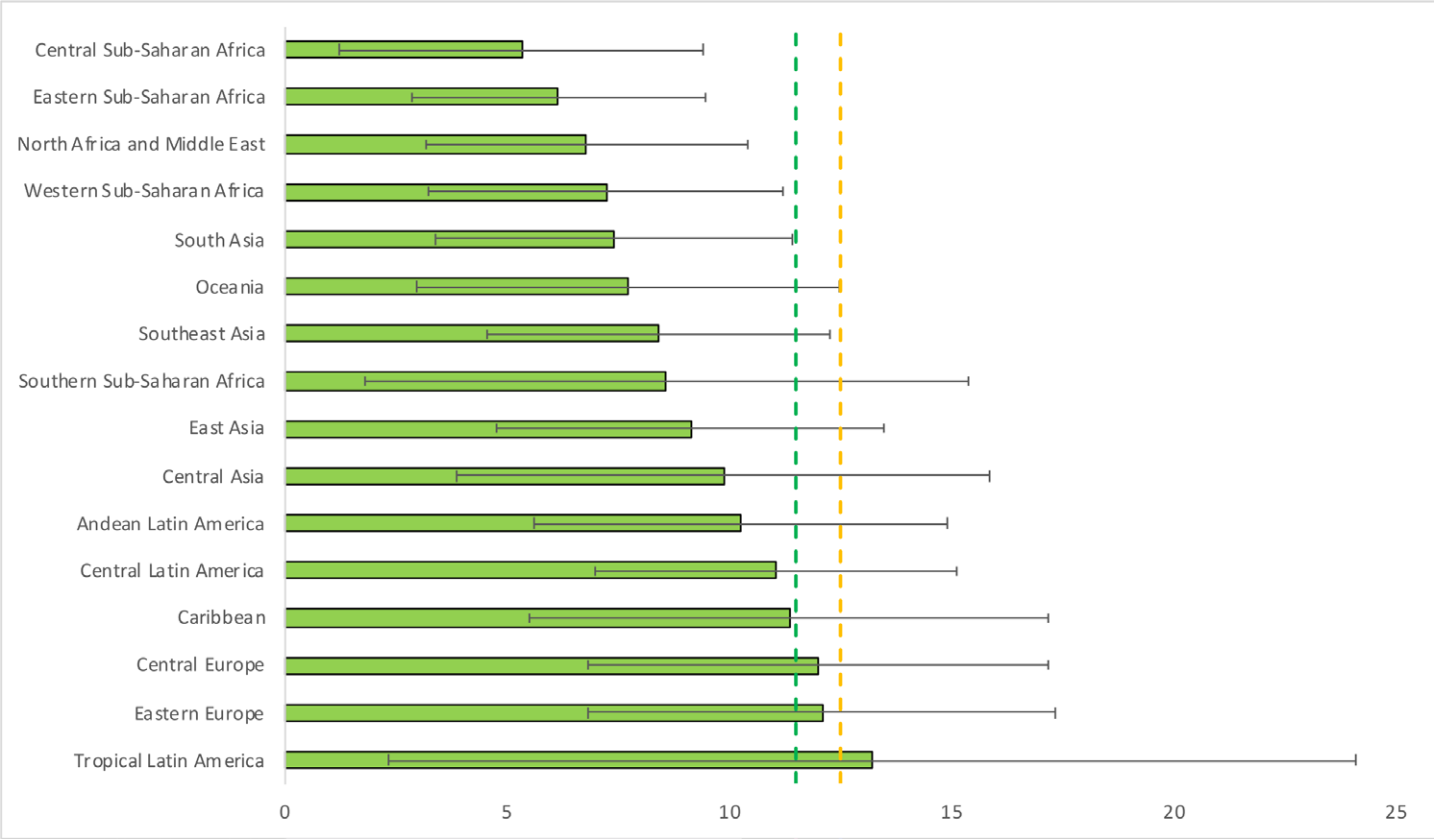
Estimated gestational weight gain in 2015 by Global Burden of Disease region. Two super-regions (North Africa and Middle East, and South Asia) do not have further regional divisions and are treated as their own regions. The green and orange dashed lines represent the minimum Institute of Medicine recommendations of total gestational weight gain for normal-weight (11.5 kg) and underweight (12.5 kg) women, respectively.

At the regional level, Tropical Latin America (13.2 kg (95% UR: 2.3, 24.1)), Eastern Europe (12.1 kg (95% UR: 6.9, 17.4)) and Central Europe (12.0 kg (95% UR: 6.8, 17.2)) were the only three regions that met the minimum IOM recommendation for normal-weight women, with Tropical Latin America also meeting the recommendation of 12.5 kg for underweight women. Central Sub-Saharan Africa (5.3 kg (95% UR: 1.3, 9.4)), Eastern Sub-Saharan Africa (6.2 kg (95% UR: 2.9, 9.5)) and Western Sub-Saharan Africa (7.2 kg (95% UR: 3.2, 11.2)) were the three regions with the lowest GWG estimates, meeting only 46%, 54% and 63% of the minimum recommendation for normal-weight women, respectively (table 2 and figure 2).

**Figure 3:**
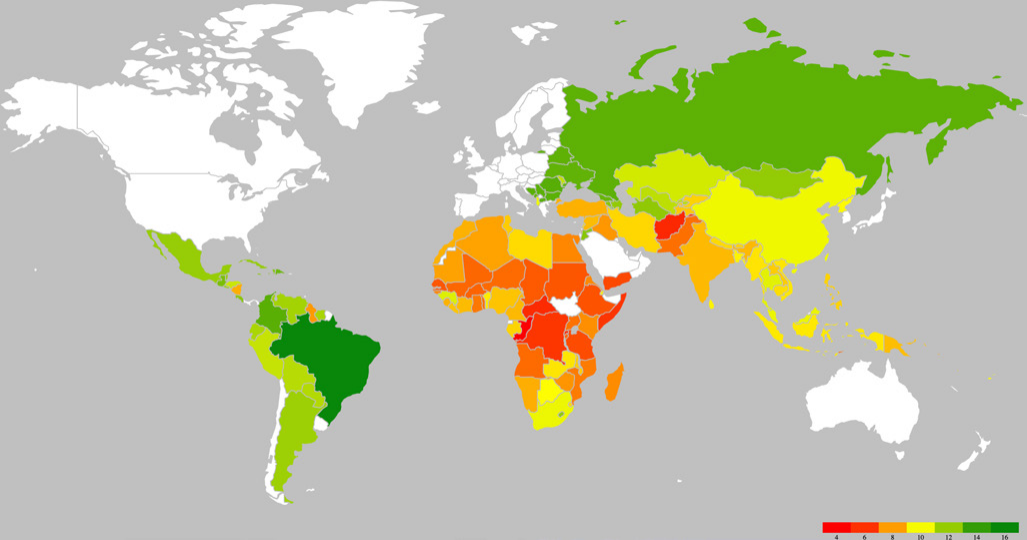
National estimates of mean gestational weight gain in low-income and middle-income countries. Different colours represent different magnitudes of the estimates. White colour represents high-income countries or low-income and middle-income countries for which estimates could not be computed (ie, South Sudan and Kosovo).

National estimates of the mean total GWG for each country in 2015 are summarised in figure 3, which shows the generally low levels of GWG in Africa, especially in Sub-Saharan Africa. The five countries with the highest GWG estimates were Brazil, Romania, Colombia, Bosnia and Herzegovina, and North Macedonia. The five countries with the lowest GWG estimates were Congo, Afghanistan, Rwanda, Central African Republic and the Democratic Republic of the Congo. Sixteen LMICs met the minimum GWG recommendation for normal-weight women, and only one country (Brazil: 14.0 kg (95% UR: 2.8, 25.1)) met the minimum recommendation for underweight women. The national estimates across years from the DHS and the estimates in 2015 from the hierarchical model are presented in online supplemental appendices 3 and 4.

10.1136/bmjgh-2020-003423.supp3 Supplementary data

10.1136/bmjgh-2020-003423.supp4 Supplementary data

## Discussion

We present the first effort to characterise and compare the average GWG levels across all low-income and middle-income countries and regions of the world. We found that Latin America and Caribbean, and Central Europe, Eastern Europe and Central Asia had the highest GWG estimates, whereas Sub-Saharan Africa and North Africa and Middle East had the lowest estimates. We reported a major burden of inadequate GWG in most low-income and middle-income regions and countries. With the exception of Latin America and Caribbean, all super-regions were below the minimum recommendation for normal-weight women, with Sub-Saharan Africa and North Africa and Middle East meeting less than 60% of the minimum recommendation.

Little is known about the levels of GWG in LMICs. Several previous efforts have been made to characterise GWG across countries. The INTERGROWTH-21st Project used cohort data from Brazil, China, India, Italy, Kenya, Oman, UK and the USA to provide population references of GWG. The project did not aim to describe GWG at the regional and national levels. Also, this project only included healthy women who were at low risk of adverse maternal and perinatal outcomes and were of normal weight in the first trimester.^40^ In a more recent cross-country analysis of GWG, Santos *et al*^19^ used data from the LifeCycle Consortium to construct GWG reference charts using pregnant women participating in 33 cohorts across Europe, North America and Oceania. The LifeCycle Consortium was not limited to healthy pregnancies and included preterm pregnancies and pregnancies with complications. It also incorporated underweight, overweight and obese participants. However, the LifeCycle Consortium relied on data from cohort studies that were generally not nationally representative of each country and may still have an over-representation of healthier pregnant women.^19^ Further, both the INTERGROWTH-21st Project and the LifeCycle Consortium had little representation of LMICs; the INTERGROWTH-21st Project included data from four LMICs (Brazil, China, India and Kenya), whereas only one LMIC (Ukraine) was represented in the LifeCycle Consortium. With the growing recognition of GWG as a target for preconceptional and antenatal care^26 27^ and the availability of tools to assess the adequacy of GWG,^1^ the lack of descriptive data in LMICs remains a major constraint.

Limited previous studies in selected LMICs suggest extremely high burden of inadequate GWG.^18 20–22^ Using the DHS data, Coffey^18^ estimated that the mean GWG in India and Sub-Saharan Africa was only 50% of the recommended amount for underweight women and 60% of the recommended amount for normal-weight women. Several clinic-based studies conducted in Sub-Saharan African countries, including Ethiopia,^22^ Ghana^21^ and Nigeria,^20^ also suggested that the weight gains for the majority of pregnant women were below the minimum recommendations. A recent systematic review by Asefa *et al*^23^ examined the distribution of GWG in Sub-Saharan Africa and found that the percentage of inadequate GWG was greater than 58% in all included low-income Sub-Saharan countries. Our estimates are consistent with these prior estimates and again highlight the particularly low levels of GWG in Africa and South Asia. Our findings also suggest a considerable gap in total GWG between LMICs and high-income countries. All cohorts in the LifeCycle Consortium (from Europe, Oceania and North America) have met the minimum recommendation for normal-weight women, with the vast majority of them also meeting the minimum recommendation for underweight women. The median total GWG of a full-term pregnancy of 40 weeks in the LifeCycle Consortium was 14.0 kg (IQR: 11.0–17.9). In contrast, we estimated that only 16 LMICs met the minimum recommendation for normal-weight women.

GWG is strongly dependent on modifiable maternal factors, including nutritional status, dietary intake, physical activity and pre-existing health conditions.^1 41^ Several factors may have contributed to the high burden of inadequate GWG in LMICs. Inadequate dietary intake, food insecurity and maternal malnutrition (eg, iron, vitamin A and zinc deficiencies) disproportionately affect pregnant women in LMICs, especially in Sub-Saharan Africa.^42^ Early marriage and adolescent pregnancy, much more prevalent in Sub-Saharan Africa and South Asia,^43^ may also have played a role. In resource-constrained settings, women who become pregnant early or during adolescence may not have finished growing themselves or have accumulated sufficient socioeconomic resources to ensure a healthy pregnancy.^44^ It was beyond the scope of this study to quantitatively evaluate the relative contributions of various maternal risk factors to GWG at the population level. Future studies should assess how different maternal characteristics explain inadequate or excessive GWG in various national and regional contexts, which will guide the design of antenatal intervention programmes needed for each setting.

Data from the DHS Program have the key advantage of being nationally representative, which provided us with the unique opportunity to compute national estimates. This study also has several limitations associated with the use of DHS data. First, due to the cross-sectional nature of the DHS, only one weight was available for each pregnant woman, which did not allow us to estimate GWG at the individual level or the percentage of women who failed to meet the IOM recommendation. The methodology of estimating the mean total GWG using cross-sectional DHS data has been previously used for India and Sub-Saharan Africa.^18^ Because the hierarchical model’s input data are themselves model estimates with some uncertainty, the final estimates have higher levels of uncertainty than they would have if gestational weights were observed at the individual level. Nonetheless, we accounted for this additional source of error analytically using a multiple imputation approach. Even the upper limits of the UR for South Asia, North Africa and Middle East, and much of Sub-Saharan Africa (except Southern Sub-Saharan Africa) are below the minimum recommendation. Second, no data were available on prepregnancy weight. This limitation precluded the estimation of GWG adequacy levels based on the IOM guidelines, which had different recommendations based on prepregnancy BMI categories.^1^ The burden of excessive GWG, in particular, was likely masked by the low average GWG levels and thus could not be determined. Future work should seek to ascertain inadequate and excessive GWG at the individual level. Third, the DHS data were available for approximately half of LMICs and were notably lacking for countries in Europe, Oceania and East Asia. Further work using nationally representative data from countries not represented or under-represented is warranted to estimate the national GWG levels.

## Conclusion

We present the national and regional estimates of GWG in low-income and middle-income countries and regions using nationally representative data. Findings from this study provide a much-needed evidence base to inform healthcare planning and intervention developments targeting GWG in resource-limited settings. This study also highlights the large data gaps and draws attention to the scarcity of longitudinal monitoring systems of gestational weight in LMICs. Future efforts to characterise national, regional and global patterns of GWG should take advantage of data that have information on prepregnancy weight as well as longitudinally collected weight data.
