# Supplementary material for: Gestational weight gain in low-income and middle-income countries: a modelling analysis using nationally representative data
Source: BMJ Glob Health. 2020 Nov 11;5(11):e003423. doi: 10.1136/bmjgh-2020-003423 (PMC7661366; doi:10.1136/bmjgh-2020-003423)
Supplement: Supplementary data [file bmjgh-2020-003423supp001.pdf]

Appendix 1
Gestational weight gain estimates from the DHS datasets included in the analysis

Country	Year of DHS survey	Point estimate (kg)	Lower limit of 95% confidence interval (kg)	Upper limit of 95% confidence interval (kg)
Albania	2017	18.9	13.1	24.7
Albania	2008	10.8	2.4	19.2
Armenia	2015	25.9	16.9	35.0
Armenia	2005	10.5	-0.8	21.8
Armenia	2000	12.6	4.9	20.2
Azerbaijan	2006	11.3	5.7	16.9
Bangladesh	2014	9.9	7.7	12.1
Bangladesh	2011	8.9	6.5	11.2
Bangladesh	2007	11.7	8.9	14.4
Bangladesh	2004	7.7	5.6	9.8
Bangladesh	1999	7.1	4.6	9.6
Bangladesh	1996	7.6	4.7	10.5
Benin	2017	8.3	5.2	11.4
Benin	2011	5.4	3.0	7.8
Benin	2006	6.3	4.4	8.2
Benin	2001	8.4	6.2	10.7
Benin	1996	11.6	7.6	15.5
Bolivia	2008	8.8	5.9	11.6
Bolivia	2003	13.6	10.7	16.5
Bolivia	1998	4.9	-0.1	9.8
Bolivia	1994	12.0	6.7	17.2
Brazil	1996	14.9	7.5	22.4
Burkina Faso	2010	7.4	5.2	9.6
Burkina Faso	2003	4.8	2.9	6.7
Burkina Faso	1998	7.1	4.4	9.8
Burkina Faso	1993	4.5	1.8	7.2
Burundi	2016	7.2	3.2	11.1
Burundi	2010	6.6	4.1	9.2
Cambodia	2014	10.2	7.6	12.9
Cambodia	2010	8.9	5.8	12.1
Cambodia	2005	10.3	8.1	12.4
Cambodia	2000	5.8	3.9	7.8
Cameroon	2011	8.5	4.9	12.1
Cameroon	2004	10.3	6.5	14.2
Cameroon	1998	4.8	-2.0	11.6
Central African Republic	1994	4.7	1.1	8.3
Chad	2014	9.1	7.0	11.3
Chad	2004	5.1	2.4	7.8
Chad	1996	5.5	2.9	8.2
Colombia	2010	13.7	11.0	16.4
Colombia	2005	14.8	12.2	17.4
Colombia	2000	10.4	6.9	14.0
Colombia	1995	11.9	8.7	15.0
Comoros	2012	14.1	8.3	19.9
Comoros	1996	9.6	-0.4	19.6
Congo	2011	14.5	6.6	22.4
Congo	2005	2.1	-1.7	6.0
Democratic Republic of the Congo	2013	5.6	3.1	8.0
Democratic Republic of the Congo	2007	5.2	1.6	8.8
Cote d'Ivoire	2011	2.1	-2.1	6.3
Cote d'Ivoire	1998	10.0	4.6	15.3
Cote d'Ivoire	1994	6.8	3.0	10.6
Dominican Republic	2013	13.2	7.5	19.0
Dominican Republic	1996	11.0	6.4	15.6
Dominican Republic	1991	13.0	7.7	18.3
Egypt	2014	4.3	2.1	6.6
Egypt	2008	5.9	3.5	8.2
Egypt	2005	3.2	0.7	5.6
Egypt	2003	9.4	5.7	13.1
Egypt	2000	6.8	4.1	9.5
Egypt	1995	9.4	5.6	13.2

Egypt	1992	6.6	2.5	10.8
Eswatini	2006	8.0	1.4	14.7
Ethiopia	2016	6.9	4.5	9.2
Ethiopia	2011	6.0	3.9	8.2
Ethiopia	2005	4.4	1.0	7.8
Ethiopia	2000	4.8	2.8	6.7
Gabon	2012	6.2	-1.1	13.5
Gabon	2000	8.7	2.8	14.6
The Gambia	2013	8.4	3.8	13.1
Ghana	2014	1.3	-3.8	6.4
Ghana	2008	6.1	2.0	10.1
Ghana	2003	4.1	-0.1	8.2
Ghana	1998	10.0	4.9	15.1
Ghana	1993	4.3	-0.4	9.0
Guatemala	1998	12.7	7.7	17.8
Guatemala	1995	9.0	6.1	11.8
Guinea	2018	5.4	0.9	9.9
Guinea	2012	7.8	4.4	11.3
Guinea	2005	8.8	5.2	12.3
Guinea	1999	10.3	7.4	13.1
Guyana	2009	6.9	-5.0	18.8
Haiti	2016	13.5	7.8	19.1
Haiti	2012	11.1	6.8	15.4
Haiti	2005	14.5	9.2	19.8
Haiti	2000	9.8	4.4	15.1
Haiti	1994	9.5	4.4	14.6
Honduras	2011	8.6	5.6	11.7
Honduras	2005	9.9	7.0	12.8
India	2015	8.2	7.7	8.8
India	2005	7.9	7.0	8.8
India	1998	6.8	6.0	7.7
Jordan	2017	8.7	4.1	13.3
Jordan	2012	14.6	6.5	22.8
Jordan	2009	6.1	0.1	12.1
Jordan	2007	8.4	3.7	13.1
Jordan	2002	11.5	7.6	15.3
Jordan	1997	14.7	10.4	18.9
Kazakhstan	1999	7.7	-4.1	19.4
Kazakhstan	1995	13.7	6.8	20.7
Kenya	2014	4.8	1.0	8.6
Kenya	2008	4.0	-1.8	9.8
Kenya	2003	6.8	2.9	10.7
Kenya	1998	6.2	2.0	10.5
Kenya	1993	6.7	3.0	10.3
Kyrgyz Republic	2012	9.9	6.4	13.5
Kyrgyz Republic	1997	8.4	1.3	15.4
Lesotho	2014	13.6	3.7	23.6
Lesotho	2009	20.4	6.5	34.3
Lesotho	2004	7.2	-0.1	14.5
Liberia	2013	9.3	5.8	12.7
Liberia	2007	7.9	4.0	11.7
Madagascar	2008	5.7	2.9	8.5
Madagascar	2003	8.3	5.2	11.5
Madagascar	1997	5.3	1.0	9.6
Malawi	2015	8.4	5.8	10.9
Malawi	2010	6.0	3.6	8.4
Malawi	2004	8.5	6.8	10.3
Malawi	2000	7.1	4.7	9.6
Malawi	1992	7.2	3.4	10.9
Maldives	2016	9.0	-0.1	18.1
Maldives	2009	11.9	7.4	16.4
Mali	2018	1.9	-2.8	6.5
Mali	2012	8.0	4.4	11.7
Mali	2006	5.0	3.2	6.8
Mali	2001	6.3	4.4	8.2
Mali	1995	5.1	2.2	7.9

Moldova	2005	10.0	1.6	18.4
Morocco	2003	9.0	6.0	11.9
Morocco	1992	6.1	1.5	10.8
Mozambique	2011	5.7	3.5	7.8
Mozambique	2003	3.6	1.7	5.4
Mozambique	1997	9.5	4.5	14.4
Myanmar	2015	8.0	4.6	11.5
Namibia	2013	7.8	2.3	13.3
Namibia	2006	9.8	5.5	14.2
Namibia	1992	5.4	-0.8	11.6
Nepal	2016	8.5	3.9	13.1
Nepal	2011	6.0	1.6	10.4
Nepal	2006	8.7	5.8	11.6
Nepal	2001	7.5	5.6	9.4
Nepal	1996	6.4	4.0	8.9
Nicaragua	2001	5.4	2.0	8.9
Nicaragua	1998	8.0	4.6	11.3
Niger	2012	7.1	4.1	10.1
Niger	2006	6.4	3.4	9.3
Niger	1998	6.7	4.1	9.2
Niger	1992	6.4	3.6	9.2
Nigeria	2018	8.7	6.1	11.3
Nigeria	2013	7.4	5.8	9.0
Nigeria	2008	6.1	4.5	7.7
Nigeria	2003	9.8	6.4	13.3
Pakistan	2017	7.2	0.6	13.8
Pakistan	2012	6.4	1.7	11.0
Peru	2012	8.2	5.3	11.0
Peru	2011	10.2	7.1	13.3
Peru	2010	11.2	8.1	14.3
Peru	2009	11.3	8.0	14.6
Peru	2006	12.5	10.1	14.9
Peru	2000	11.9	9.3	14.5
Peru	1996	6.8	3.6	9.9
Peru	1991	8.8	5.5	12.2
Rwanda	2014	5.0	1.2	8.8
Rwanda	2010	4.4	1.5	7.4
Rwanda	2005	2.4	-0.6	5.4
Rwanda	2000	5.9	3.7	8.1
Sao Tome and Principe	2008	5.8	-0.2	11.9
Senegal	2010	8.4	2.6	14.3
Senegal	2005	4.3	-0.4	9.1
Senegal	1992	6.3	2.9	9.7
Sierra Leone	2013	4.0	0.6	7.5
Sierra Leone	2008	7.1	3.0	11.2
South Africa	2016	15.3	1.9	28.8
Tajikistan	2017	12.1	9.2	15.1
Tajikistan	2012	8.6	5.9	11.4
Tanzania	2015	7.3	4.3	10.3
Tanzania	2010	6.8	3.8	9.9
Tanzania	2004	5.3	2.9	7.7
Tanzania	1996	4.2	-0.5	8.9
Tanzania	1991	6.3	3.9	8.6
Timor-Leste	2016	7.3	4.3	10.3
Timor-Leste	2009	6.0	3.7	8.3
Togo	2013	5.2	1.1	9.3
Togo	1998	6.1	3.5	8.7
Turkey	2013	12.3	4.8	19.7
Turkey	2008	11.5	5.2	17.9
Turkey	2003	5.2	0.5	9.8
Turkey	1998	8.9	2.2	15.7
Turkey	1993	5.8	-1.0	12.7
Uganda	2016	8.9	4.5	13.2
Uganda	2011	5.6	1.5	9.6
Uganda	2006	4.8	1.6	8.0
Uganda	2000	6.8	3.3	10.3

Uganda	1995	5.7	3.3	8.1
Uzbekistan	1996	9.6	3.7	15.4
Yemen	2013	5.8	3.9	7.6
Zambia	2013	8.0	5.5	10.5
Zambia	2007	9.1	6.1	12.0
Zambia	2001	7.9	5.1	10.6
Zambia	1996	8.6	4.8	12.5
Zambia	1992	7.6	4.5	10.6
Zimbabwe	2015	9.4	5.8	13.0
Zimbabwe	2010	7.0	3.9	10.1
Zimbabwe	2005	9.3	5.7	13.0
Zimbabwe	1999	6.0	1.6	10.4
Zimbabwe	1994	7.8	0.9	14.7
