# Supplementary material for: Gestational weight gain in low-income and middle-income countries: a modelling analysis using nationally representative data
Source: BMJ Glob Health. 2020 Nov 11;5(11):e003423. doi: 10.1136/bmjgh-2020-003423 (PMC7661366; doi:10.1136/bmjgh-2020-003423)
Supplement: Supplementary data [file bmjgh-2020-003423supp002.pdf]

Appendix 2
Country-level predictors considered for the hierarchical model and their sources

Country-level predictor	Source	Decision
Neonatal mortality rate (deaths per 1000 live births)	United Nations Inter-agency Group for Child Mortality Estimation (UN IGME) http://www.childmortality.org/	Dropped during variable selection
Low birthweight rate (%)	UNICEF Data Warehouse http://data.unicef.org/ World Bank Open Data https://data.worldbank.org/	Dropped due to high level of missingness
Proportion of women receiving four or more antenatal care visits (%)	UNICEF Data Warehouse http://data.unicef.org/	Dropped due to high level of missingness
Cesarean section rate (%)	UNICEF Data Warehouse http://data.unicef.org/	Dropped due to high level of missingness
Mean adult female body mass index (kg/m^2)	NCD Risk Factor Collaboration (NCD-RisC) http://ncdrisc.org/	Retained in the final model
Gross domestic product per capita (current US dollars)	World Bank Open Data https://data.worldbank.org/	Retained in the final model
GINI index	World Bank Open Data https://data.worldbank.org/ Federal Reserve Economic Data https://fred.stlouisfed.org/	Dropped due to high level of missingness
Human Development Index	United Nations Development Programme (UNDP) Human Development Reports http://www.hdr.undp.org/	Dropped due to multicollinearity
Total fertility rate (births per woman)	World Bank Open Data https://data.worldbank.org/	Retained in the final model
Adolescent fertility rate (births per 1000 women)	World Bank Open Data https://data.worldbank.org/	Dropped during variable selection
Percentage of adolescents aged 15 to 19 years out of all women of reproductive age (15 to 49 years)	United Nations World Population Prospects 2019 https://population.un.org/wpp/	Dropped during variable selection
Adult female literacy rate (%)	World Bank Open Data https://data.worldbank.org/	Dropped due to high level of missingness
