# Supplementary material for: Gestational weight gain in low-income and middle-income countries: a modelling analysis using nationally representative data
Source: BMJ Glob Health. 2020 Nov 11;5(11):e003423. doi: 10.1136/bmjgh-2020-003423 (PMC7661366; doi:10.1136/bmjgh-2020-003423)
Supplement: Supplementary data [file bmjgh-2020-003423supp003.pdf]

Appendix 3
Gestational weight gain estimated for the year 2015 using hierarchical modeling

Country	Point estimate (kg)	Lower limit of 95% uncertainty range (kg)	Upper limit of 95% uncertainty range (kg)
Afghanistan	4.8	0.4	9.2
Albania	9.2	1.3	17.1
Algeria	7.1	3.0	11.3
Angola	6.1	2.2	10.0
Argentina	10.8	7.2	14.5
Armenia	11.3	-2.5	25.1
Azerbaijan	11.1	6.3	15.8
Bangladesh	8.6	4.0	13.2
Belarus	12.1	7.4	16.9
Belize	11.0	7.1	14.9
Benin	8.5	3.0	13.9
Bhutan	8.0	4.6	11.5
Bolivia	10.3	3.7	16.9
Bosnia and Herzegovina	12.2	7.0	17.4
Botswana	8.8	5.3	12.3
Brazil	14.0	2.8	25.1
Bulgaria	12.2	7.0	17.3
Burkina Faso	6.0	2.0	9.9
Burundi	6.0	1.4	10.7
Cabo Verde	9.0	5.5	12.5
Cambodia	8.2	3.8	12.7
Cameroon	7.5	1.9	13.1
Central African Republic	4.9	-2.4	12.2
Chad	5.8	1.2	10.4
China	9.1	5.2	13.1
Colombia	12.2	7.0	17.5
Comoros	10.2	-0.7	21.1
Congo	4.1	-6.7	14.9
Democratic Republic of the Congo	5.1	0.9	9.2
Costa Rica	11.3	7.8	14.7
Cote d'Ivoire	7.6	1.5	13.7
Cuba	11.0	7.4	14.7
Djibouti	8.5	5.1	11.8
Dominica	11.2	7.8	14.5
Dominican Republic	11.9	5.5	18.2
Ecuador	10.6	7.3	13.9
Egypt	6.6	2.0	11.2
El Salvador	10.9	7.6	14.2
Equatorial Guinea	7.3	3.1	11.6
Eritrea	6.6	3.2	10.1
Eswatini	8.1	1.1	15.1
Ethiopia	5.6	1.5	9.8
Fiji	8.9	3.8	14.1
Gabon	8.0	3.4	12.7
Georgia	11.9	7.5	16.4
Ghana	6.3	1.1	11.6
Grenada	11.1	7.7	14.5
Guatemala	11.6	5.5	17.6
Guinea	9.5	5.3	13.7
Guinea-Bissau	6.6	3.6	9.6
Guyana	6.9	-4.1	17.9
Haiti	11.4	4.1	18.7
Honduras	9.9	5.3	14.5
India	7.5	4.5	10.6
Indonesia	8.4	4.8	12.0
Iran	8.1	4.0	12.2
Iraq	6.9	2.7	11.1
Jamaica	11.2	7.7	14.7
Jordan	11.3	3.9	18.7
Kazakhstan	9.8	2.0	17.5
Kenya	6.8	0.8	12.7

Kiribati	8.3	1.2	15.5
Kosovo[1]	..	..	..
Kyrgyz Republic	8.0	-2.0	18.0
Laos	7.9	4.4	11.4
Lebanon	8.2	4.0	12.3
Lesotho	11.1	-2.6	24.8
Liberia	7.8	3.5	12.0
Libya	8.2	4.4	11.9
Madagascar	6.7	2.4	11.0
Malawi	7.9	4.1	11.7
Malaysia	9.2	5.2	13.3
Maldives	11.7	3.2	20.2
Mali	6.0	2.4	9.6
Marshall Islands	8.1	1.4	14.8
Mauritania	7.1	3.6	10.6
Mauritius	9.8	5.8	13.8
Mexico	11.0	7.6	14.4
Micronesia	8.9	2.1	15.7
Moldova	10.1	2.2	17.9
Mongolia	11.1	6.4	15.7
Montenegro	12.1	7.1	17.0
Morocco	7.4	1.0	13.8
Mozambique	6.4	0.9	11.9
Myanmar	8.3	3.6	13.0
Namibia	7.3	0.5	14.1
Nauru	9.6	2.3	16.8
Nepal	8.1	4.4	11.9
Nicaragua	7.3	1.9	12.8
Niger	6.1	2.1	10.1
Nigeria	7.7	2.2	13.3
North Korea	8.7	5.0	12.5
North Macedonia	12.2	7.4	17.0
Pakistan	6.2	-1.7	14.1
Papua New Guinea	7.6	3.3	12.0
Paraguay	10.4	7.1	13.7
Peru	10.0	4.1	15.9
Philippines	8.0	4.5	11.6
Romania	12.3	7.3	17.3
Russia	12.2	7.1	17.2
Rwanda	4.9	0.0	9.8
Samoa	8.7	0.0	17.3
Sao Tome and Principe	5.7	-3.5	14.9
Senegal	5.7	-0.4	11.8
Serbia	12.2	7.1	17.3
Sierra Leone	7.4	2.2	12.6
Solomon Islands	7.1	1.9	12.3
Somalia	5.1	1.6	8.6
South Africa	9.2	1.4	17.0
South Sudan[1]	..	..	..
Sri Lanka	8.6	5.0	12.2
St. Lucia	11.9	8.3	15.4
St. Vincent and the Grenadines	11.1	7.8	14.4
Sudan	5.7	1.4	10.0
Suriname	10.6	7.2	14.1
Syria	7.6	3.8	11.4
Tajikistan	7.9	0.5	15.2
Tanzania	5.5	1.4	9.6
Thailand	9.4	5.6	13.2
The Gambia	6.5	1.7	11.2
Timor-Leste	6.1	1.3	11.0
Togo	6.2	1.7	10.7
Tonga	8.9	0.6	17.3
Tunisia	7.9	3.9	11.9
Turkey	7.4	0.5	14.2
Turkmenistan	11.1	6.2	16.0
Tuvalu	9.1	2.2	16.0

Uganda	6.2	1.1	11.3
Ukraine	12.1	7.5	16.7
Uzbekistan	10.2	3.3	17.1
Vanuatu	7.6	2.8	12.4
Venezuela	10.7	6.8	14.6
Vietnam	8.4	4.7	12.1
West Bank and Gaza	6.8	2.6	11.0
Yemen	5.5	0.0	11.0
Zambia	8.4	4.0	12.7
Zimbabwe	6.9	1.1	12.6

[1]Estimates for South Sudan and Kosovo could not be computed due to the lack of sufficient covariate data in 2015.
