# Supplementary figures and images for: Gestational weight gain in low-income and middle-income countries: a modelling analysis using nationally representative data
Source: BMJ Glob Health. 2020 Nov 11;5(11):e003423. doi: 10.1136/bmjgh-2020-003423 (PMC7661366; doi:10.1136/bmjgh-2020-003423)

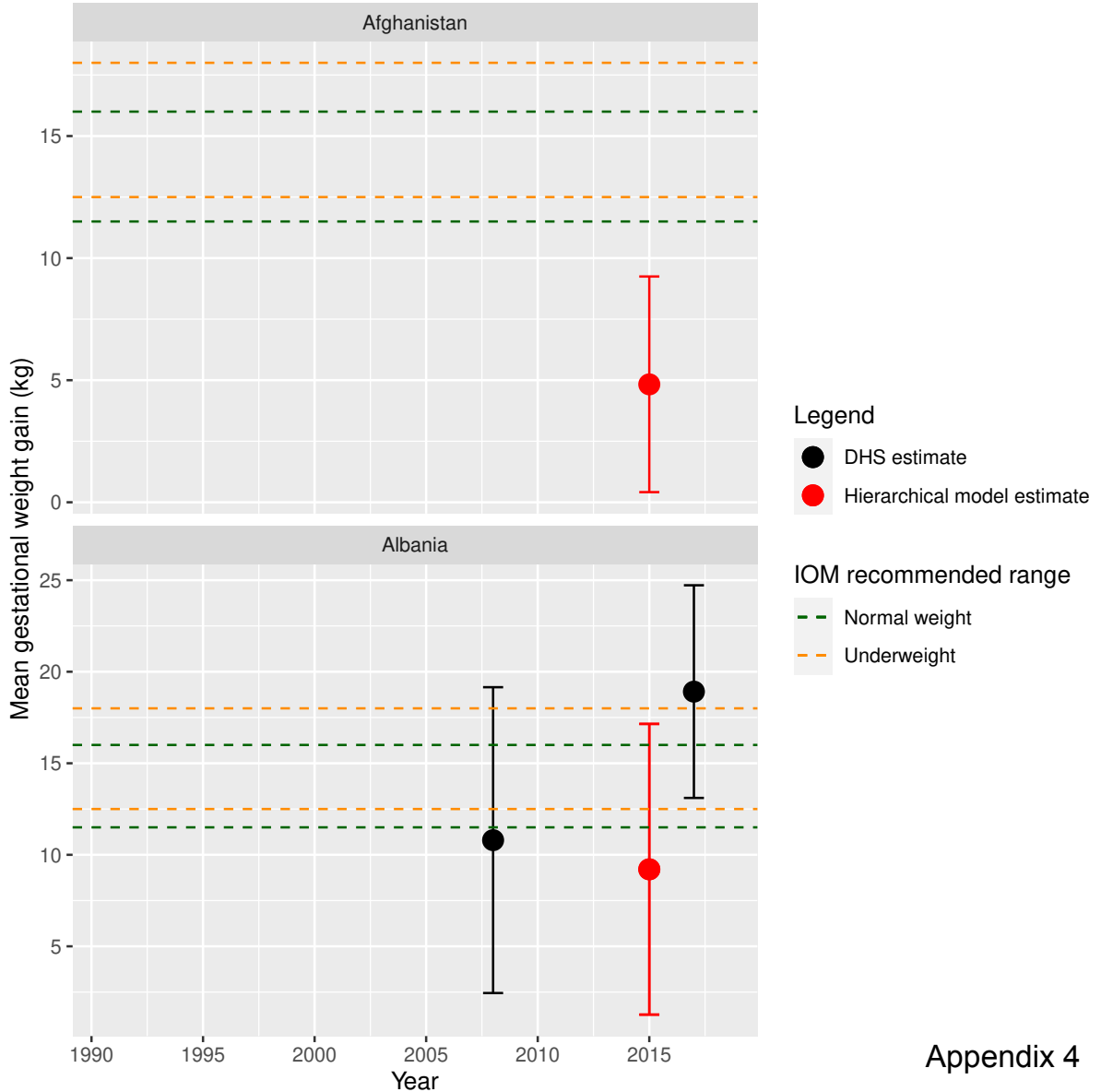

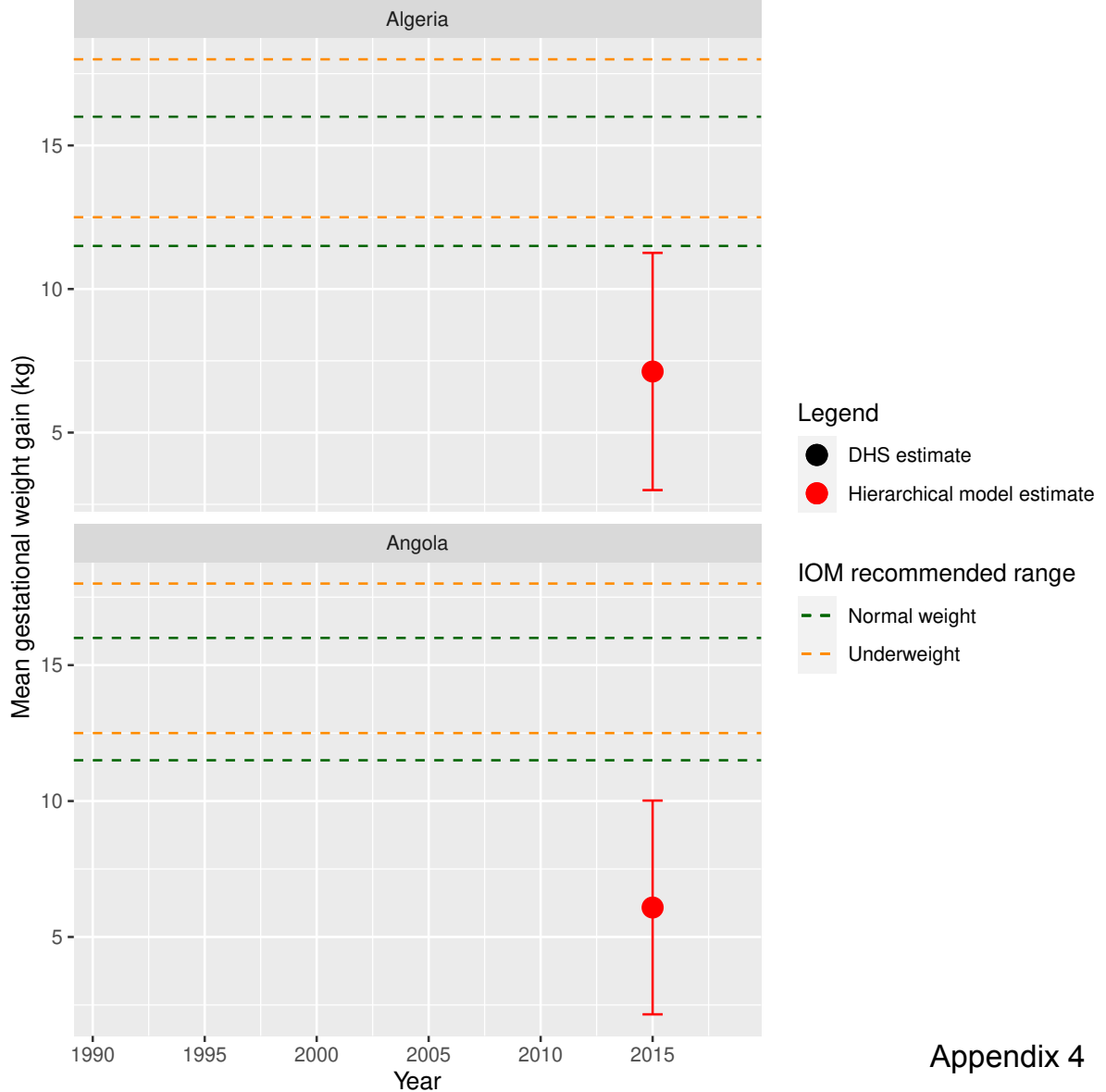

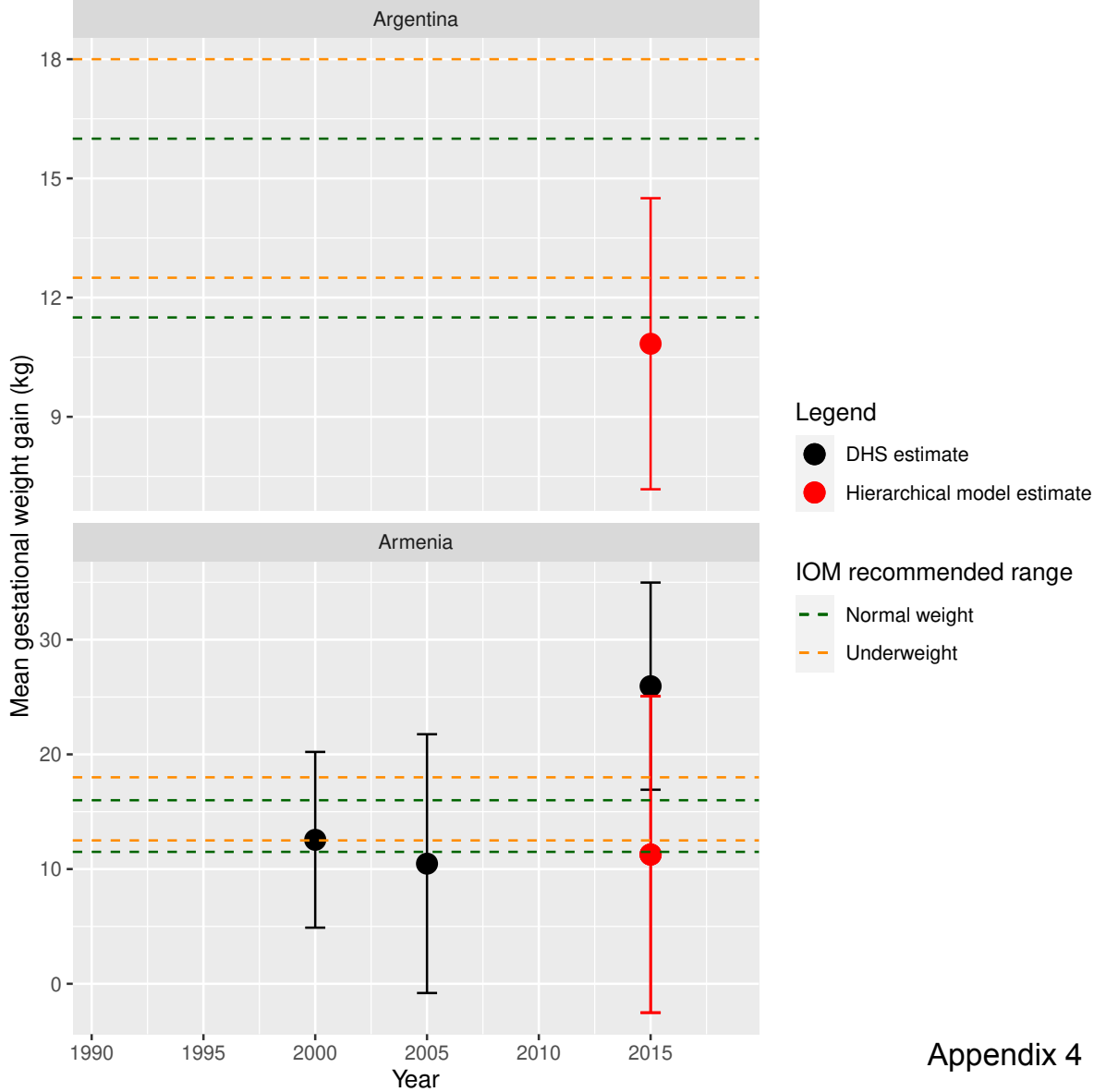

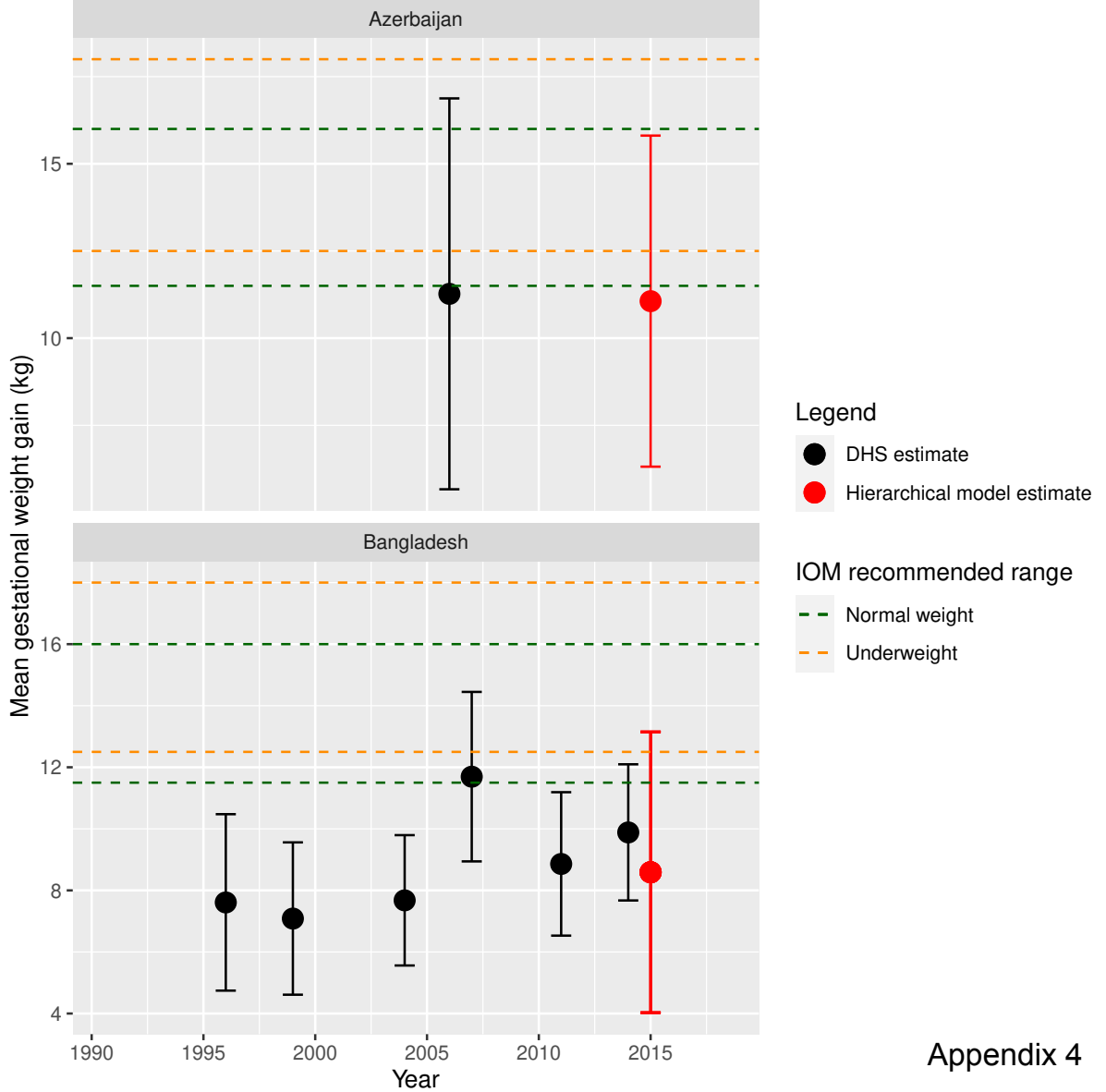

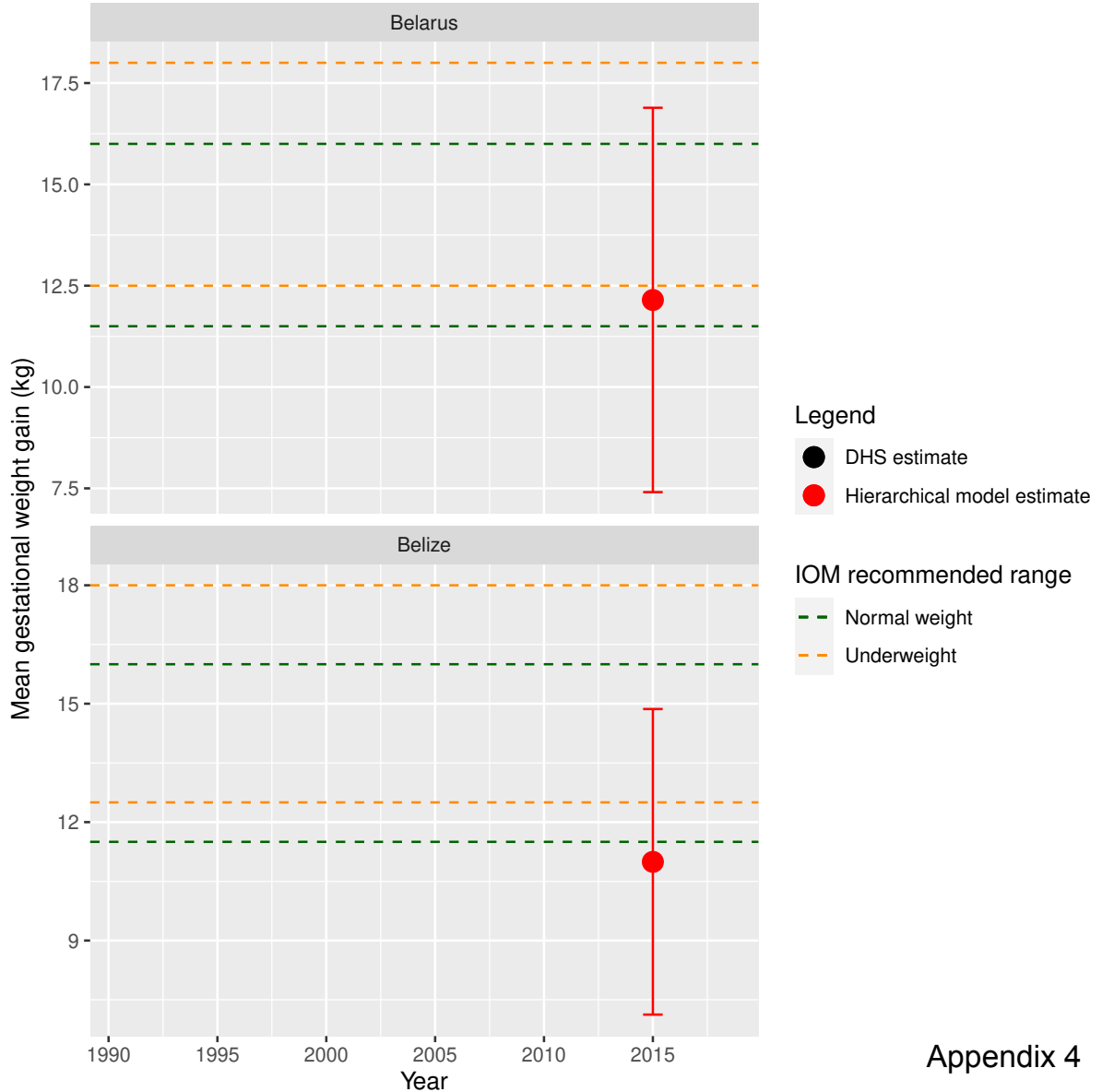

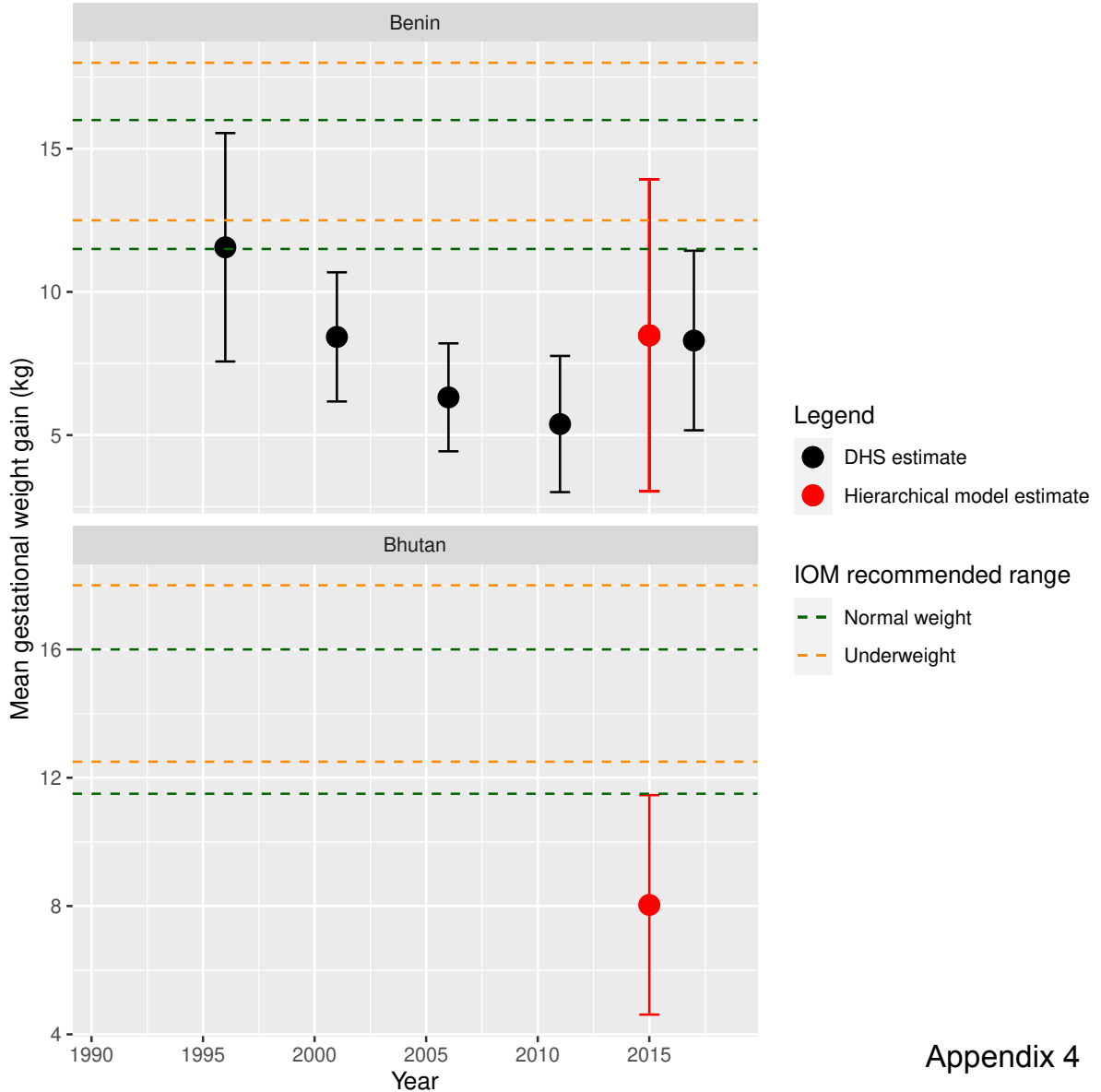

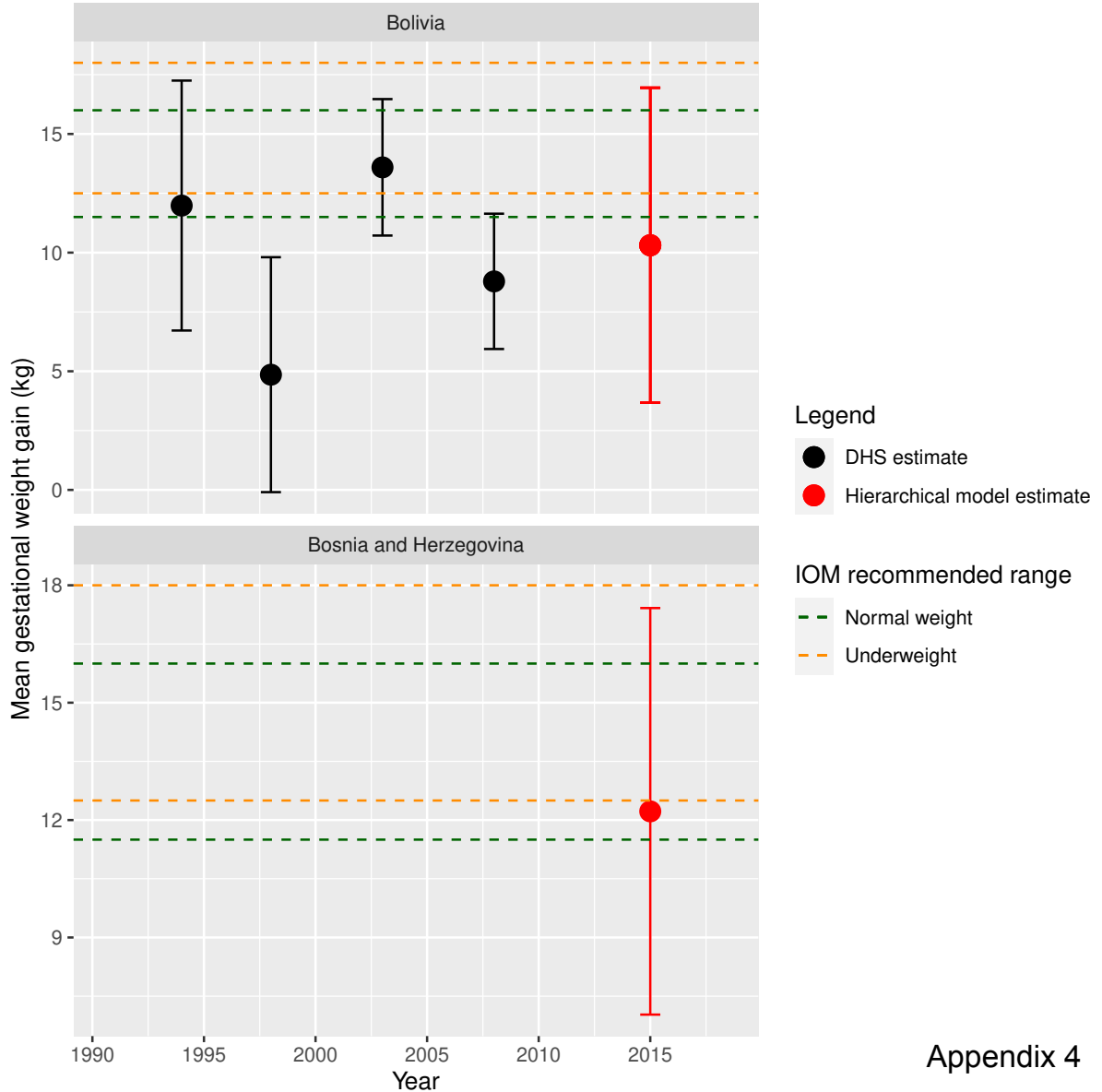

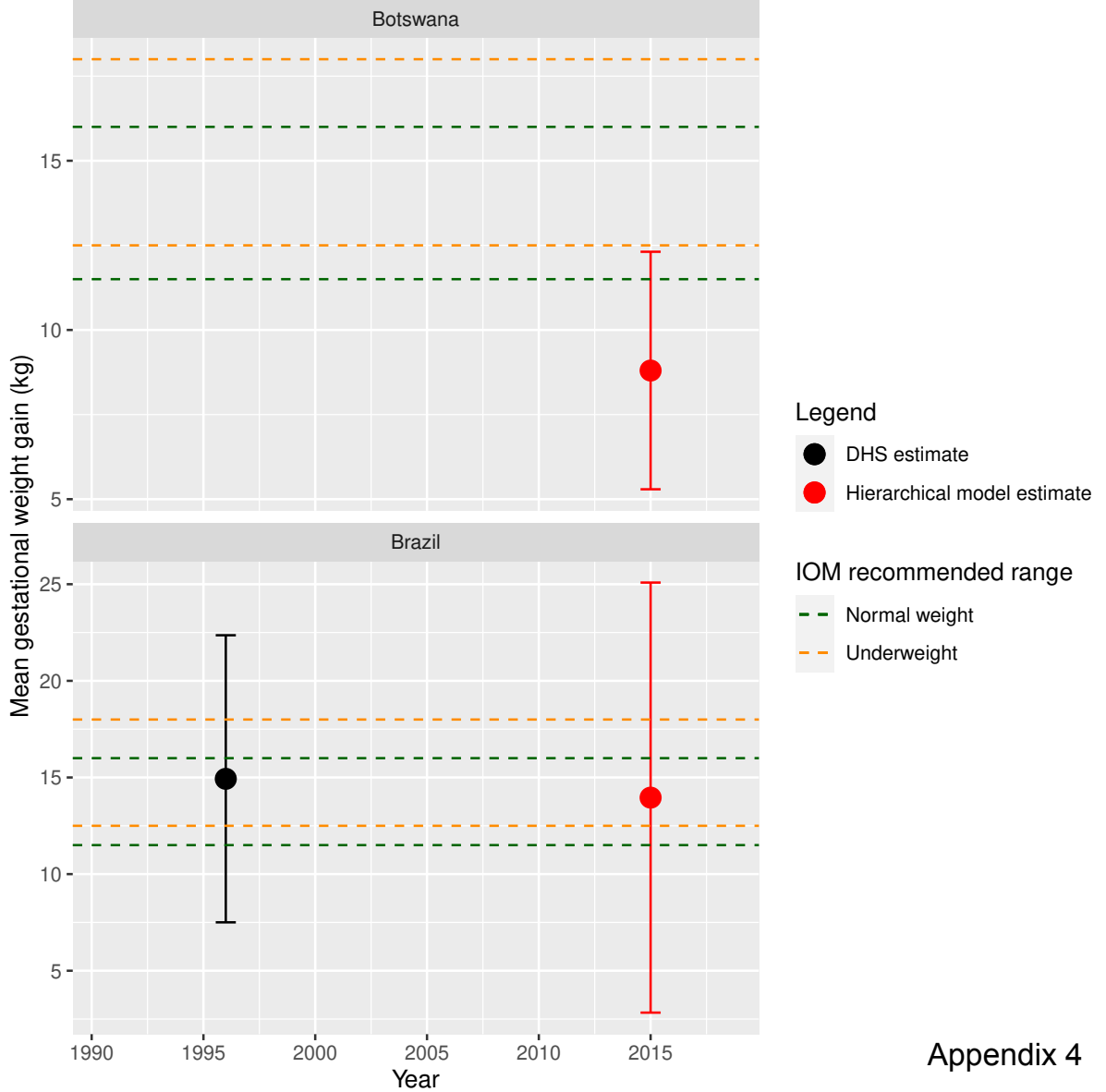

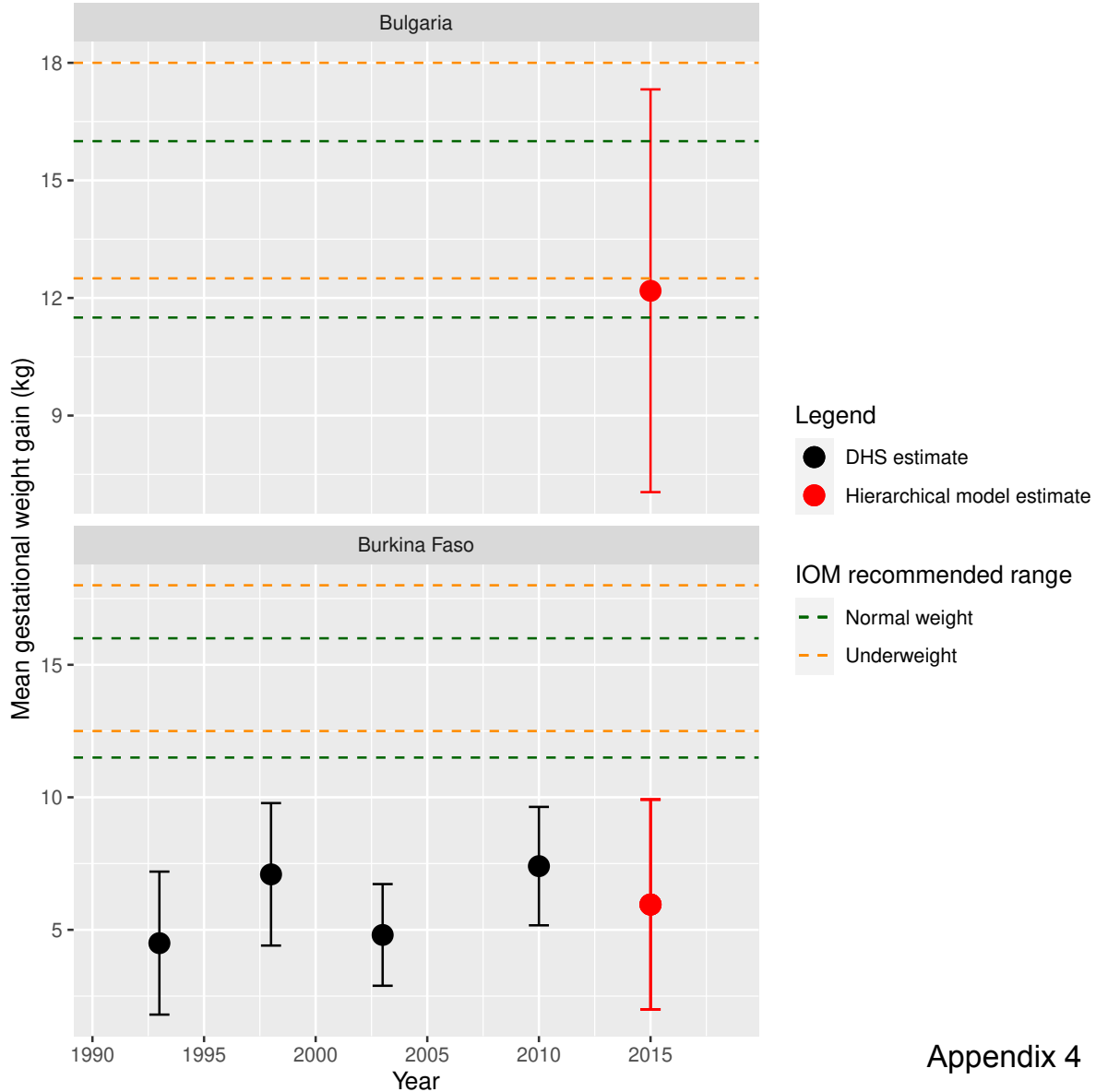

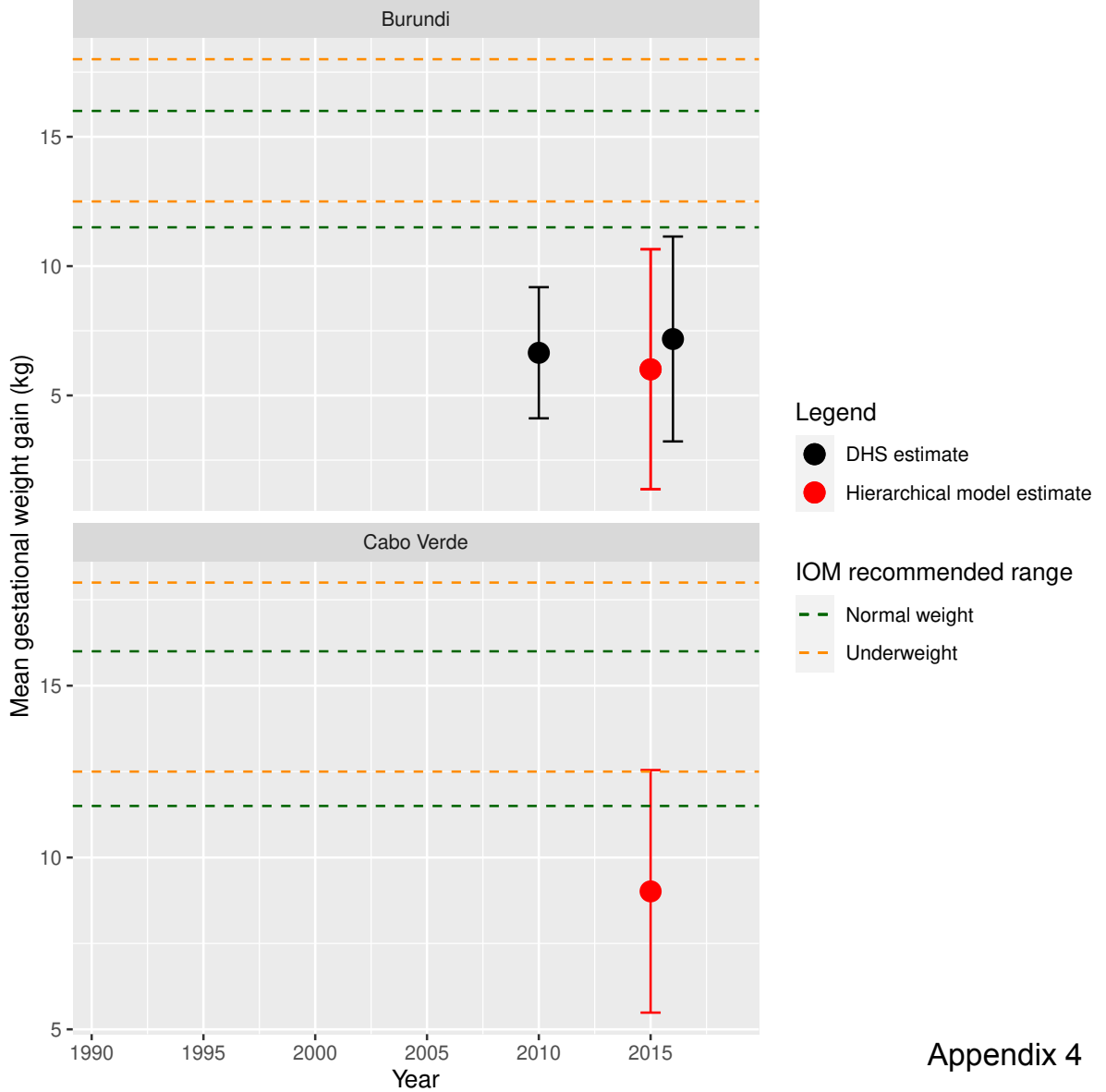

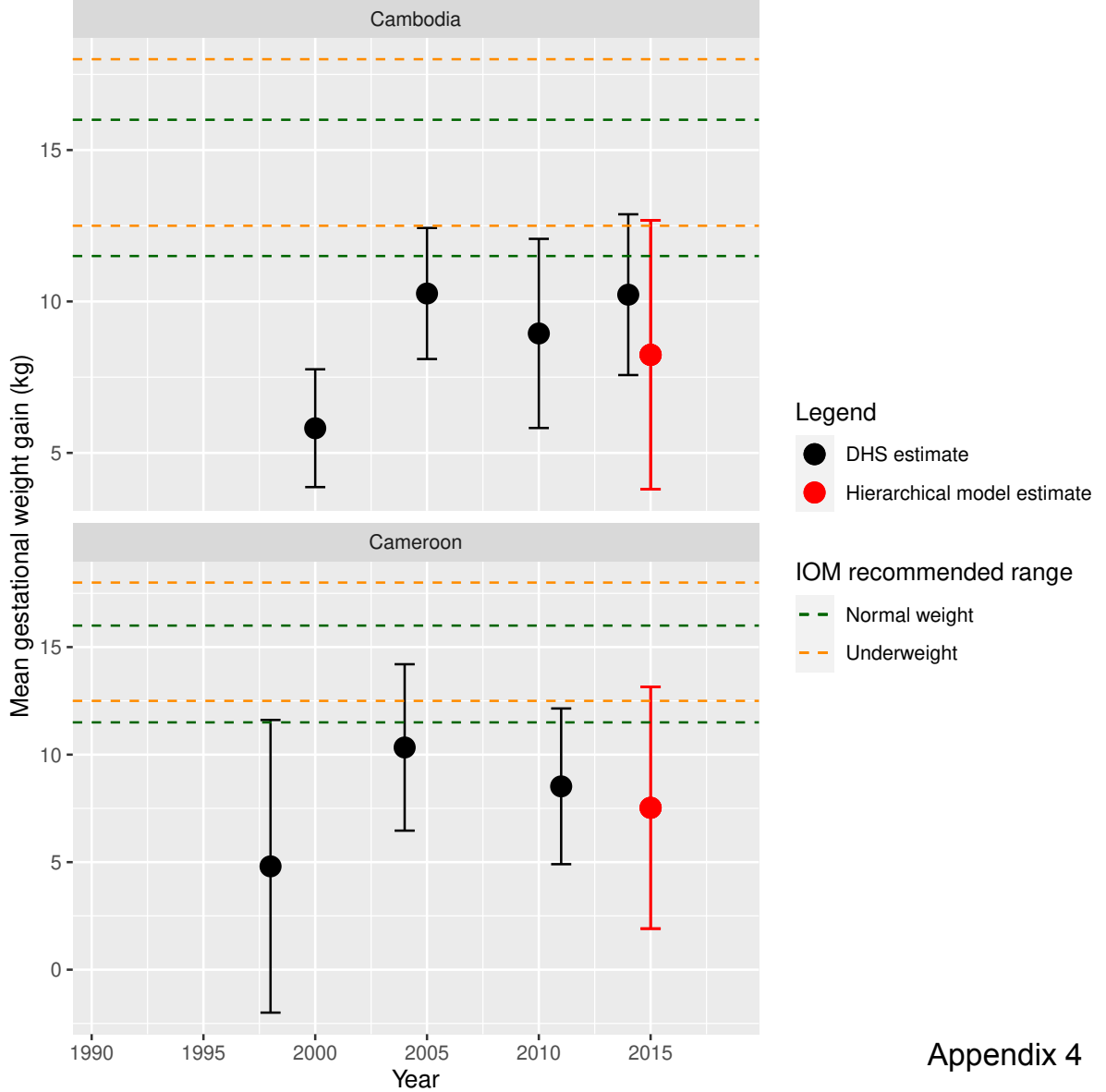

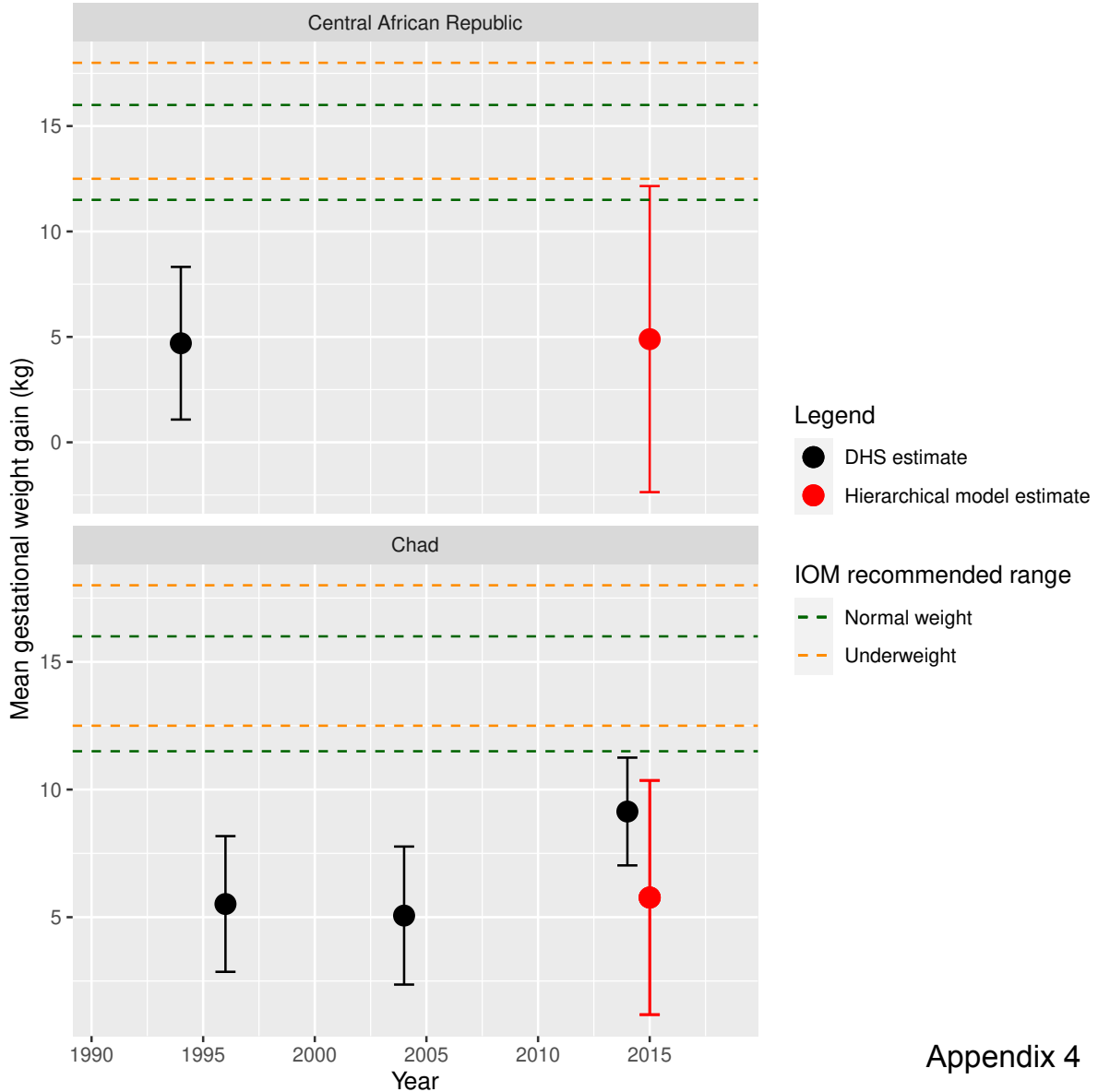

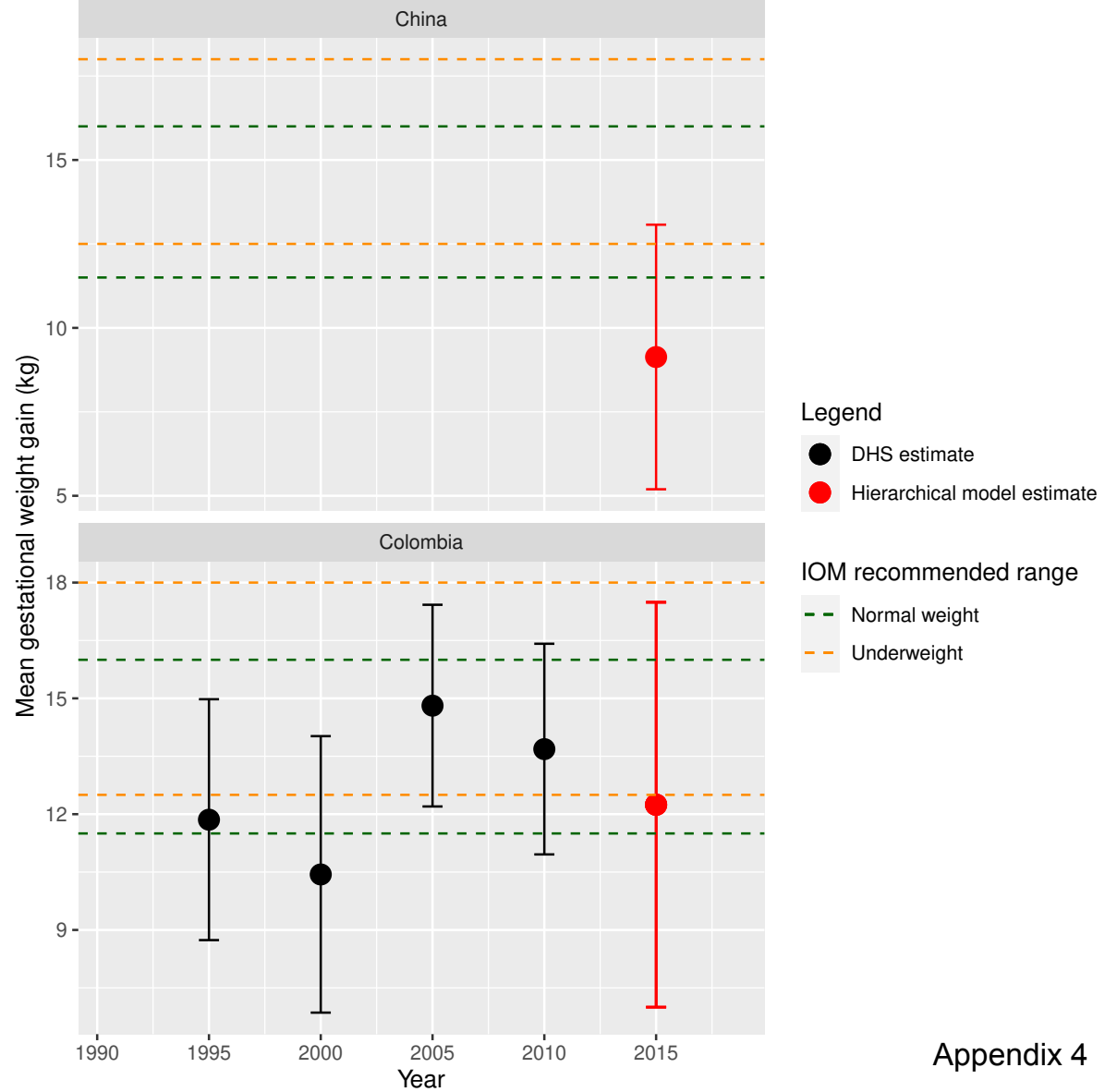

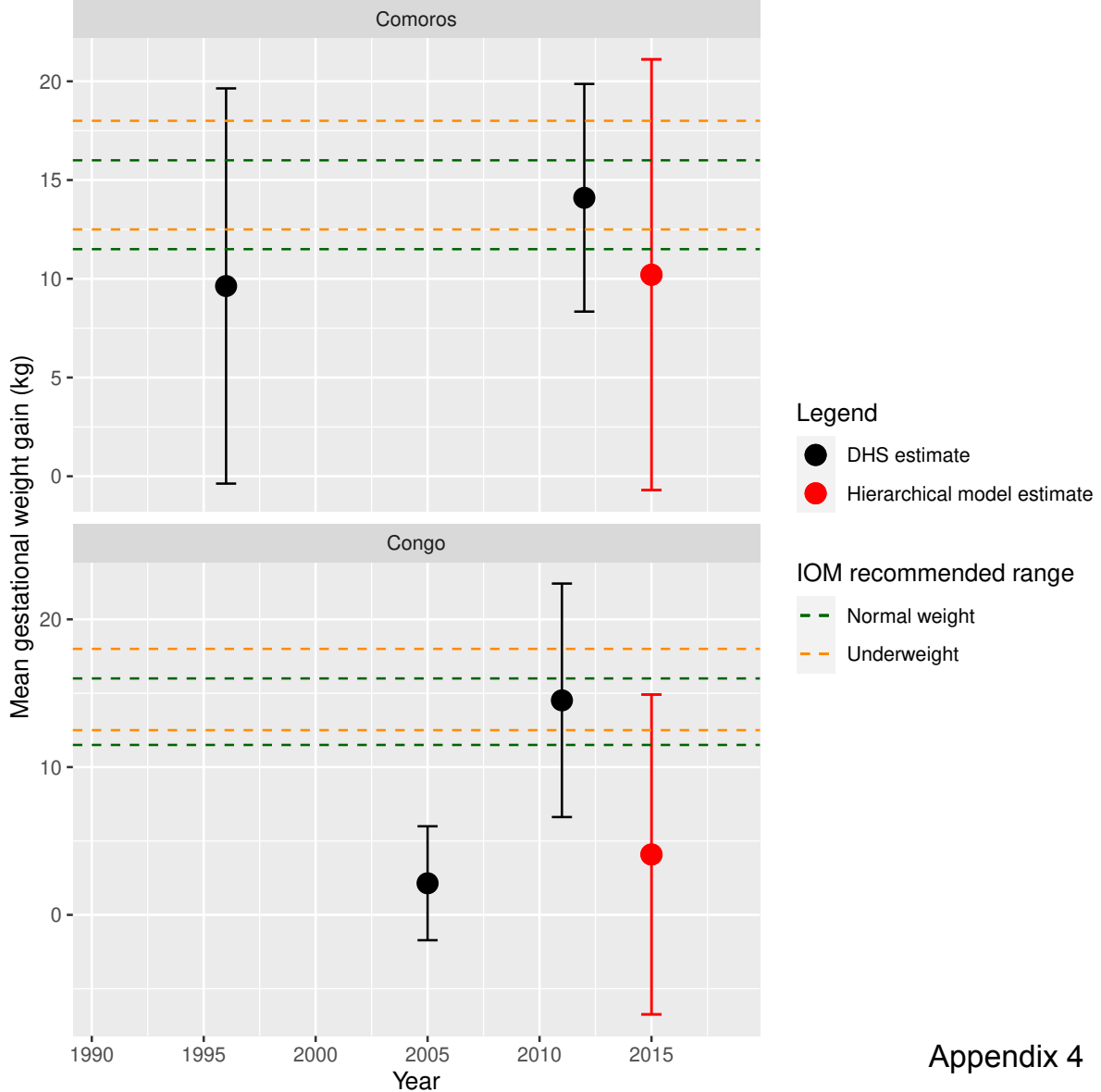

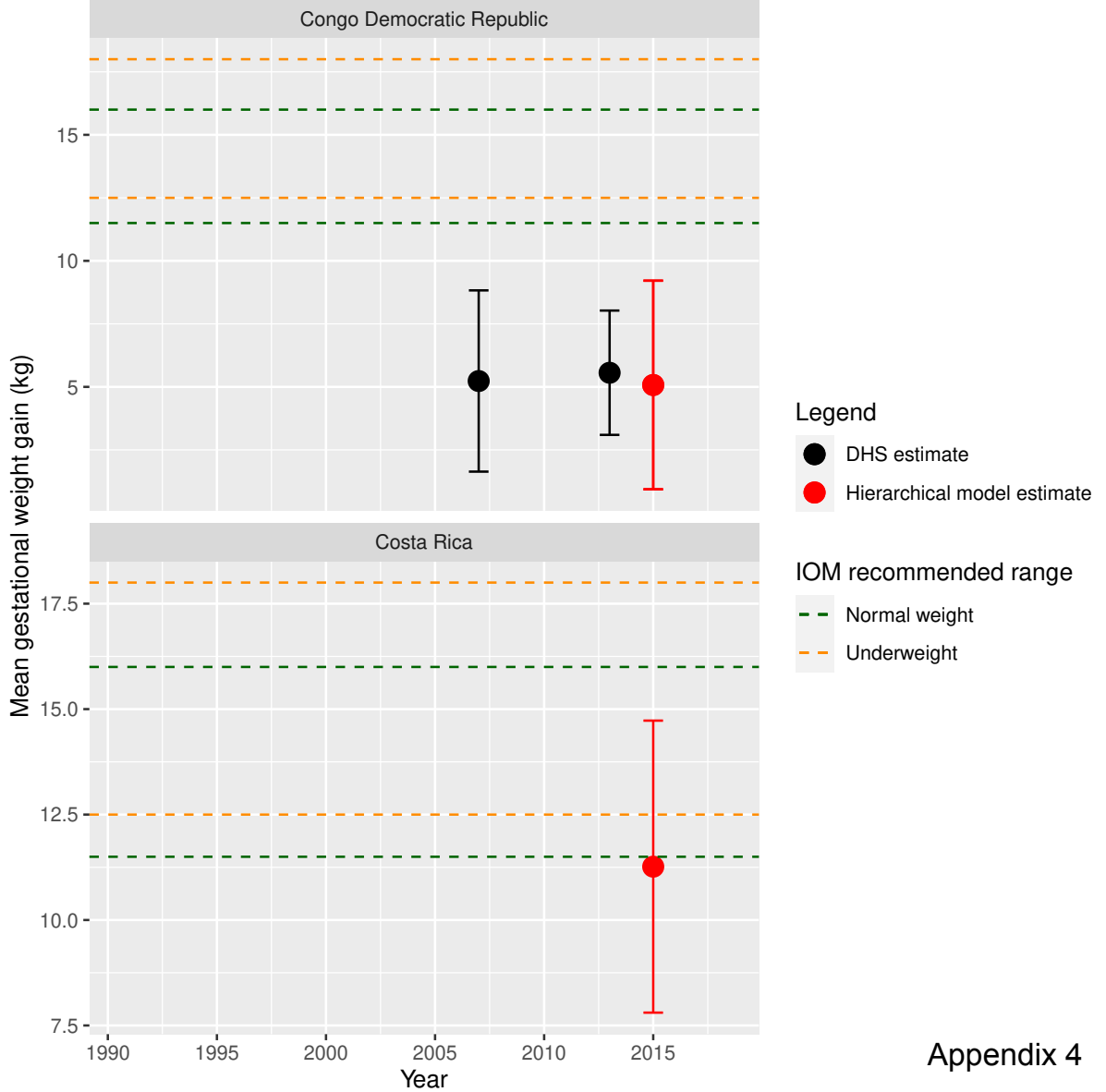

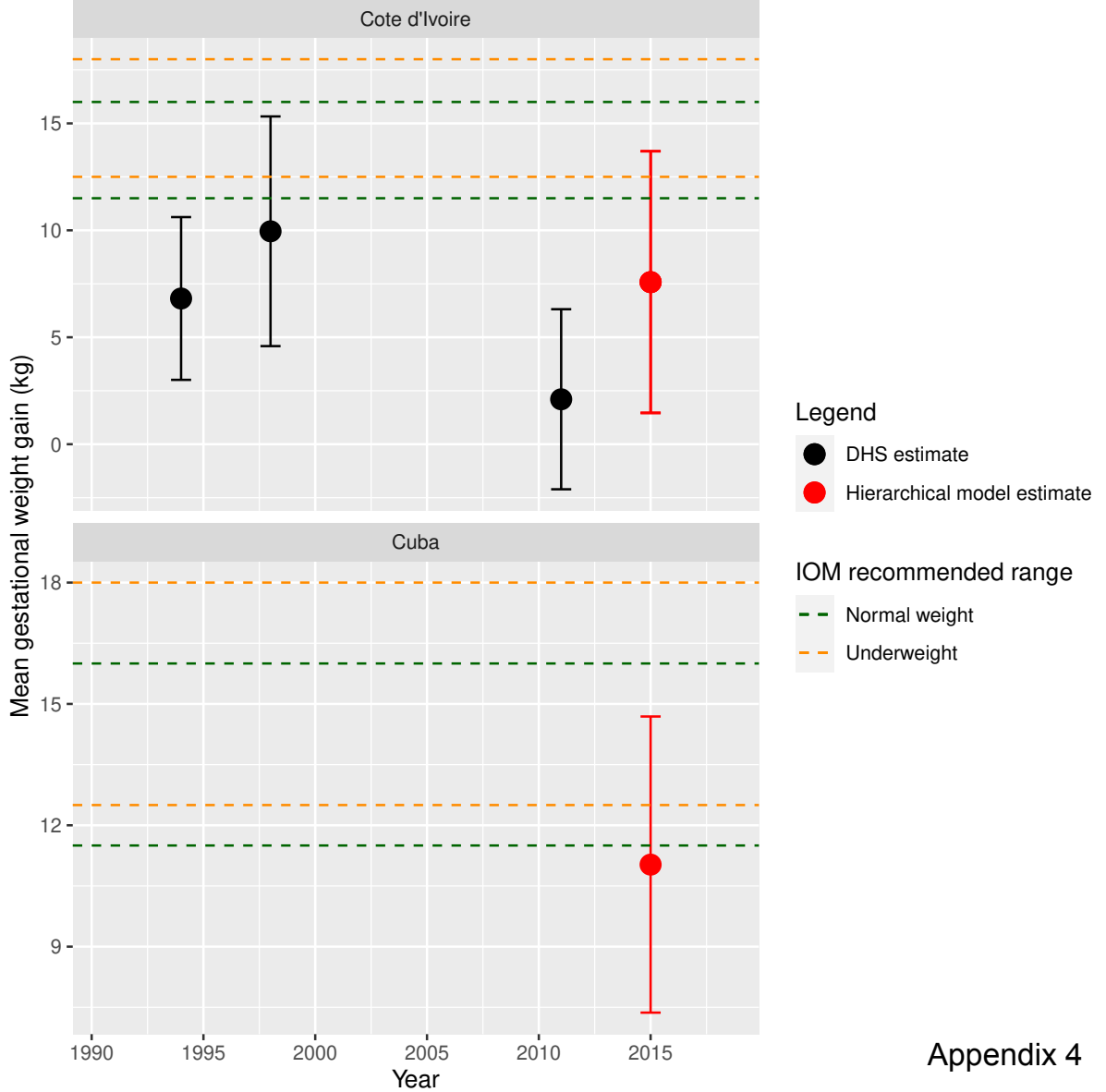

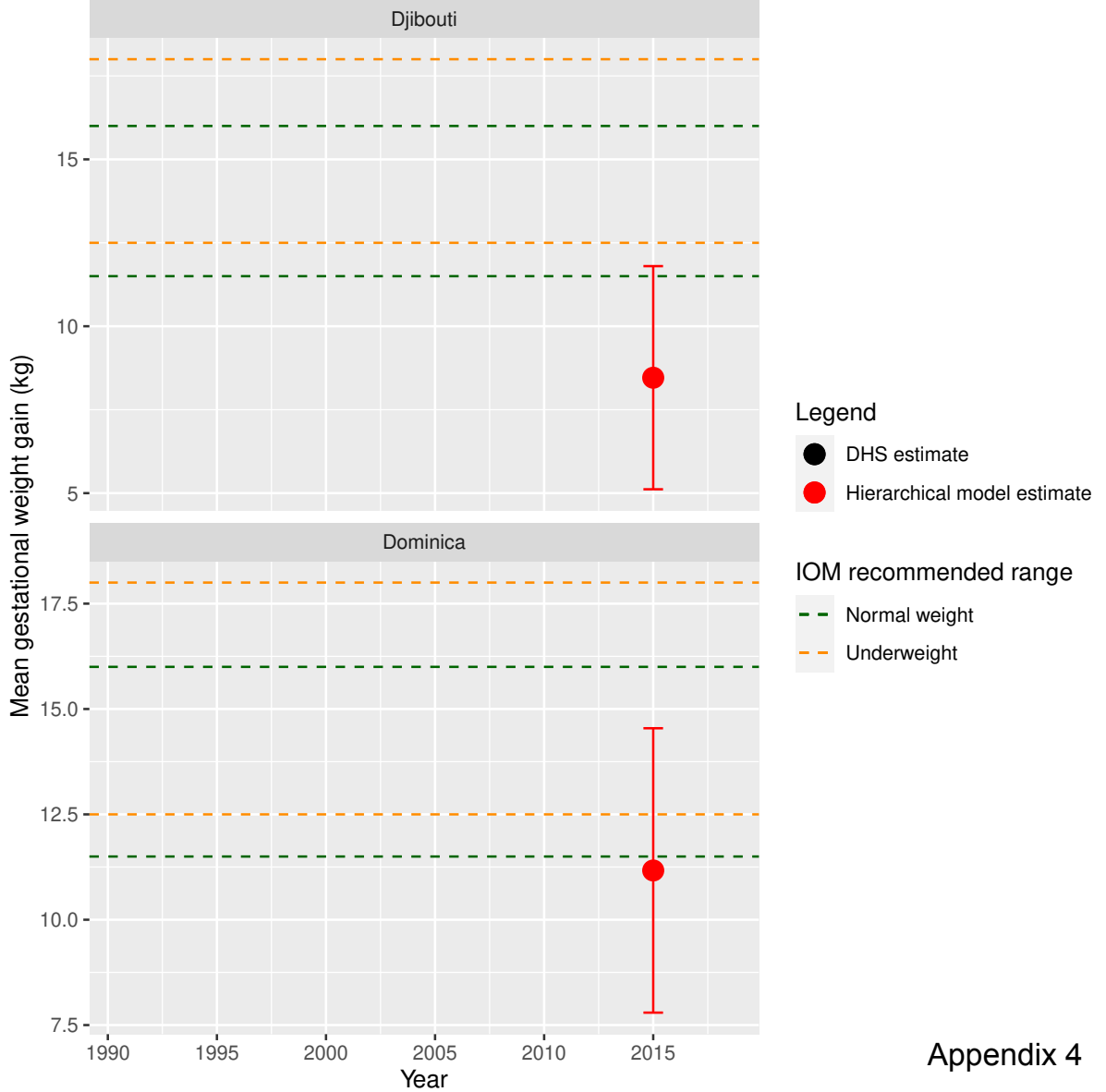

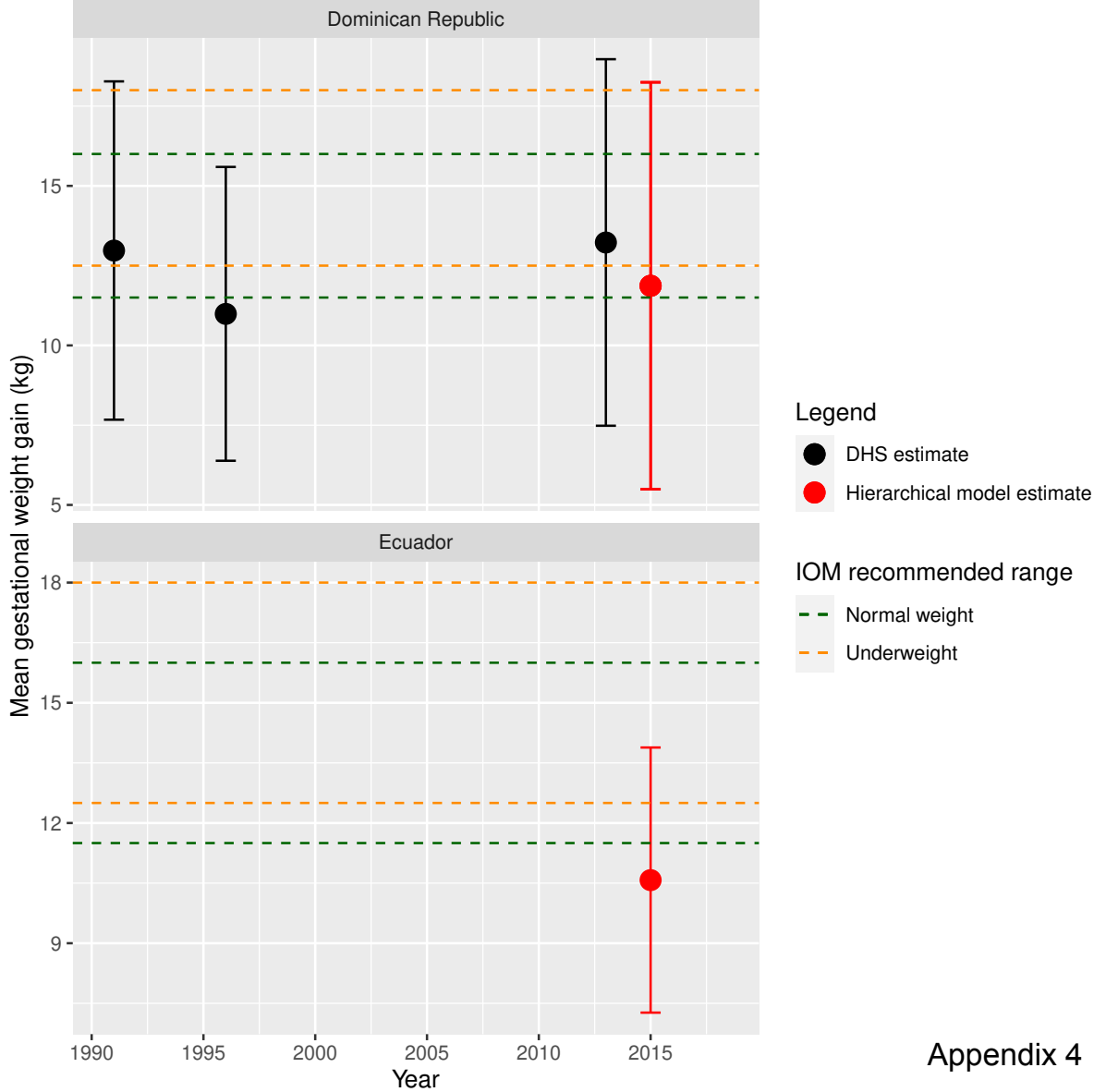

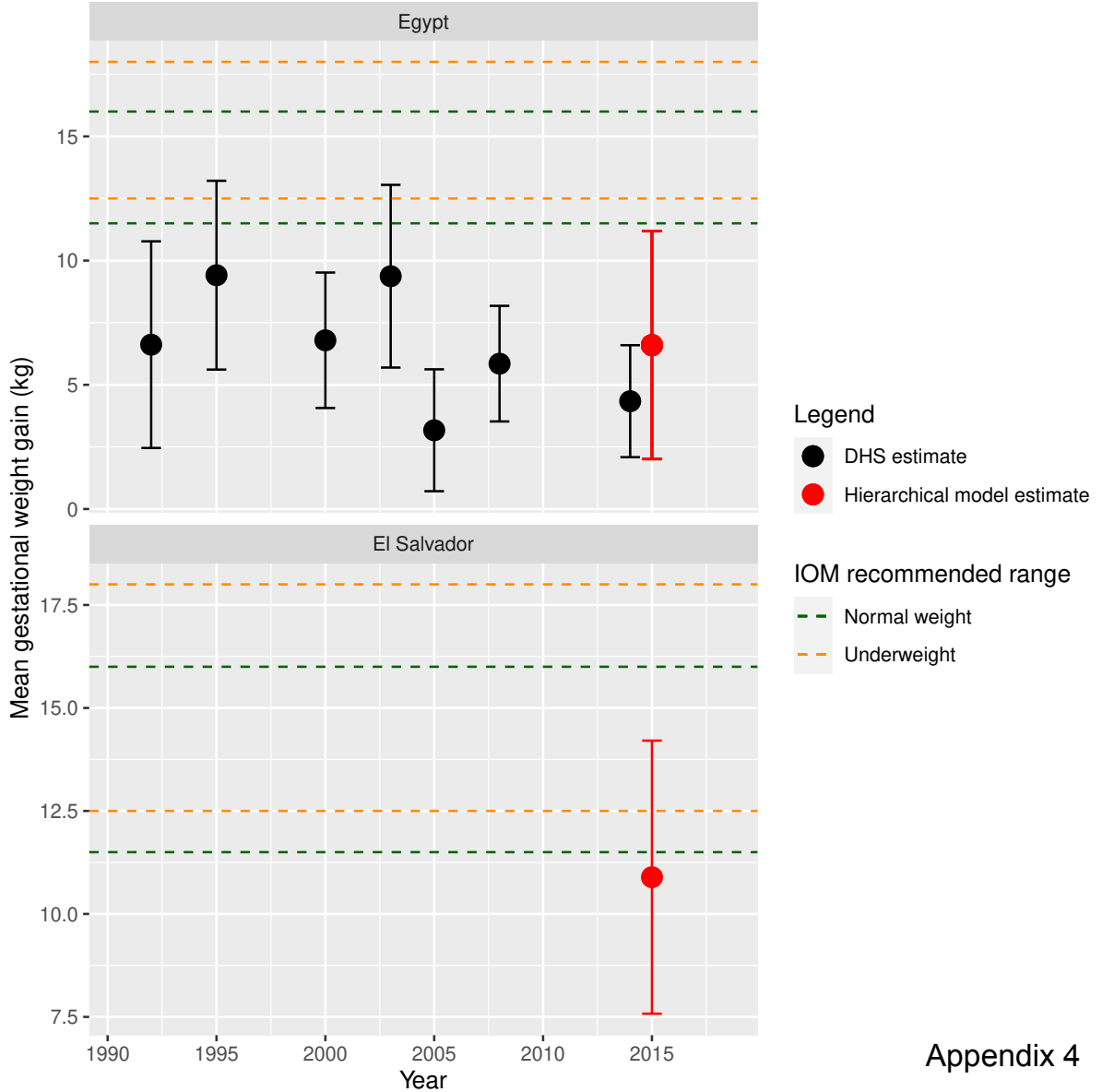

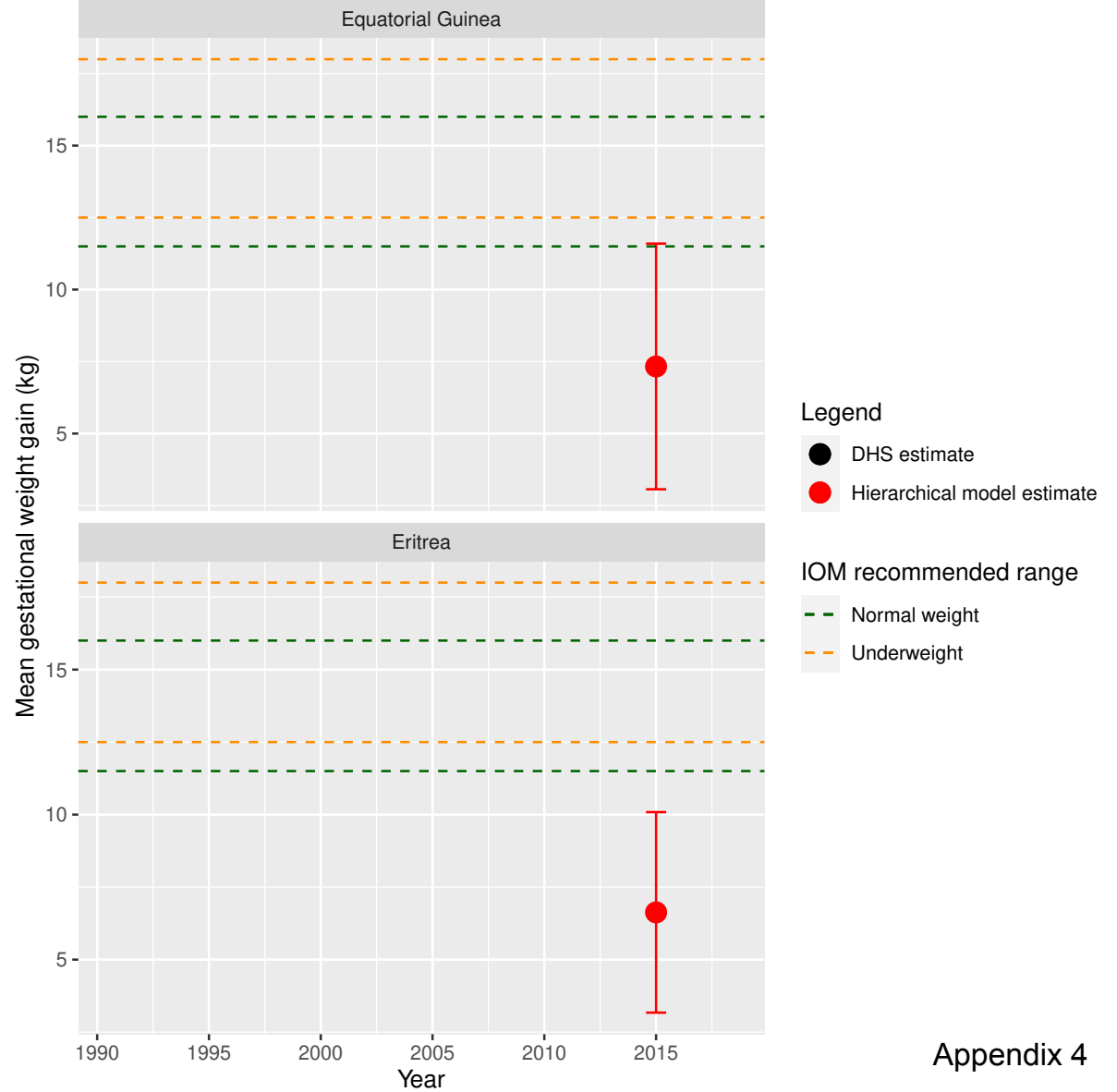

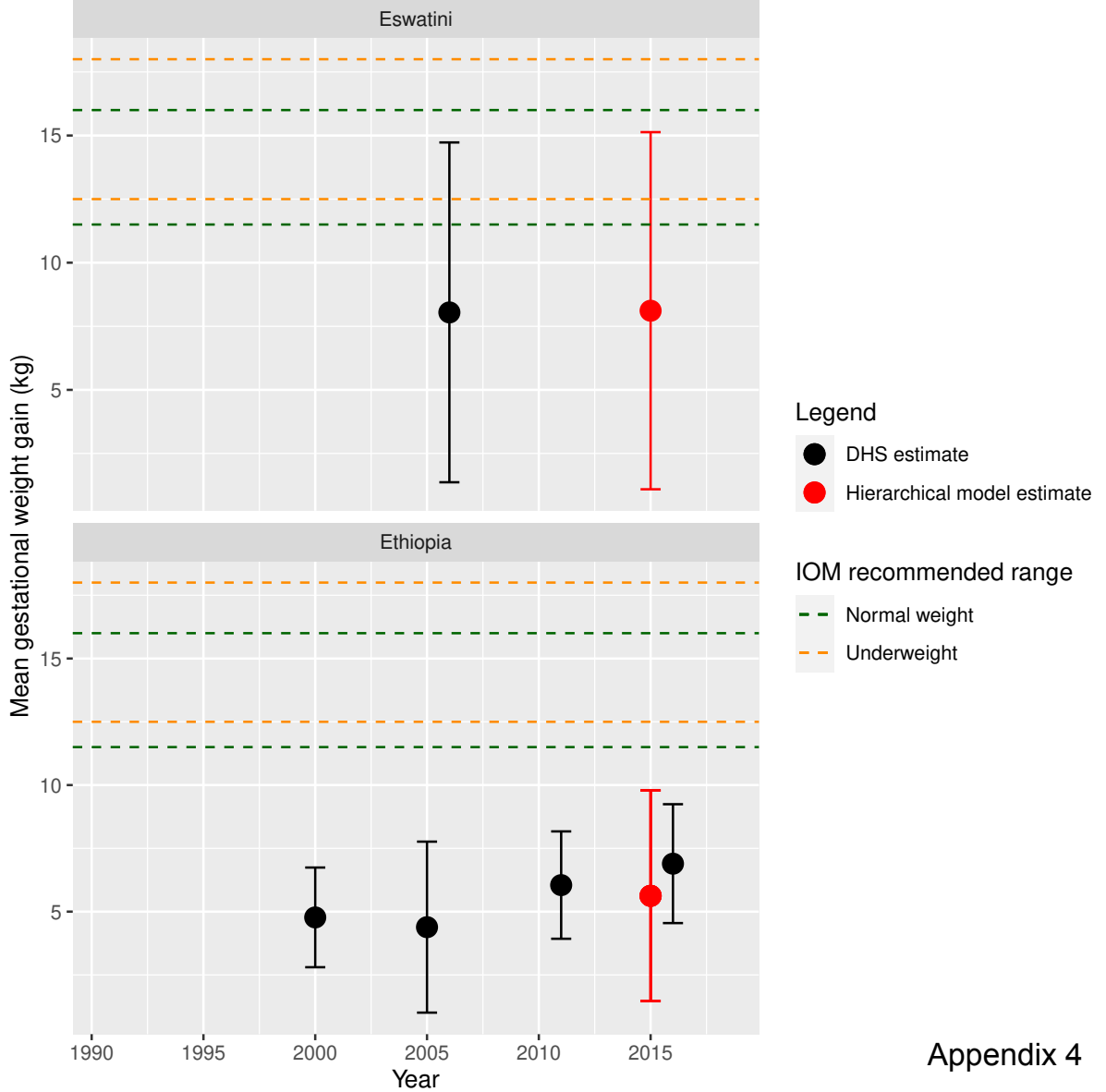

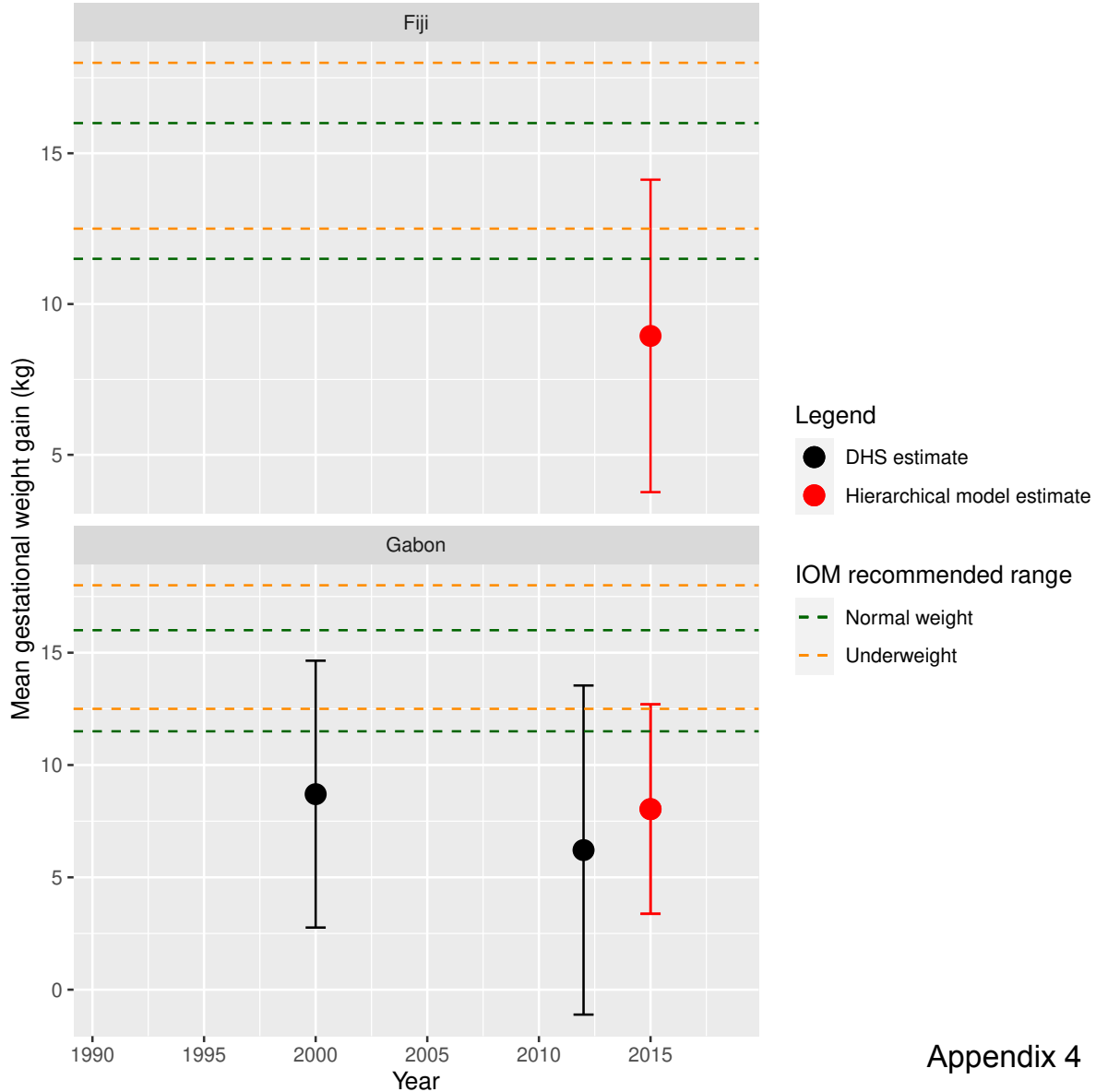

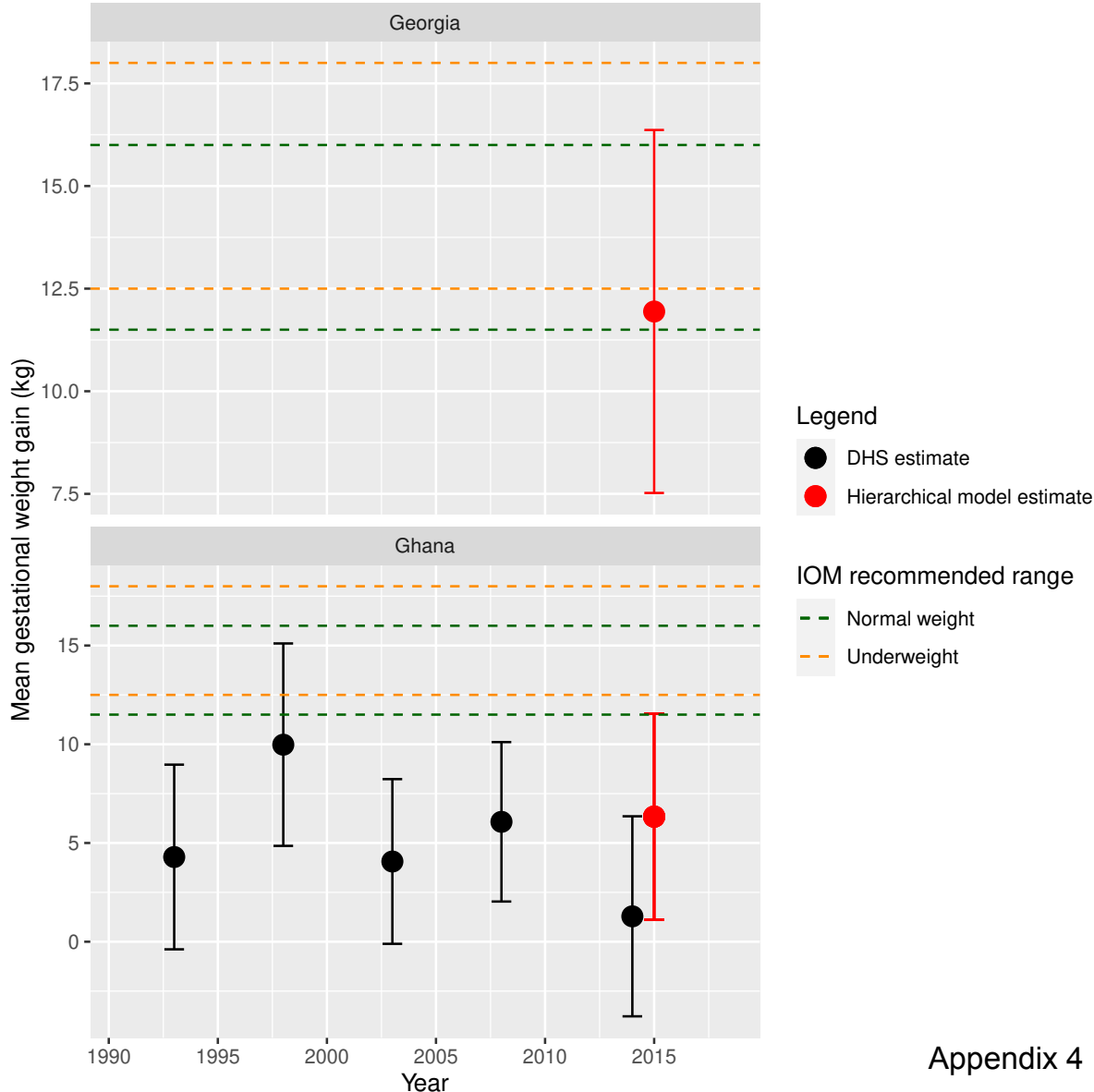

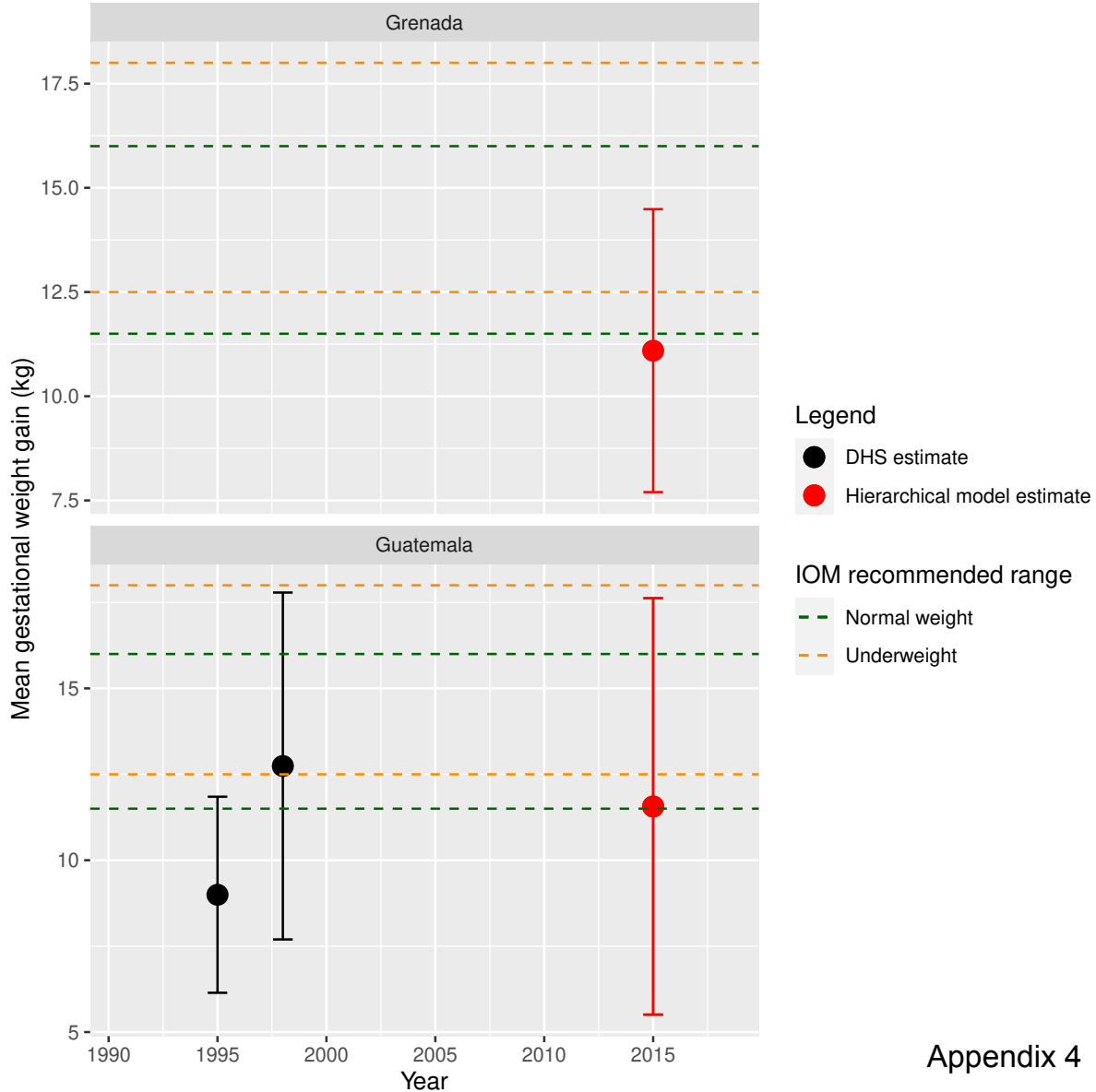

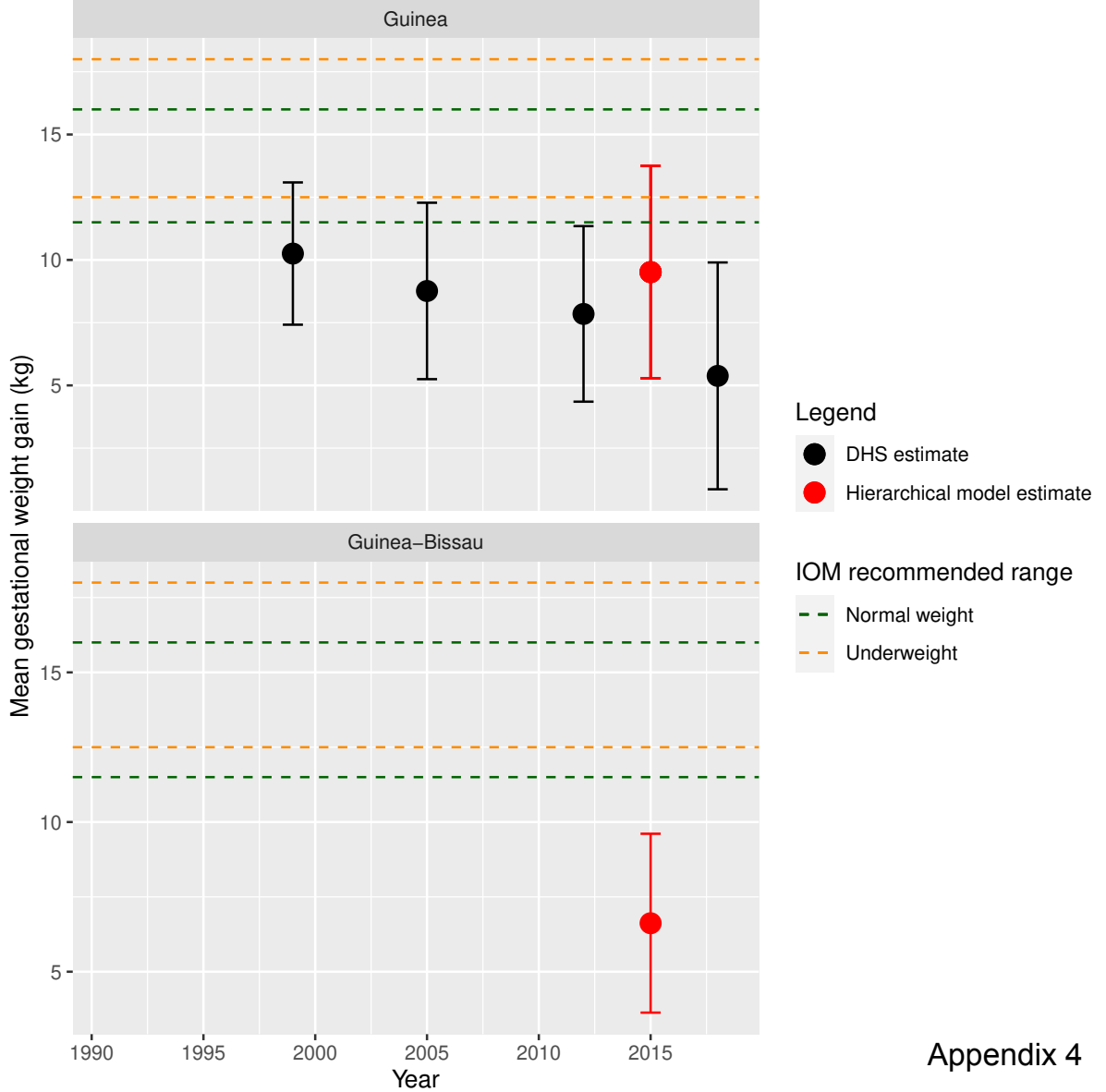

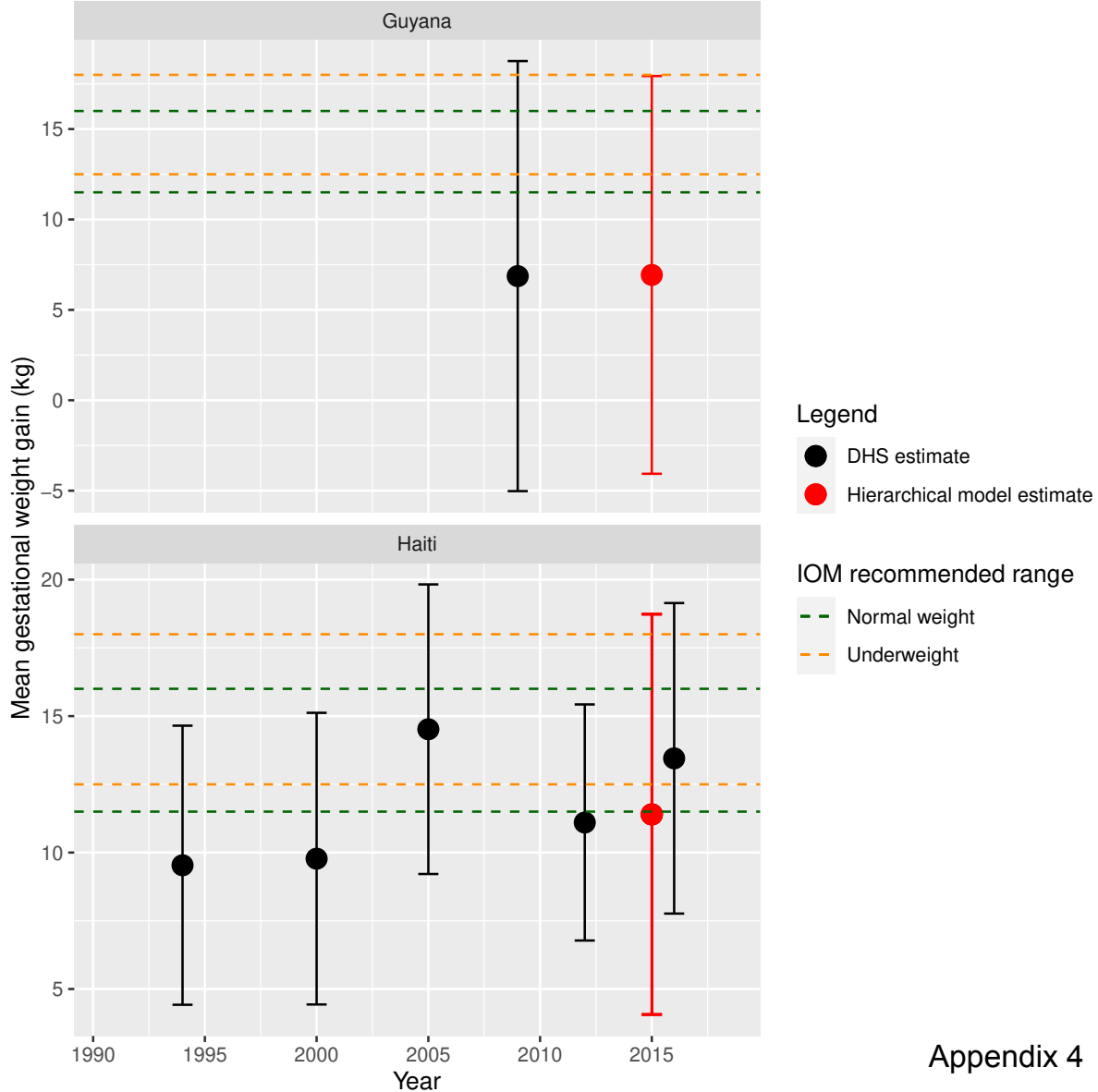

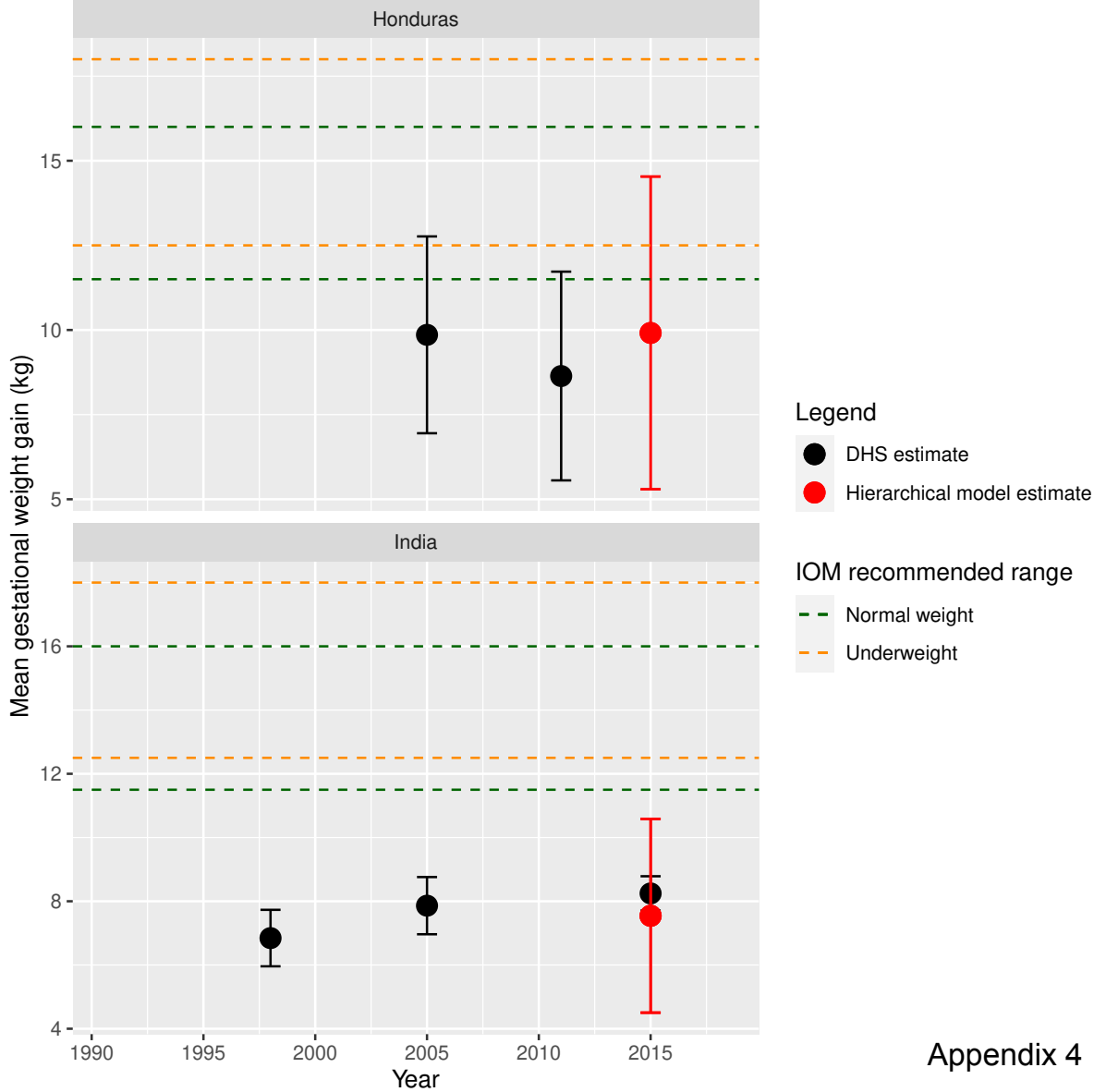

Appendix 4

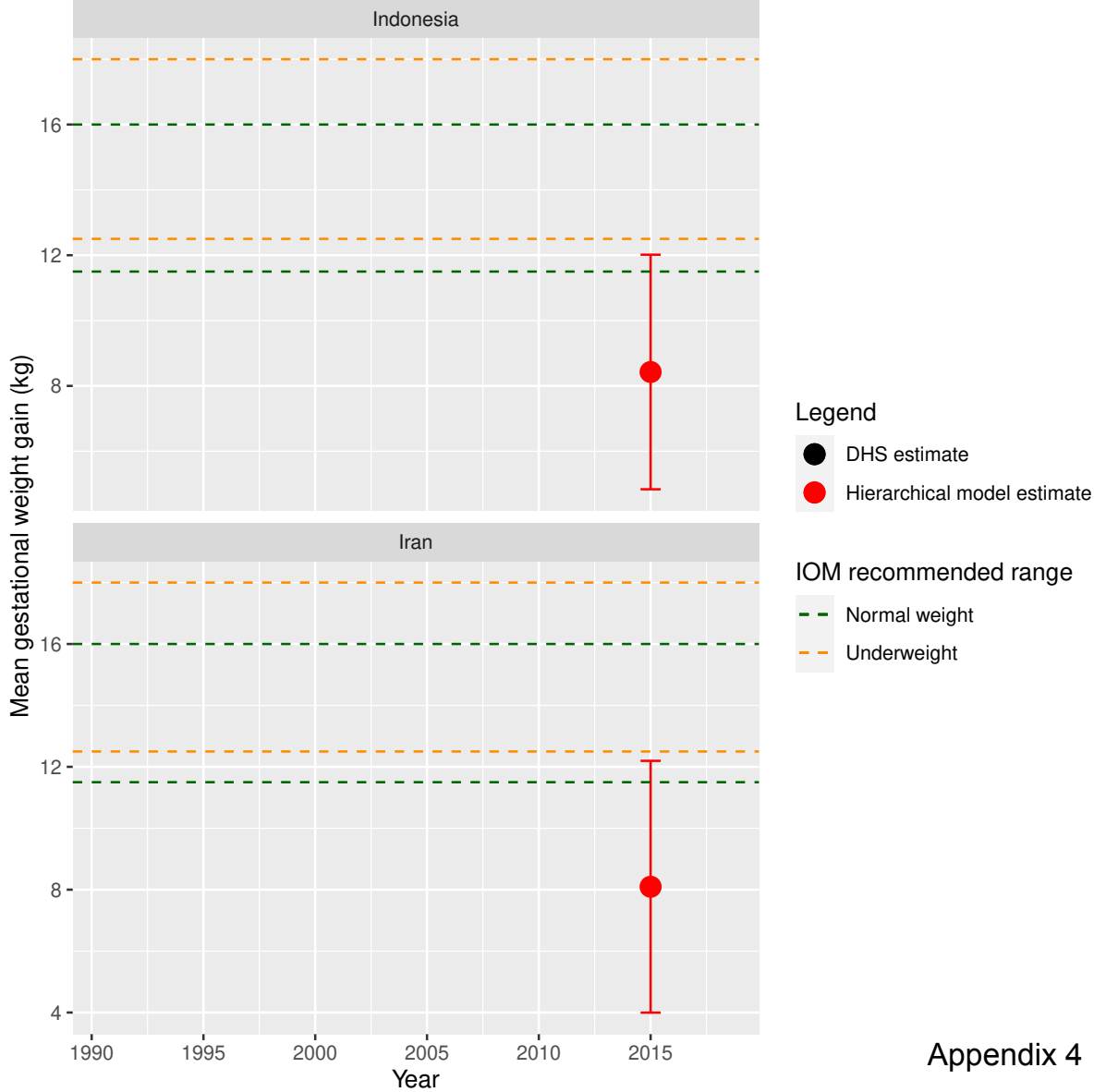

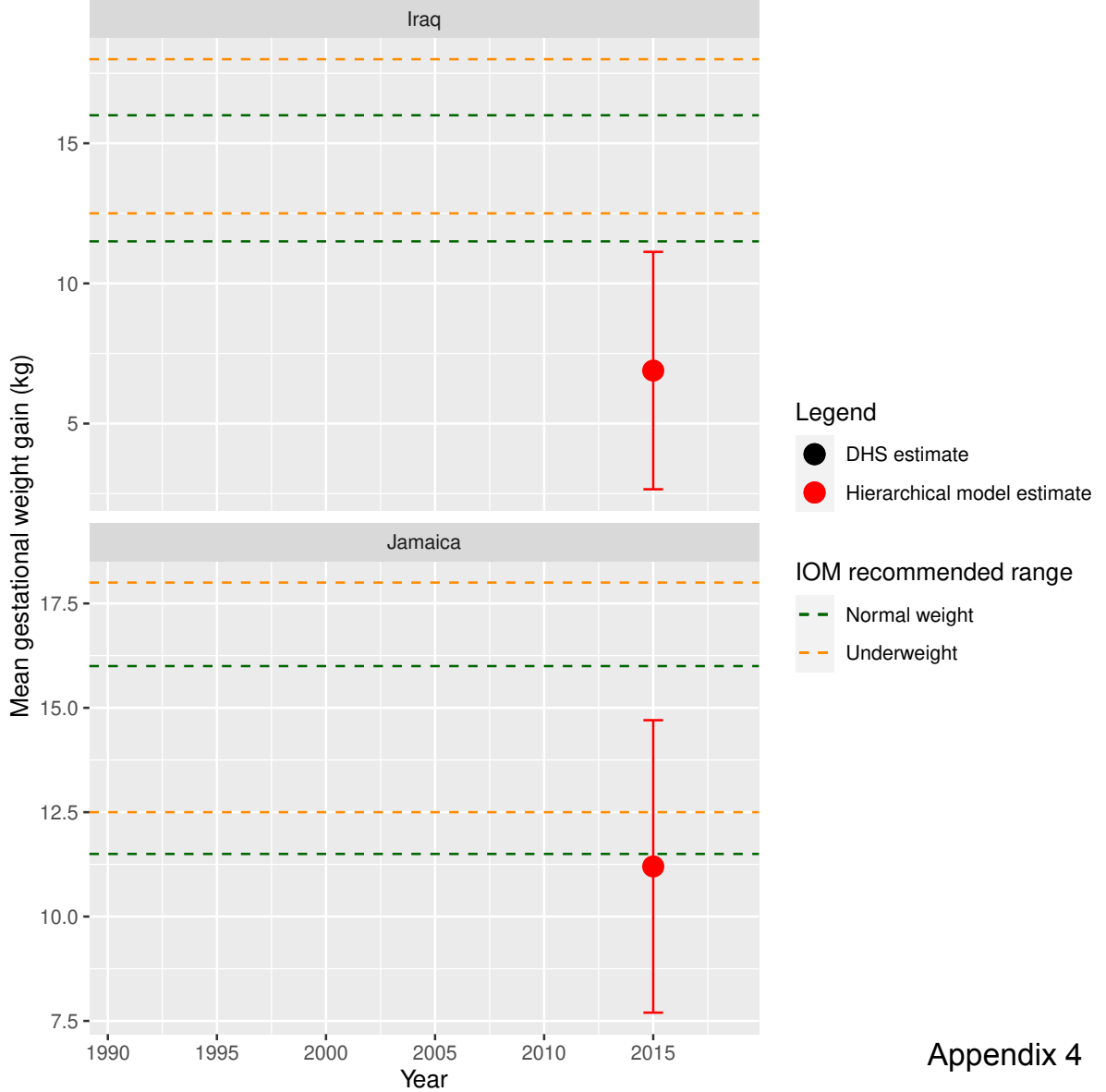

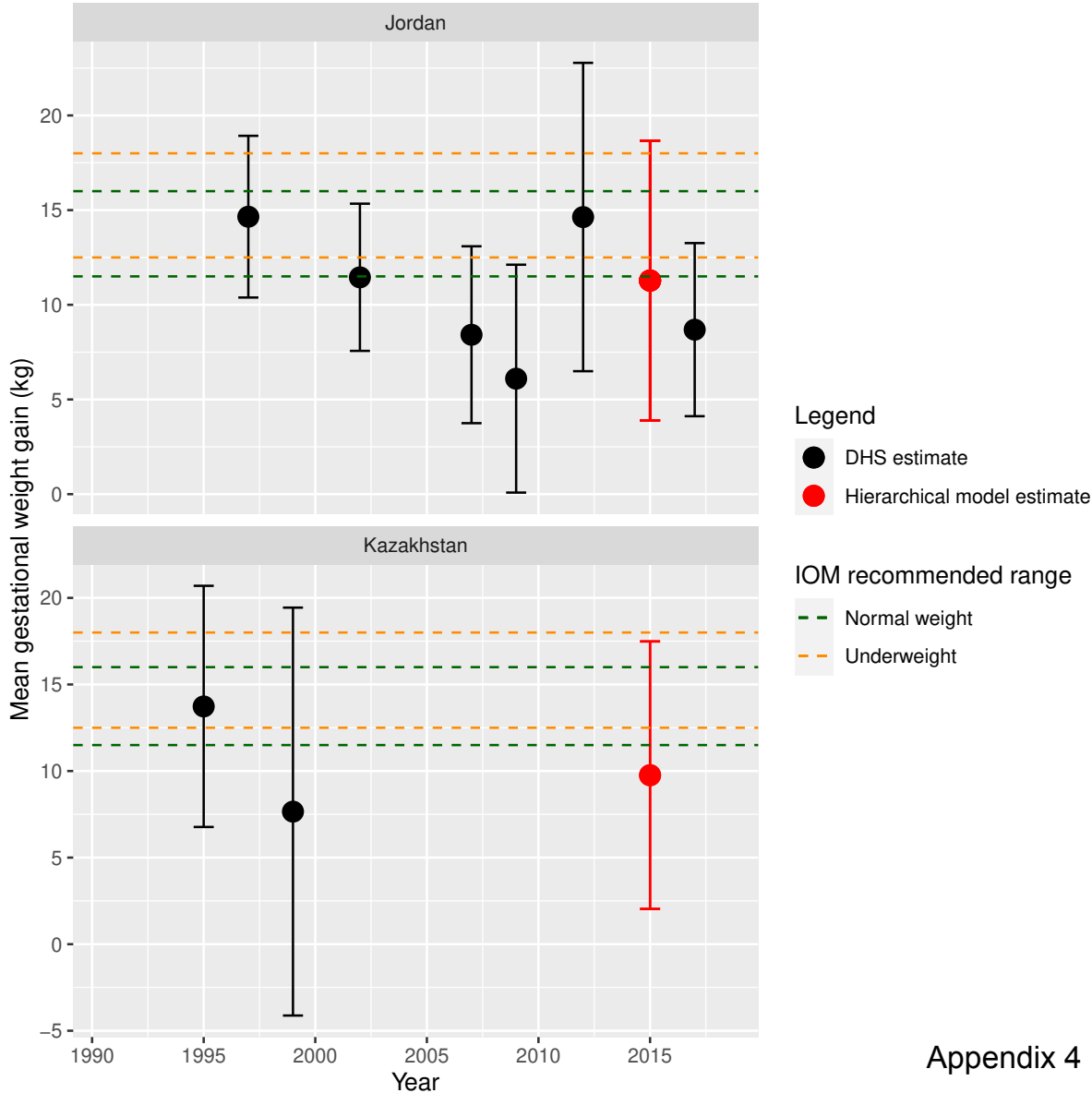

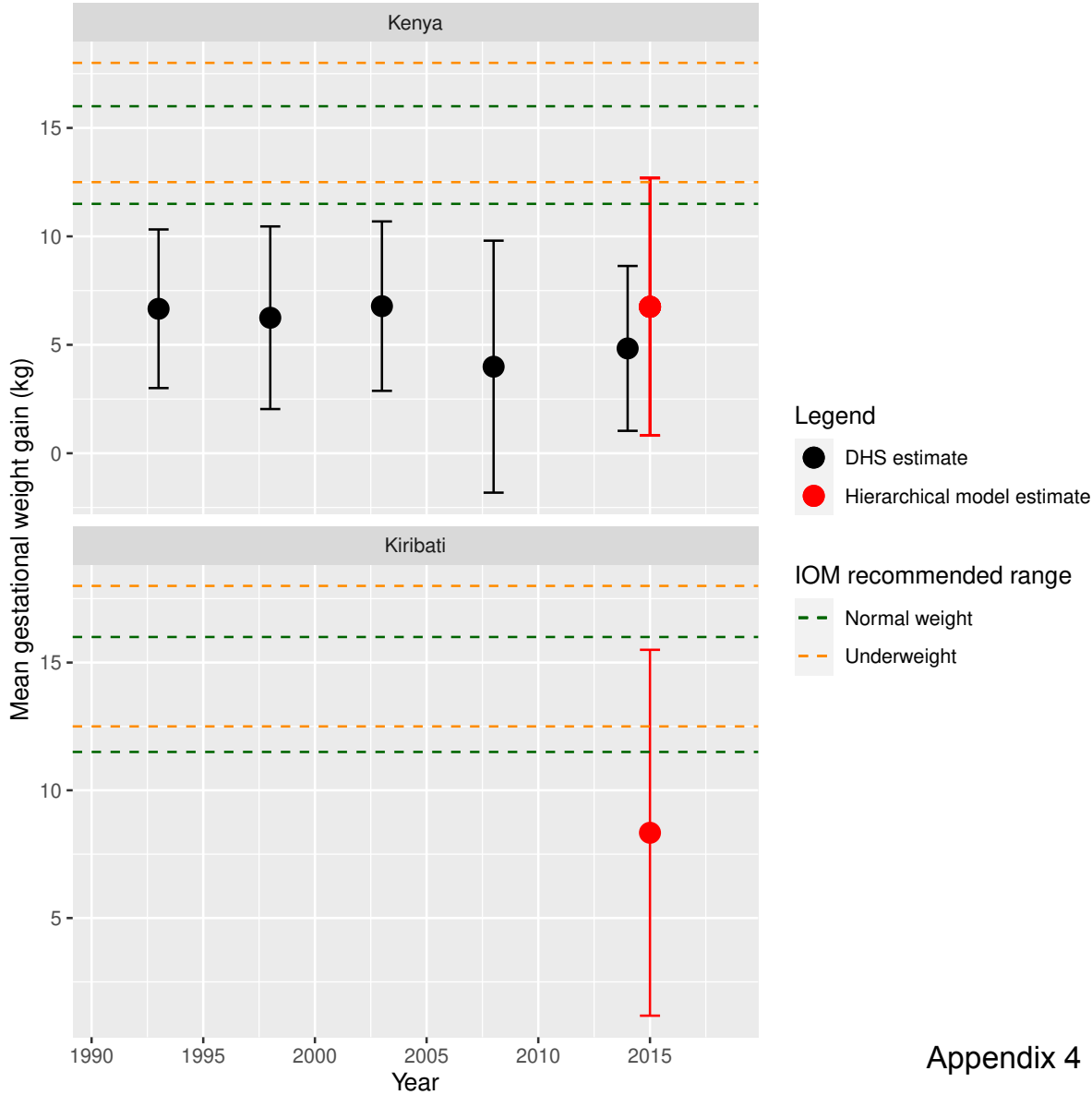

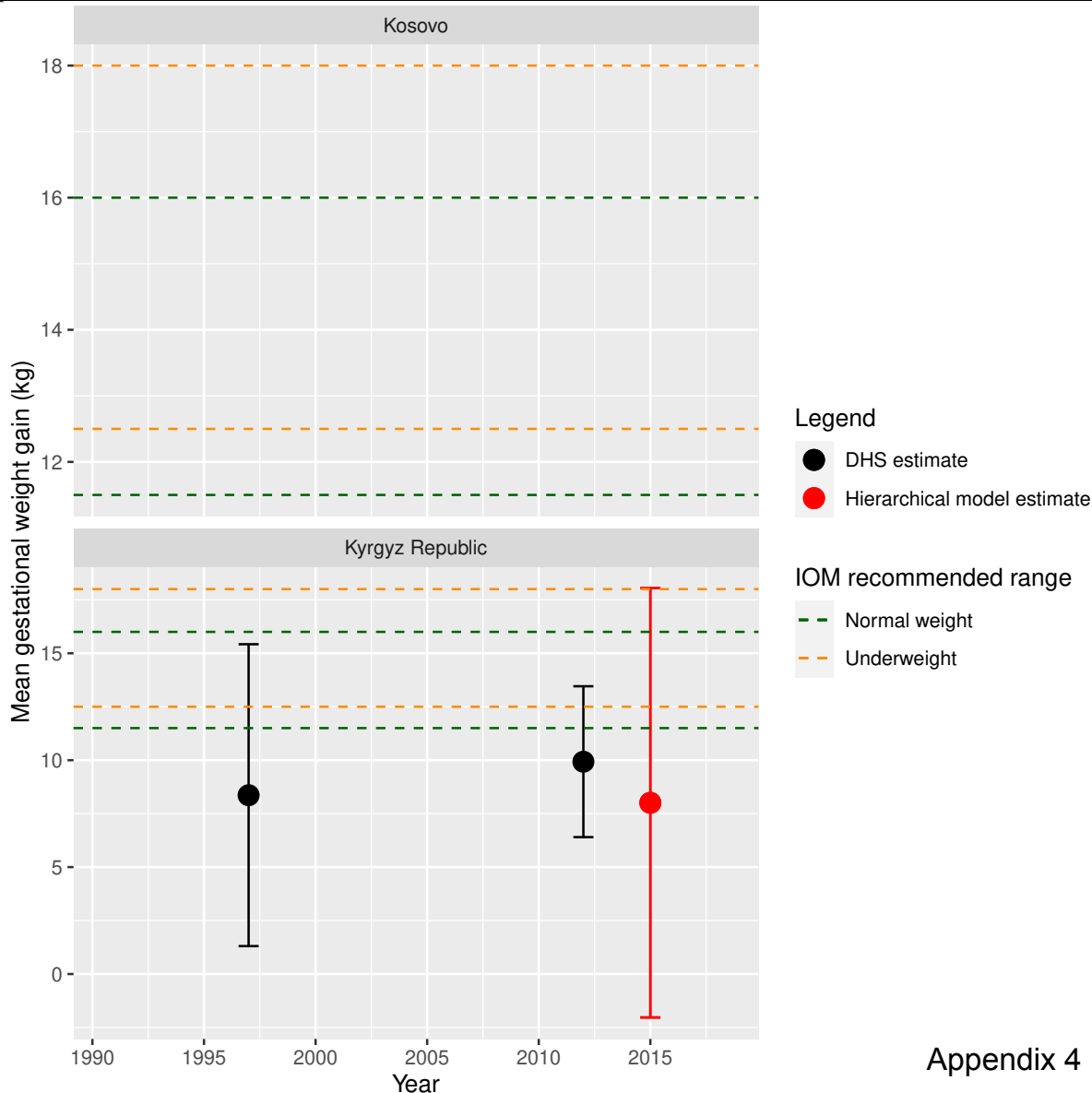

Appendix 4

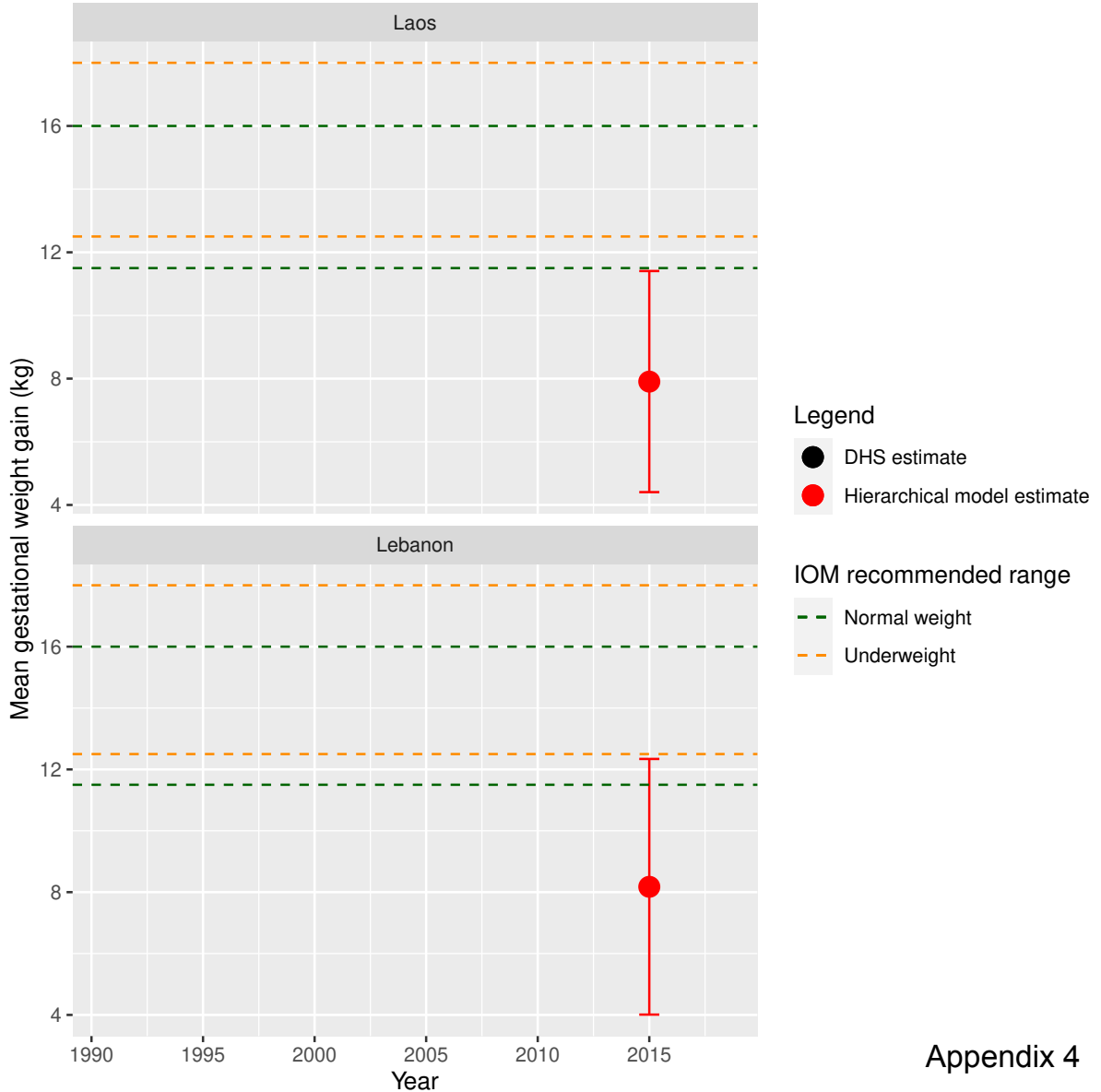

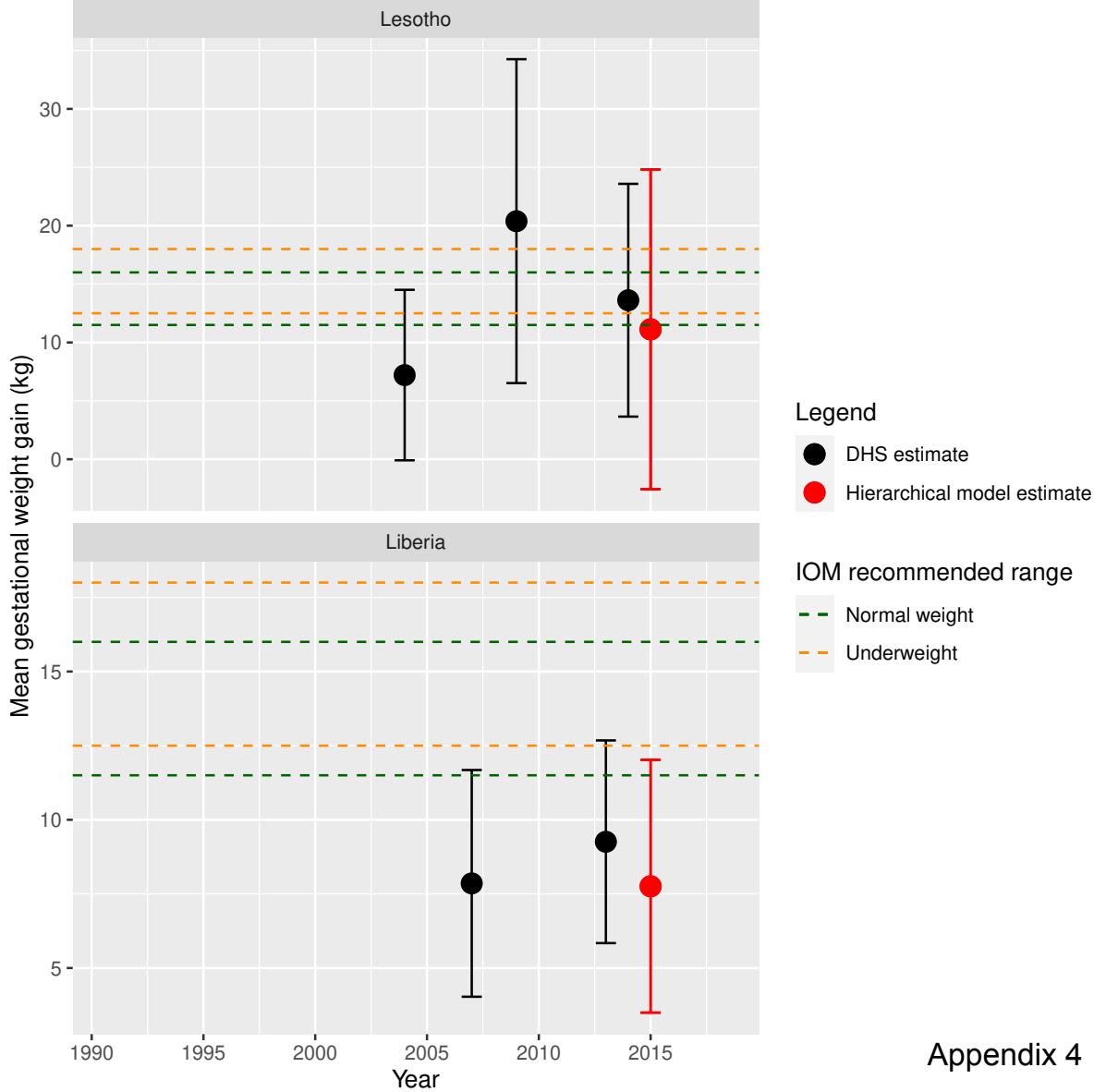

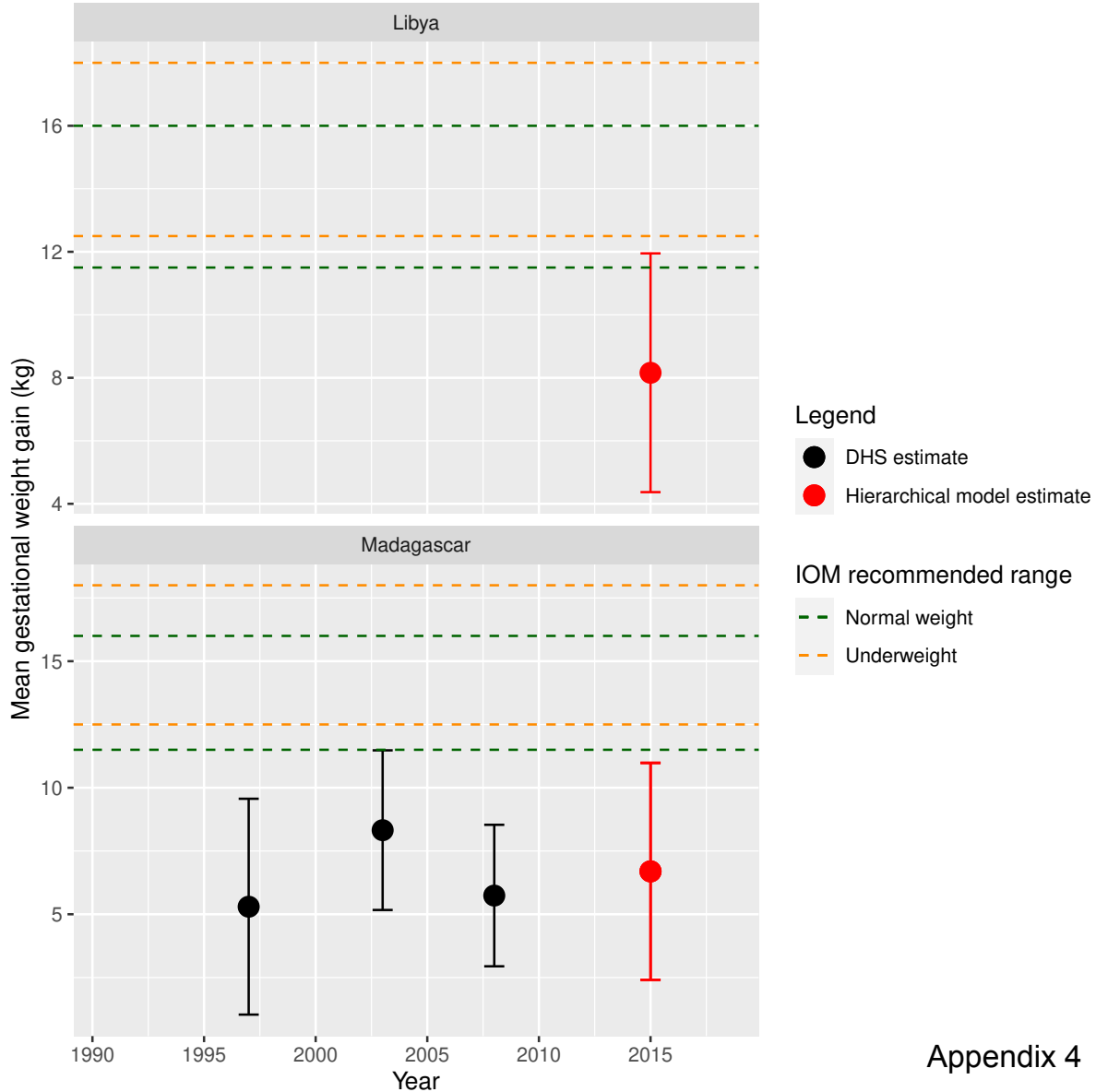

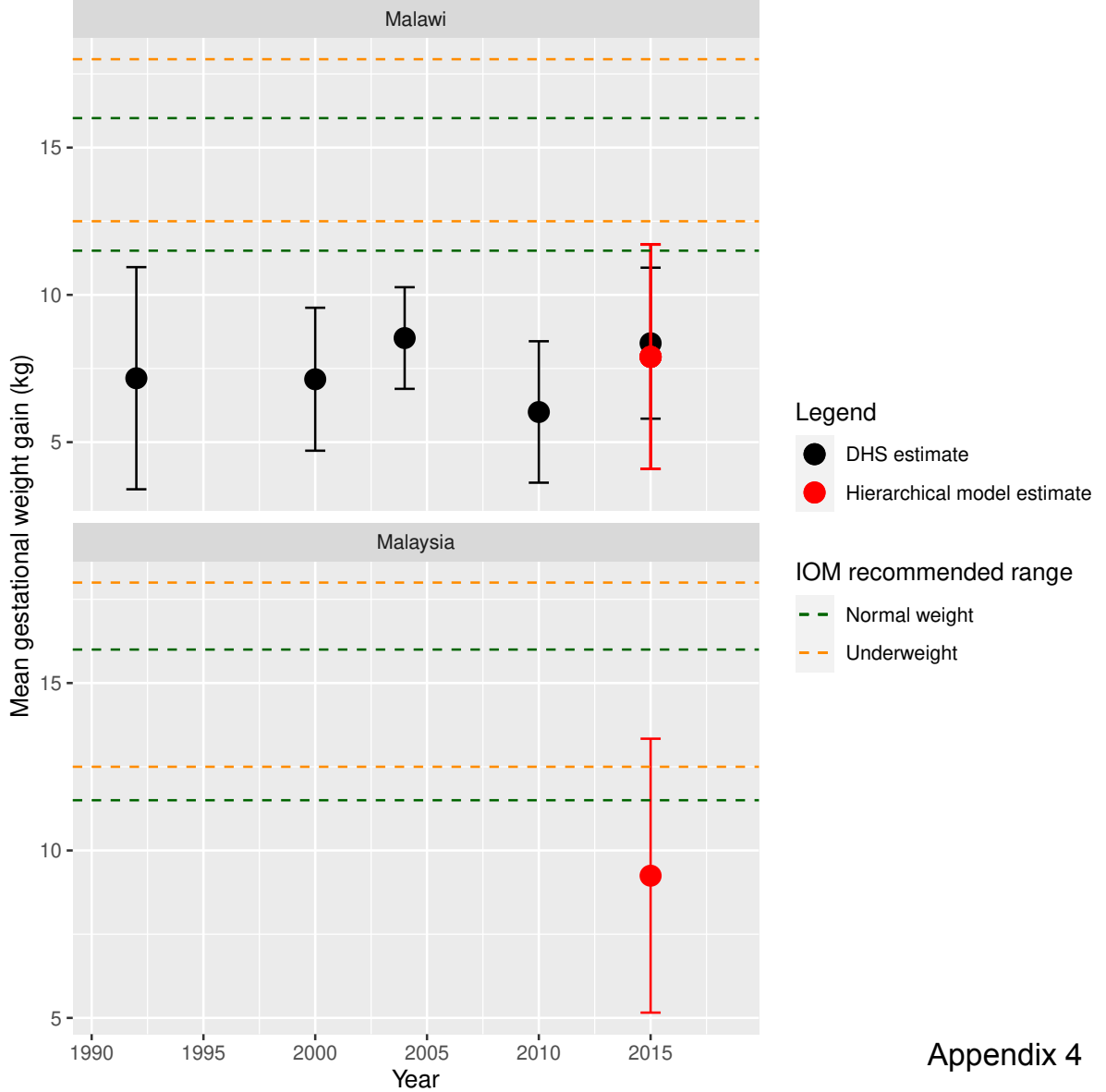

Appendix 4

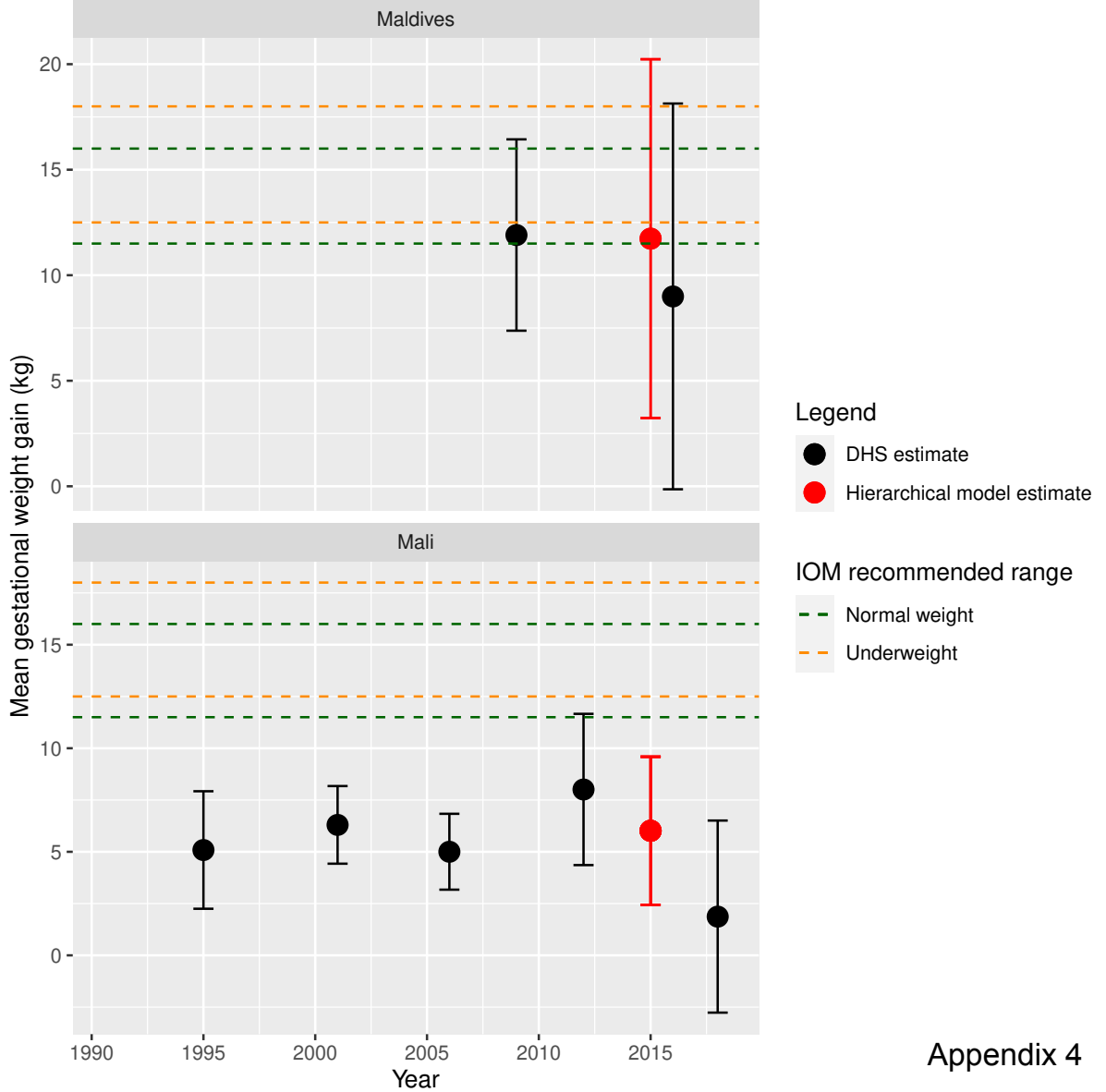

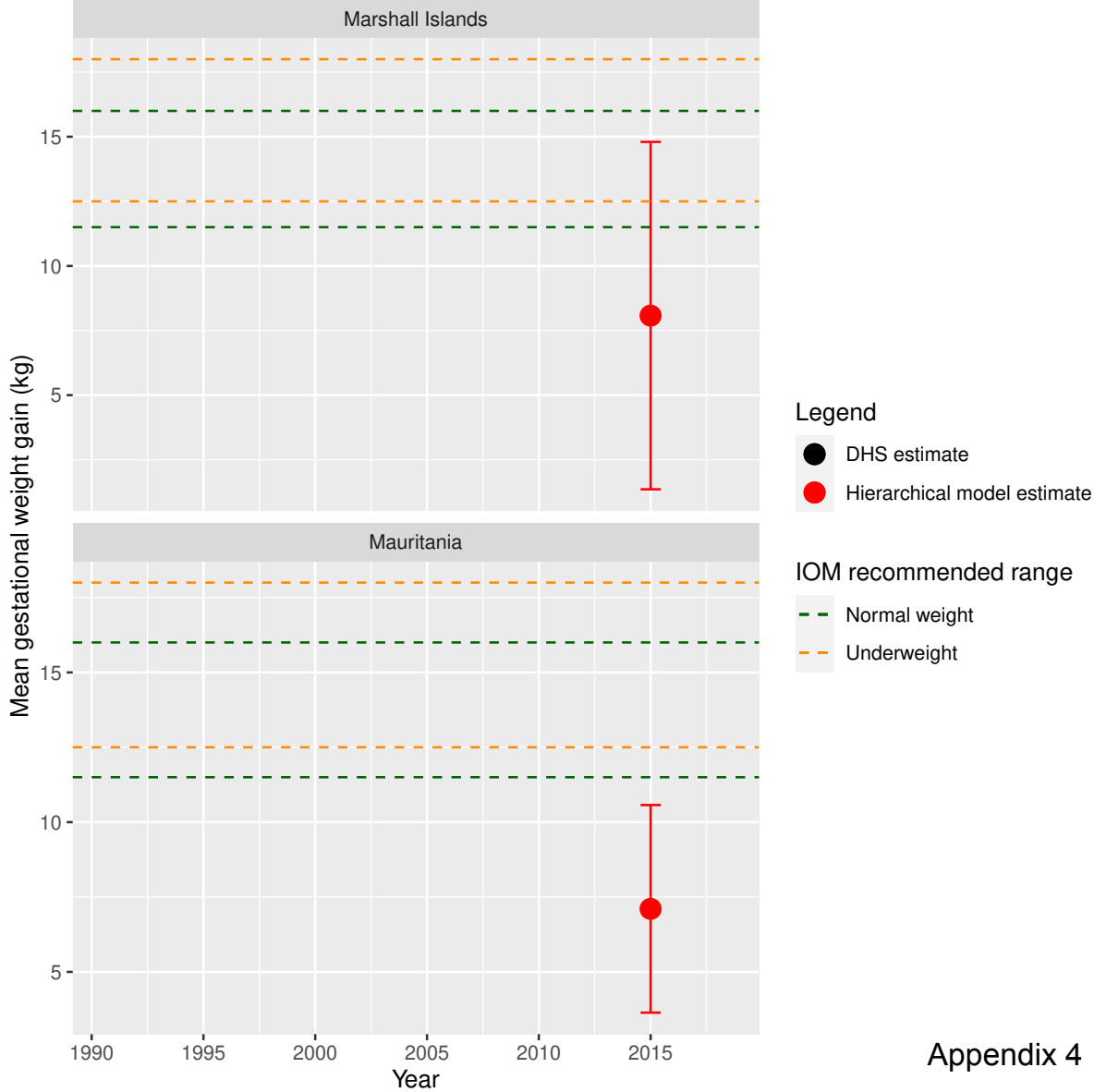

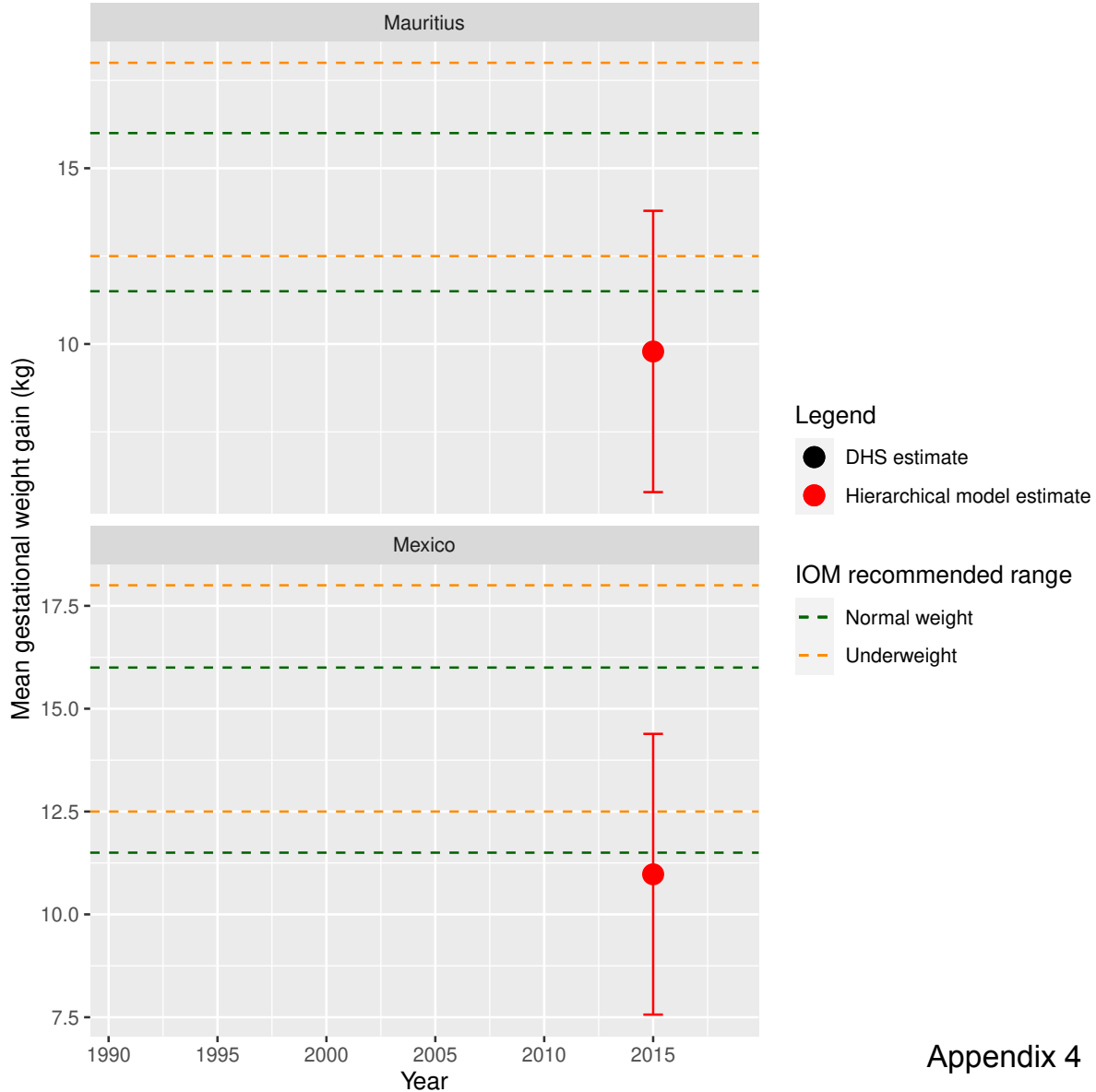

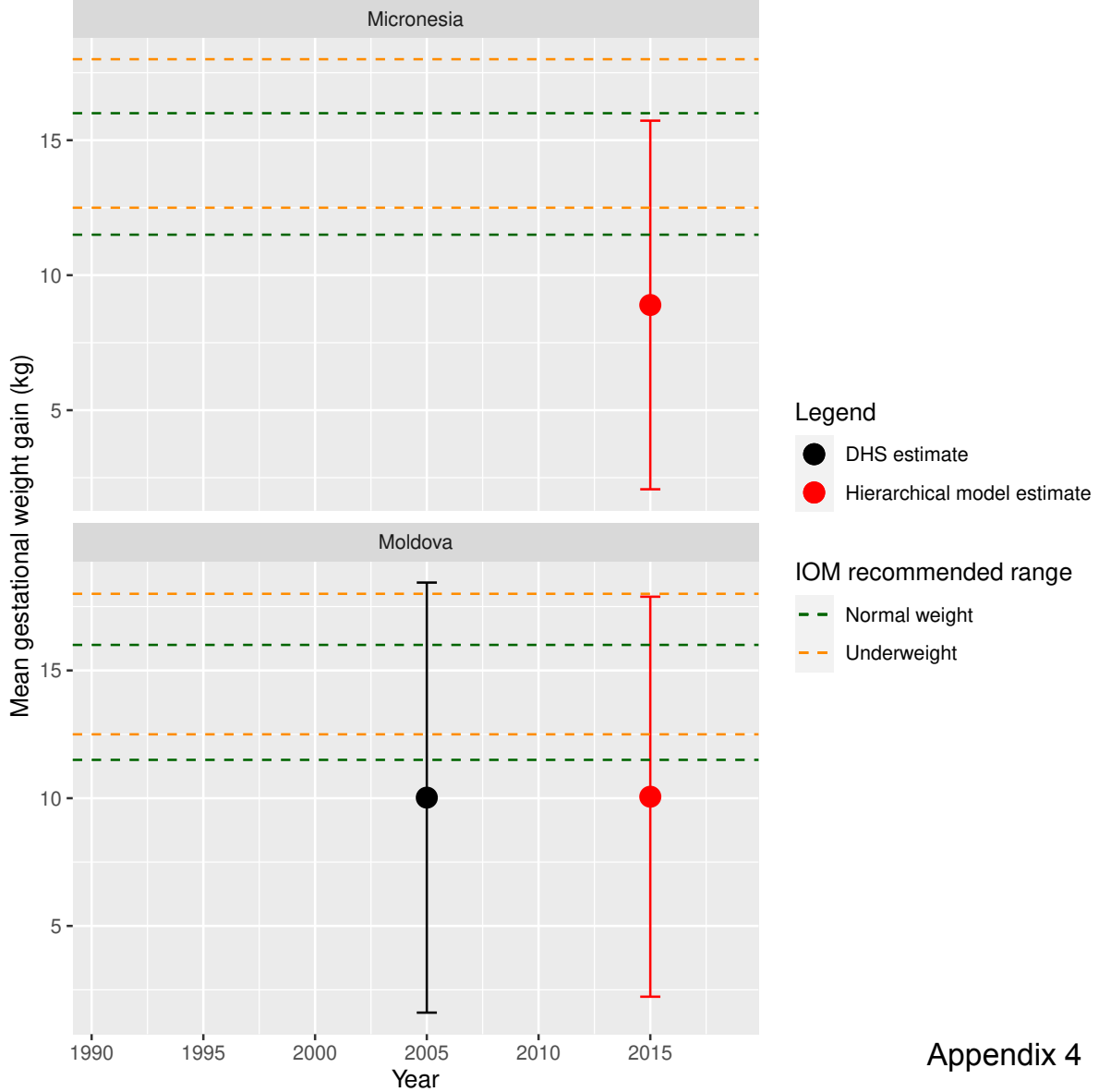

Appendix 4

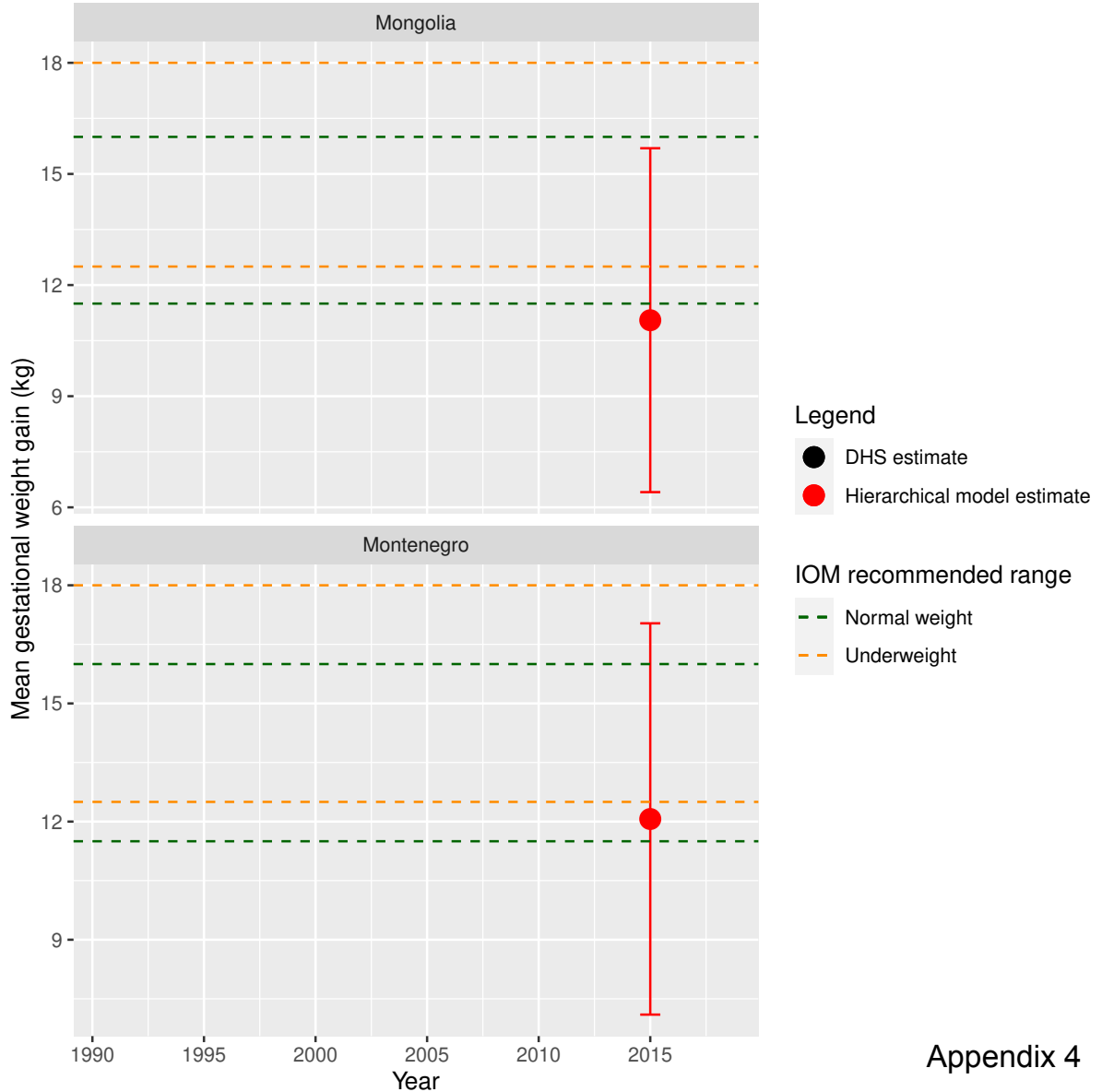

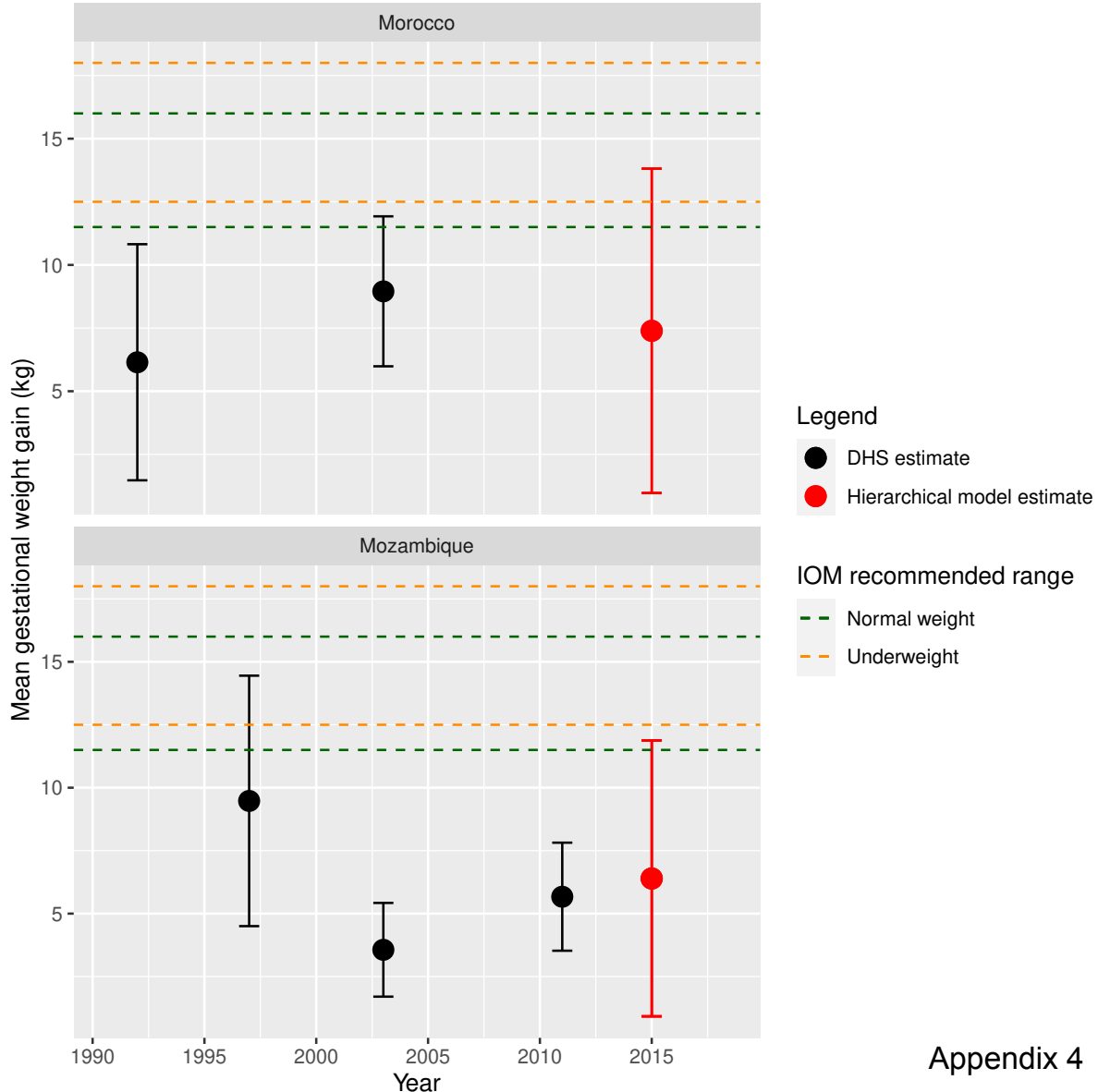

Appendix 4

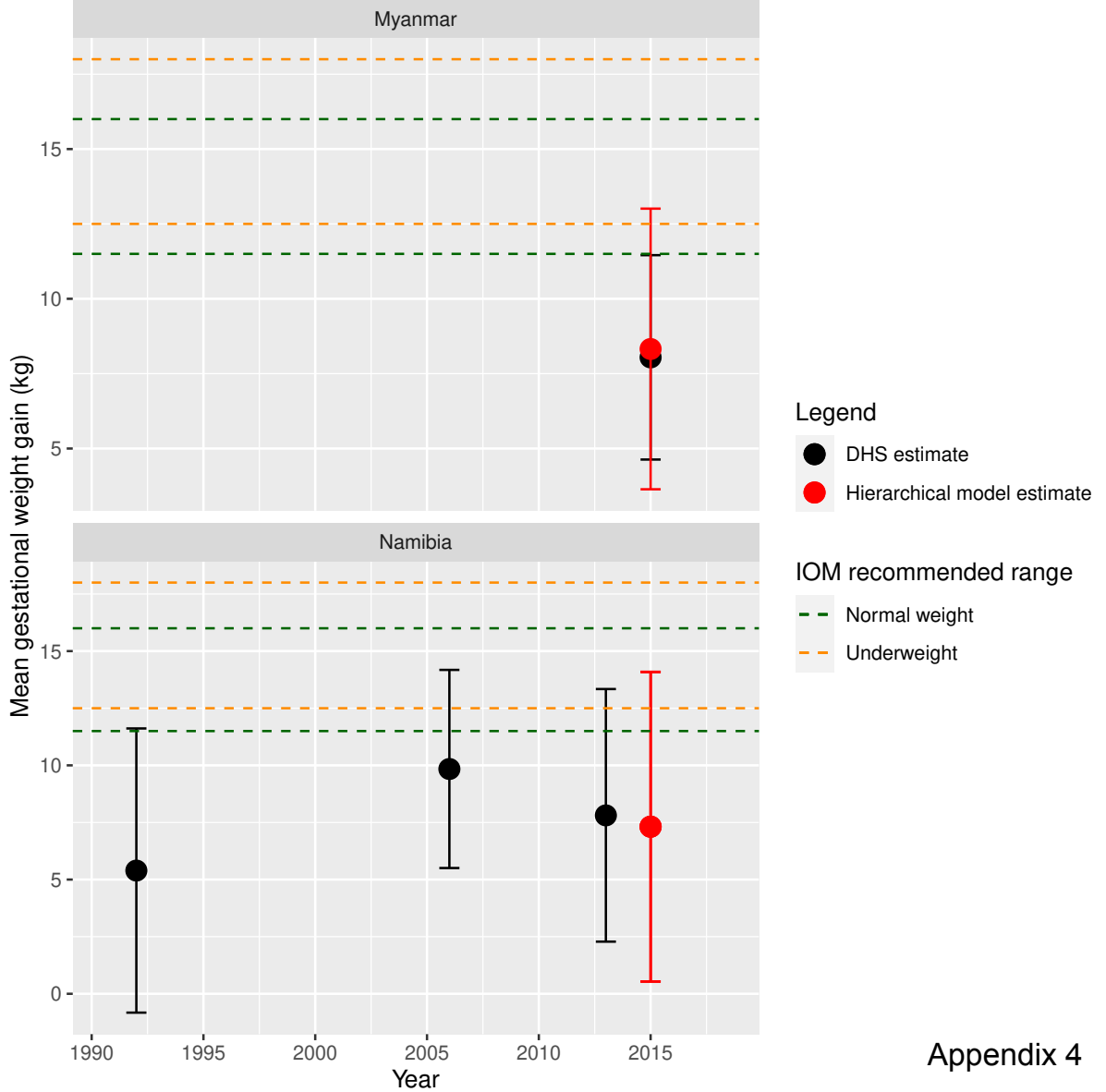

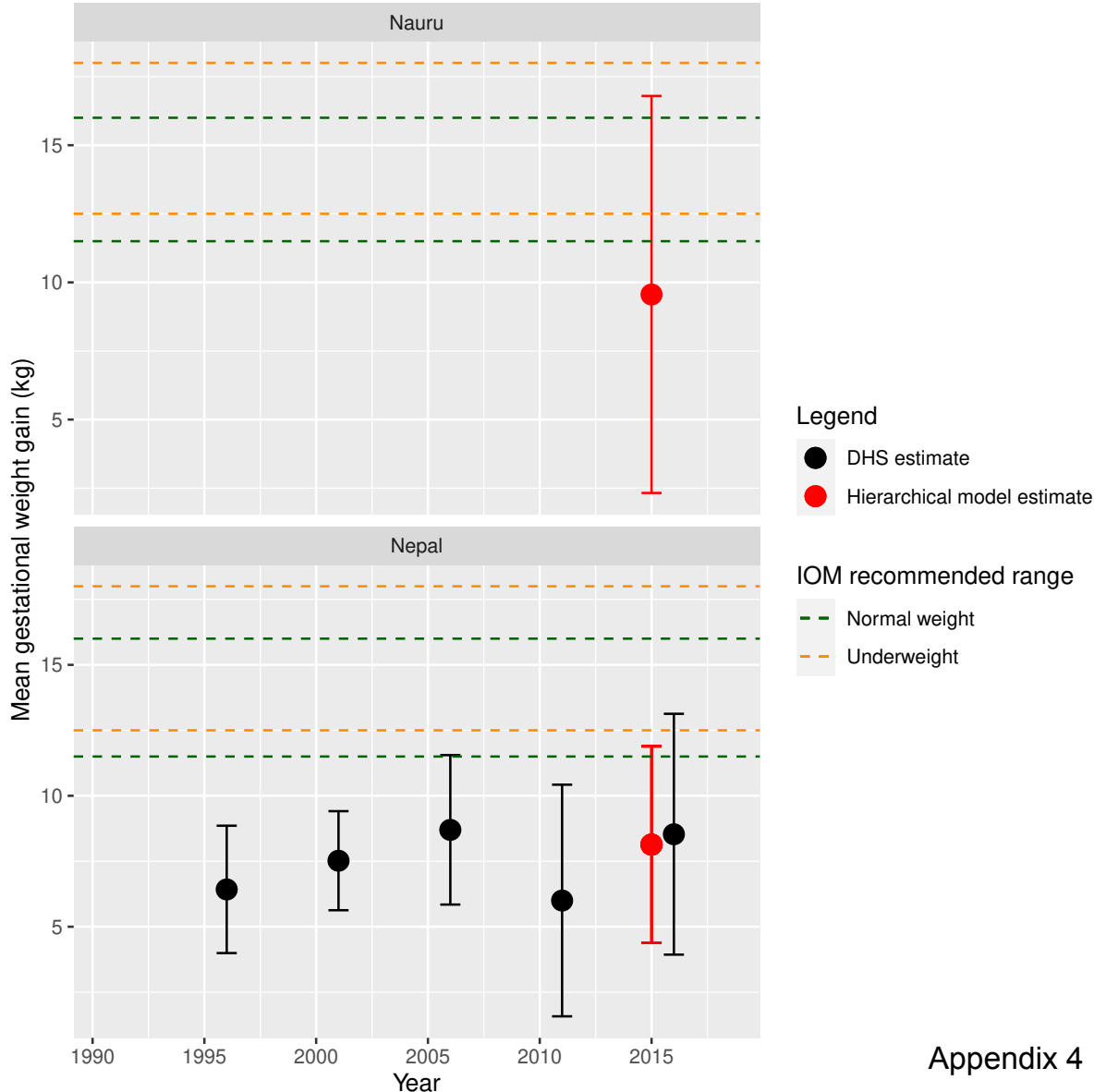

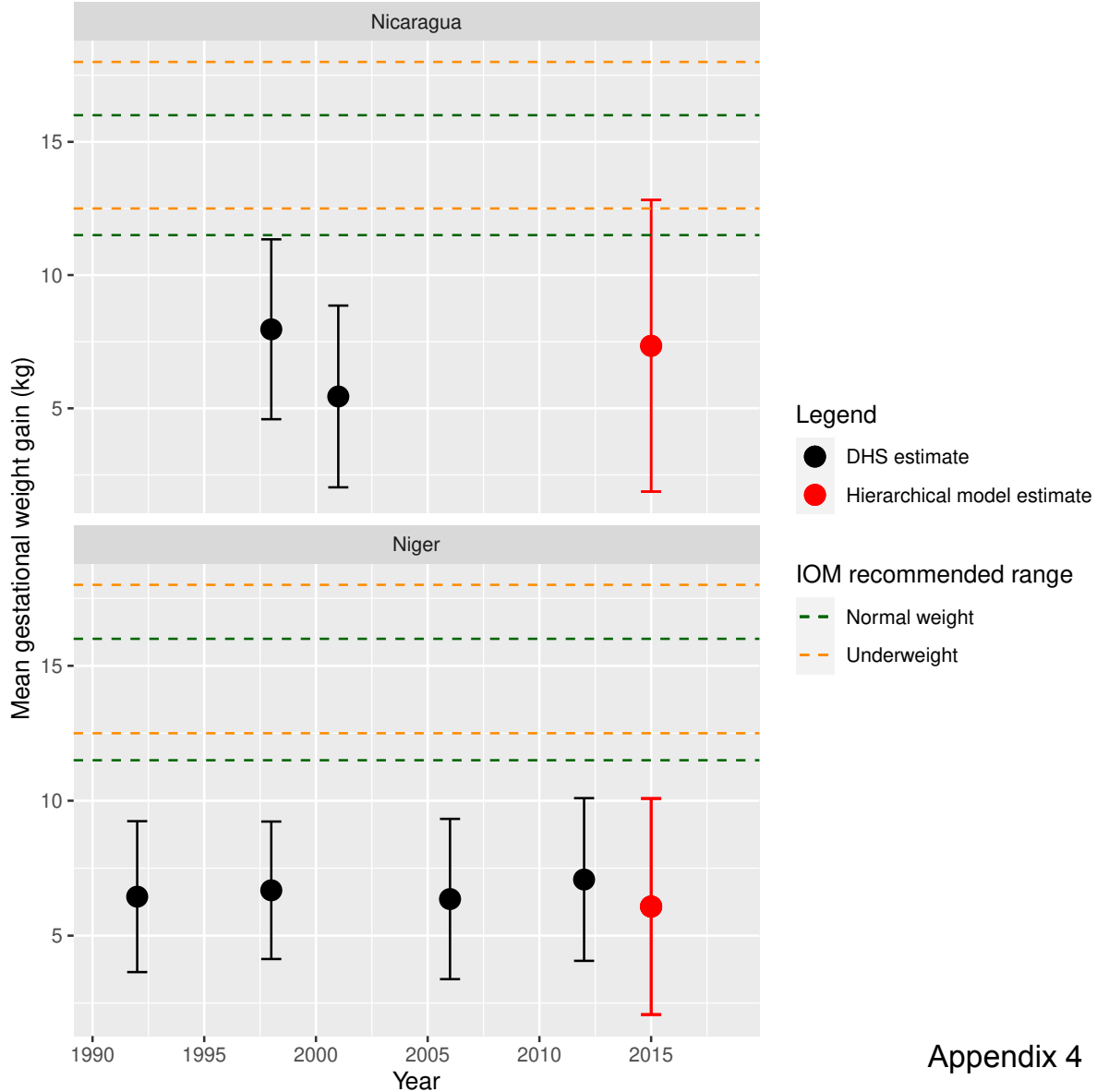

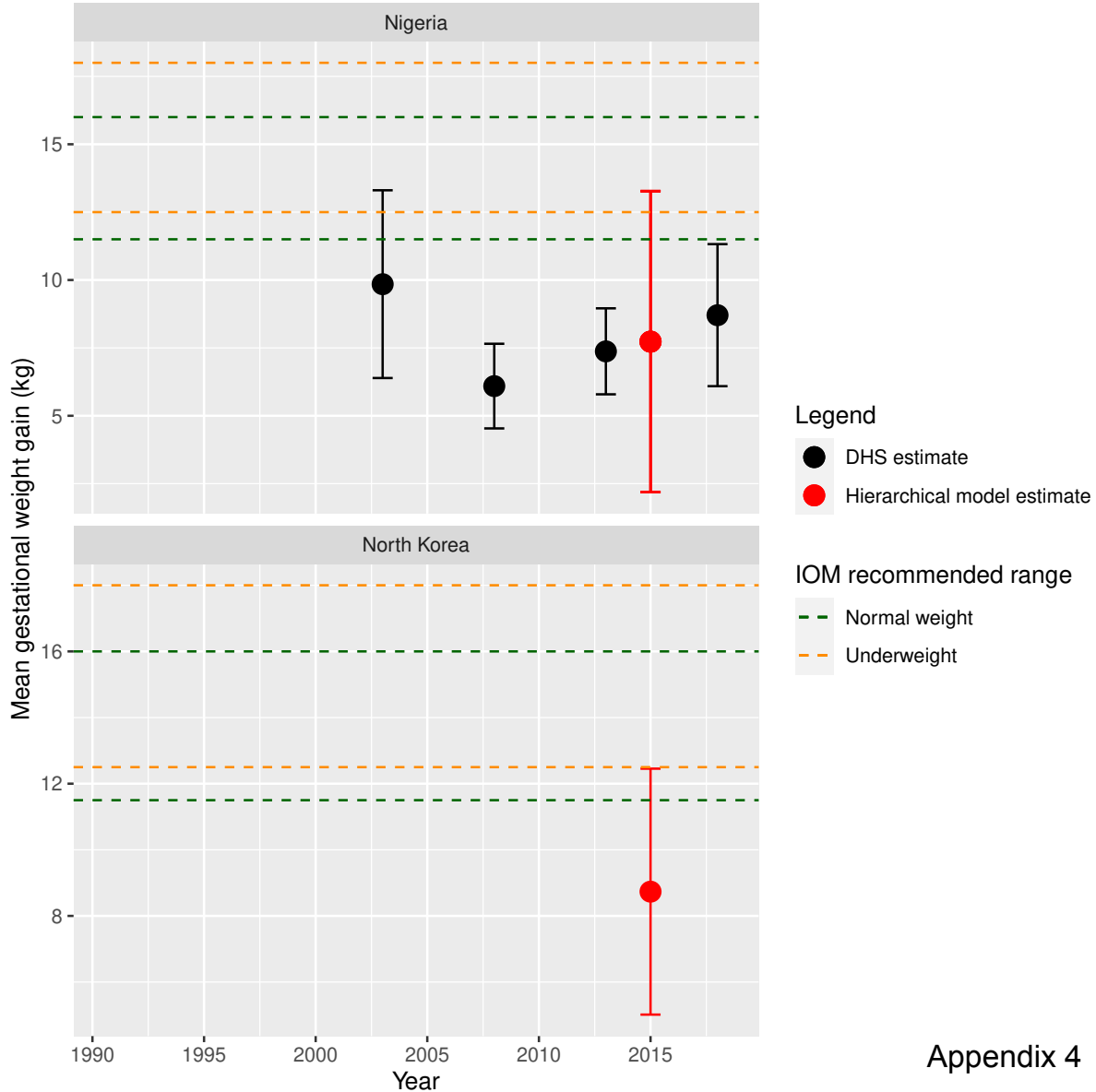

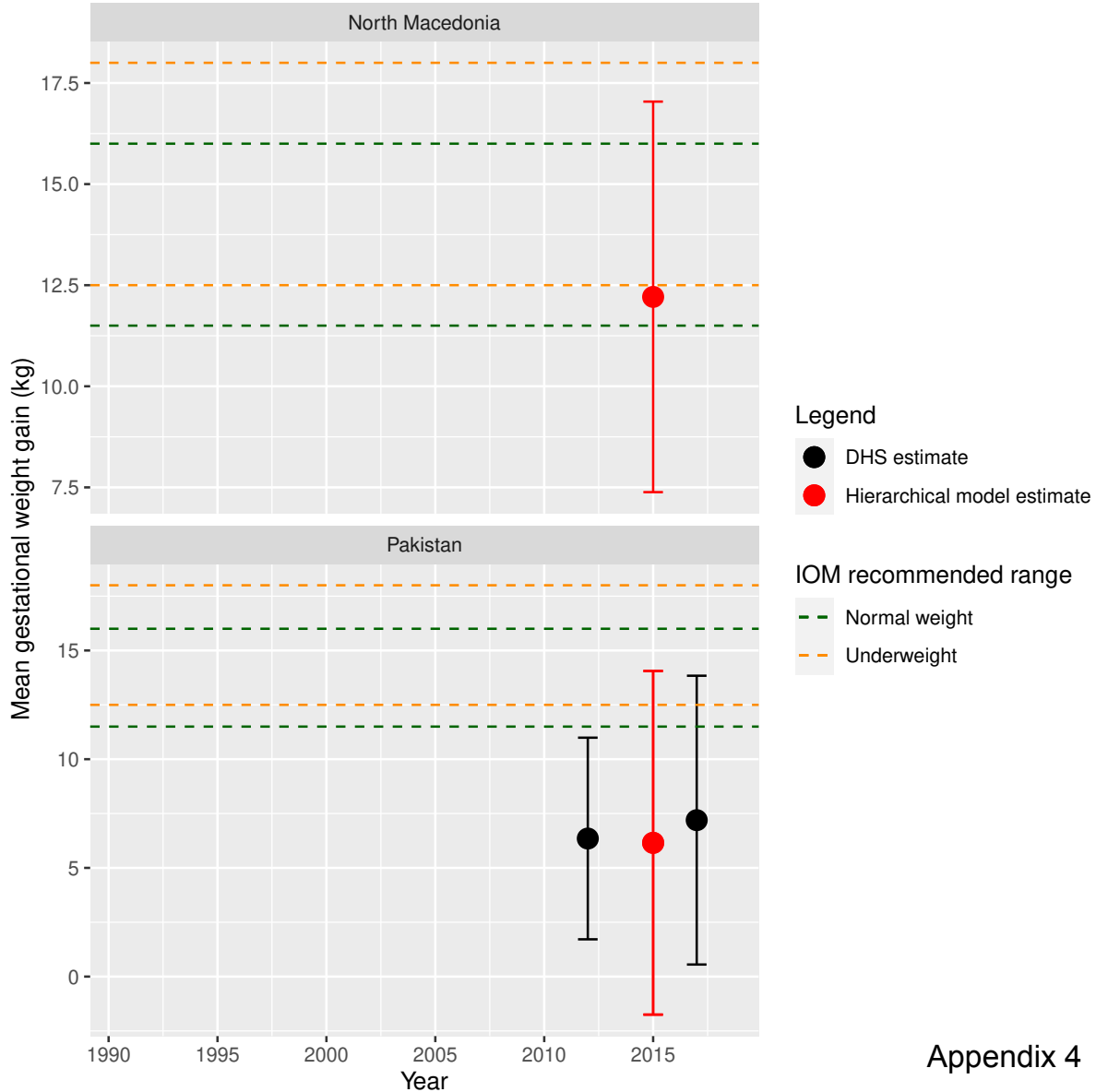

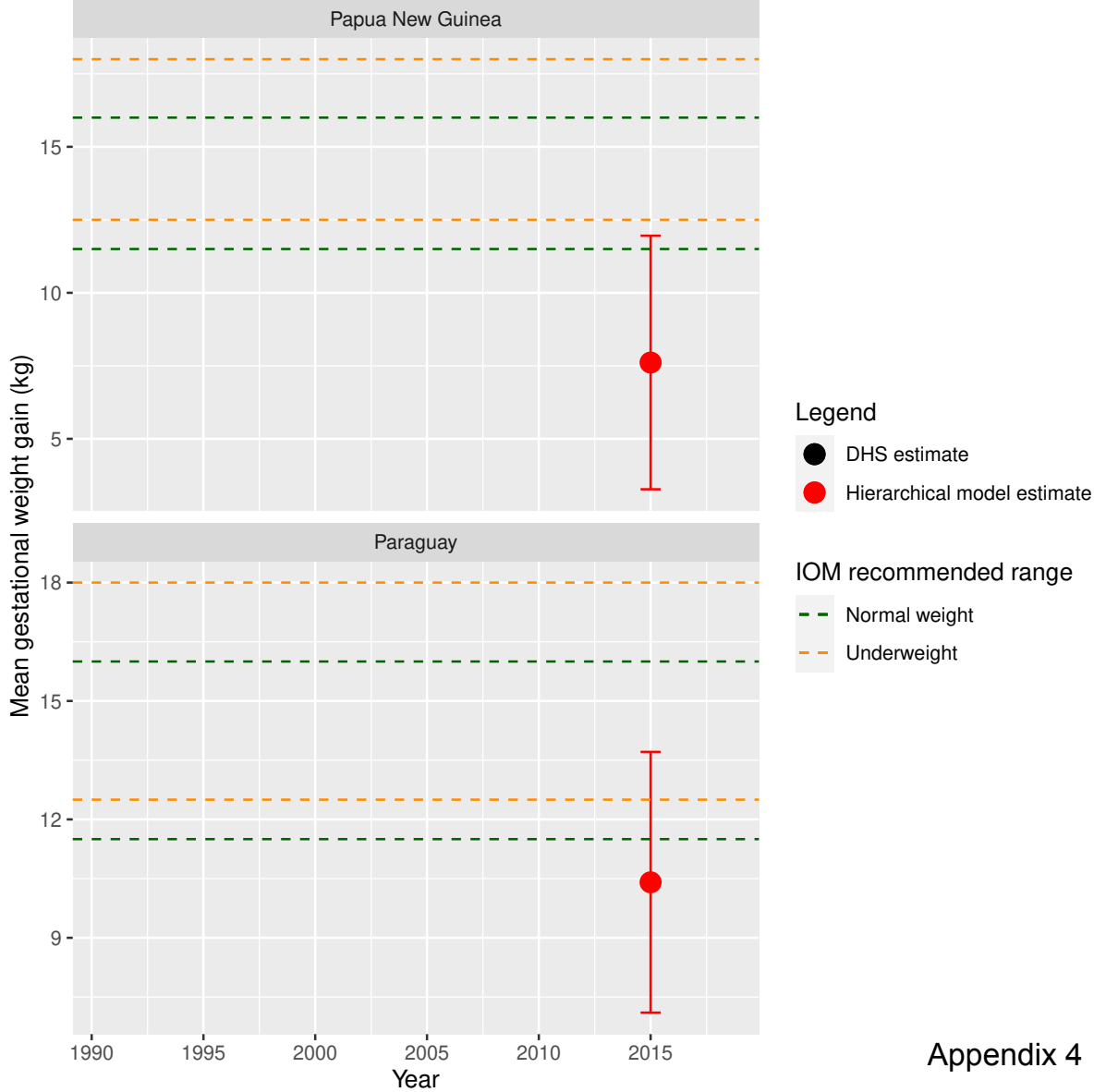

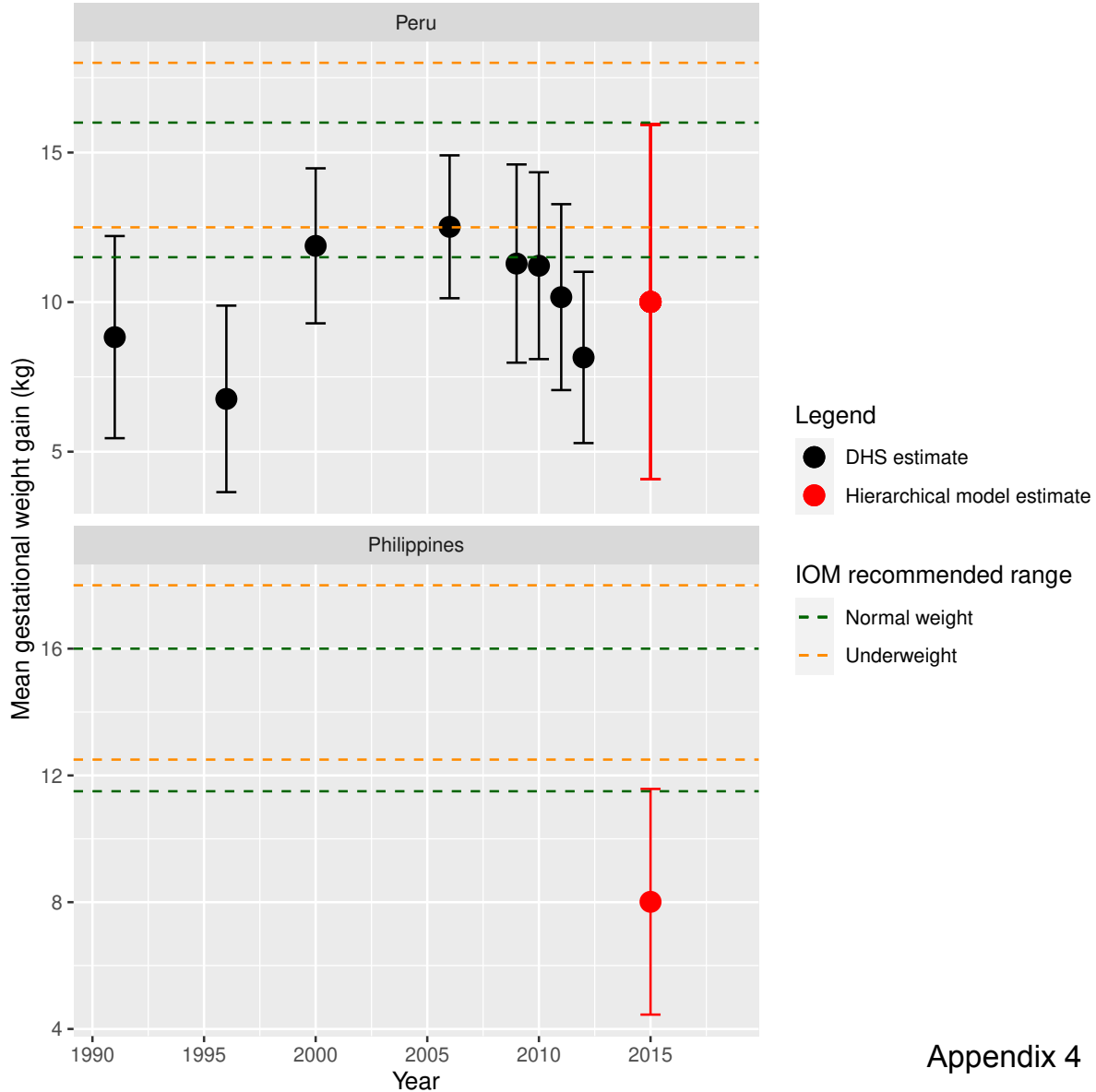

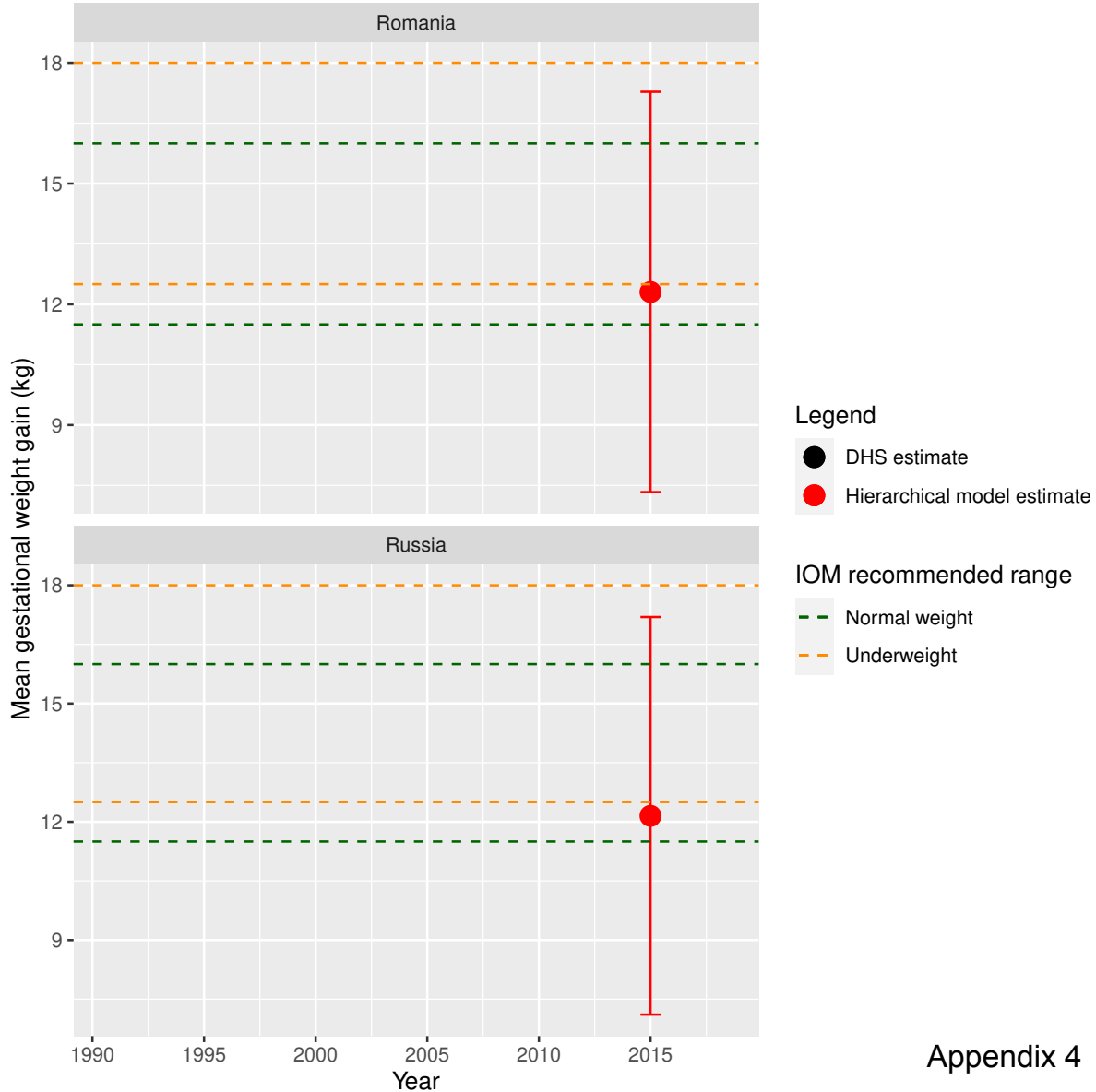

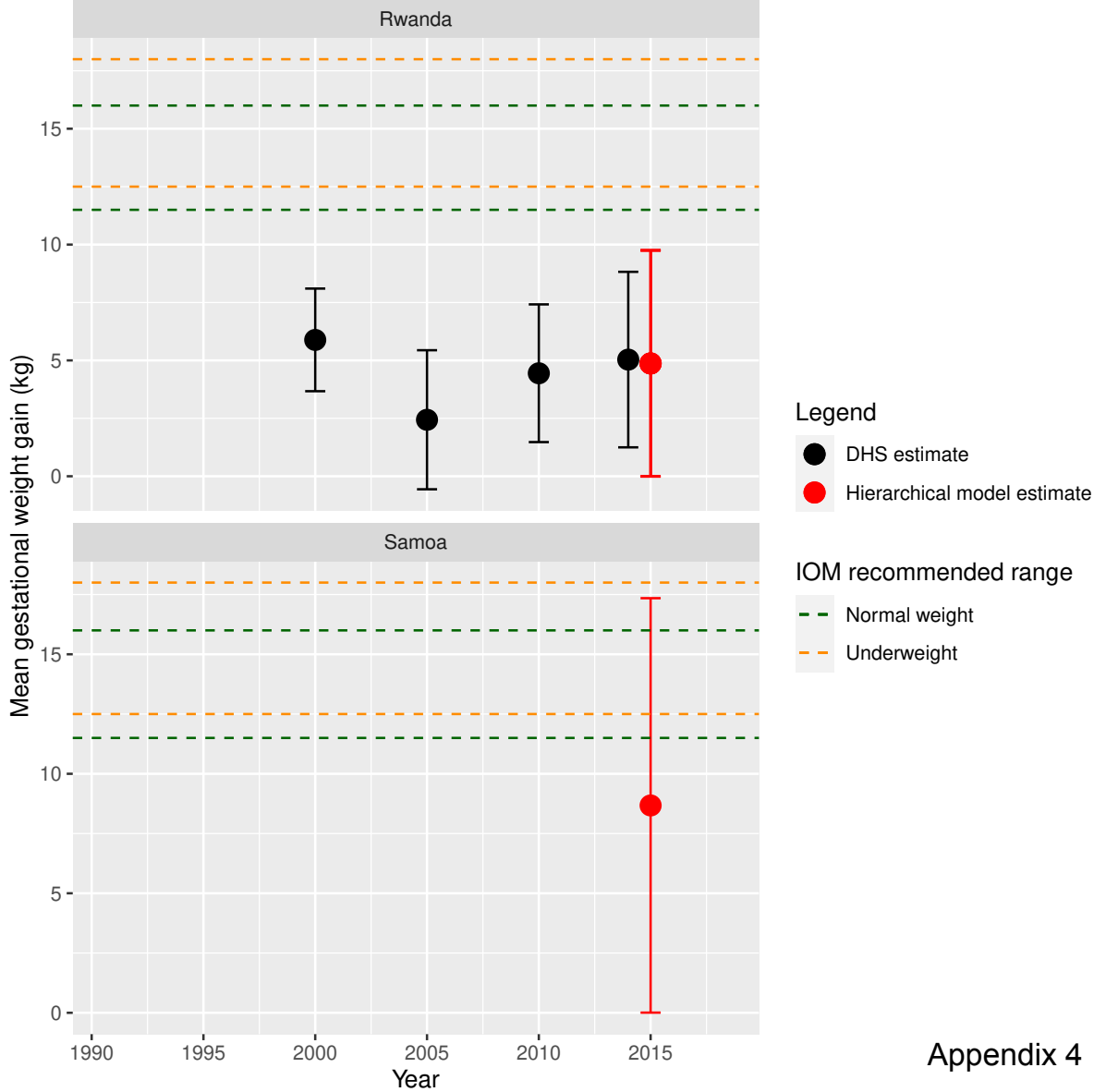

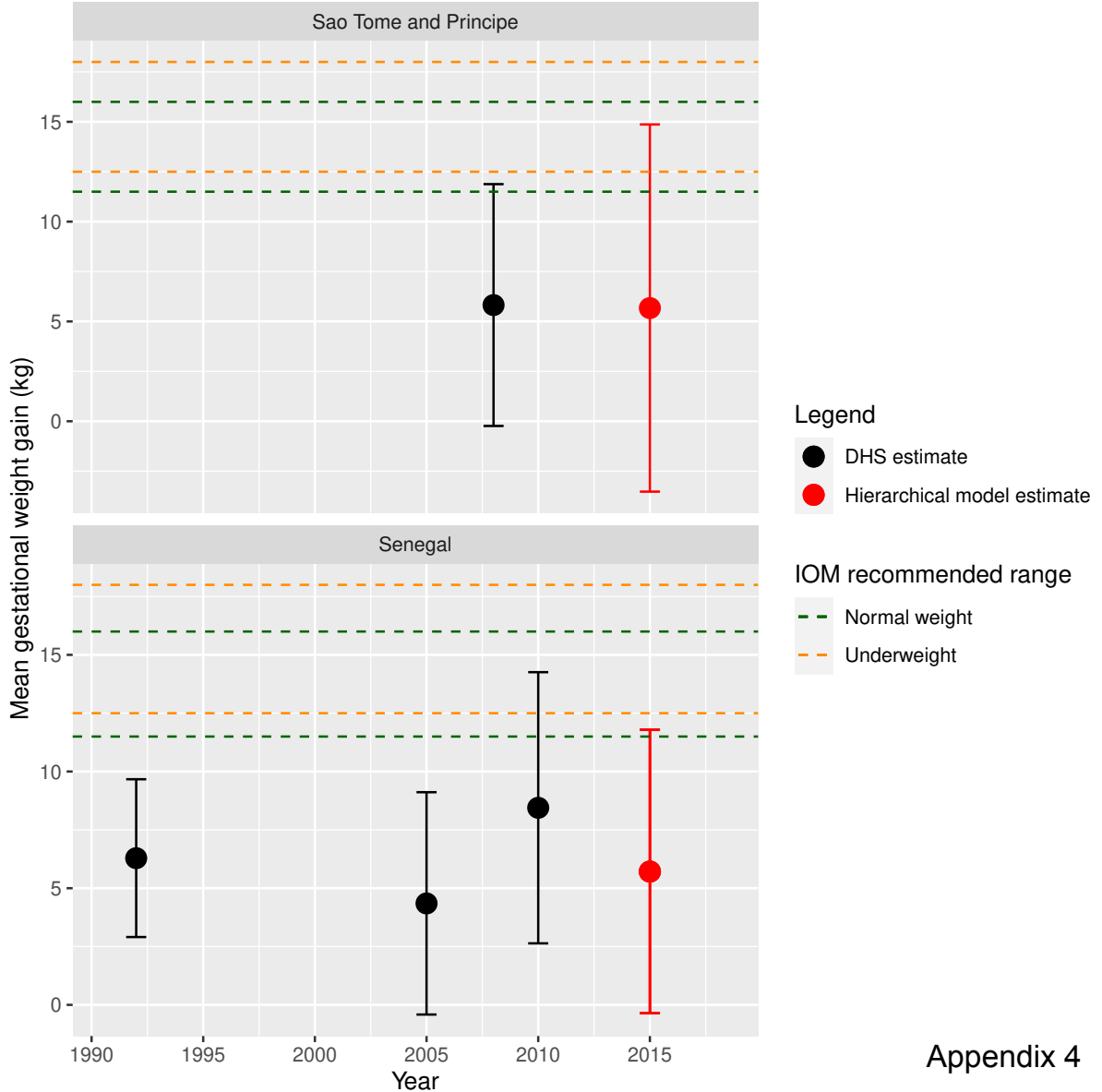

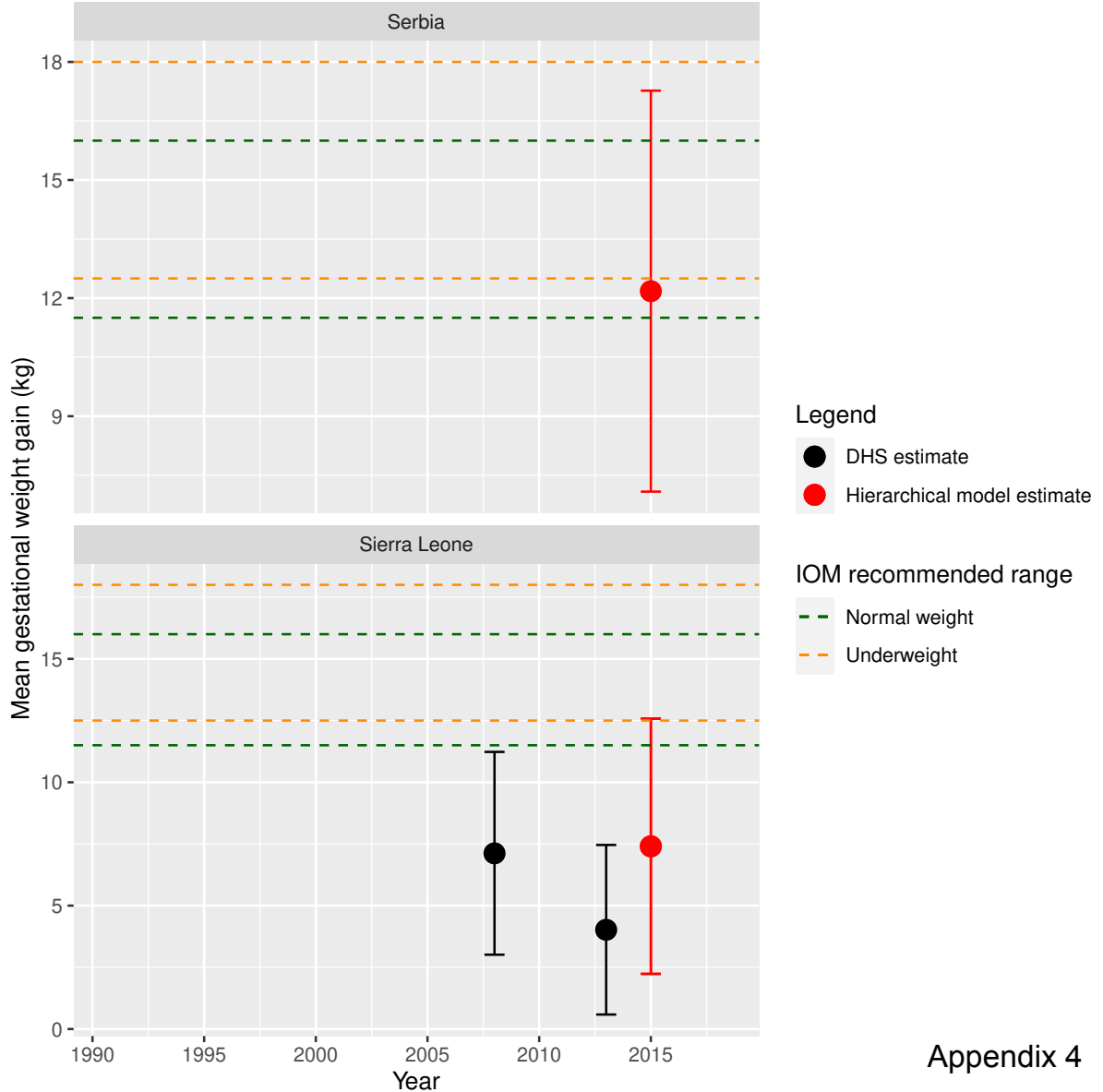

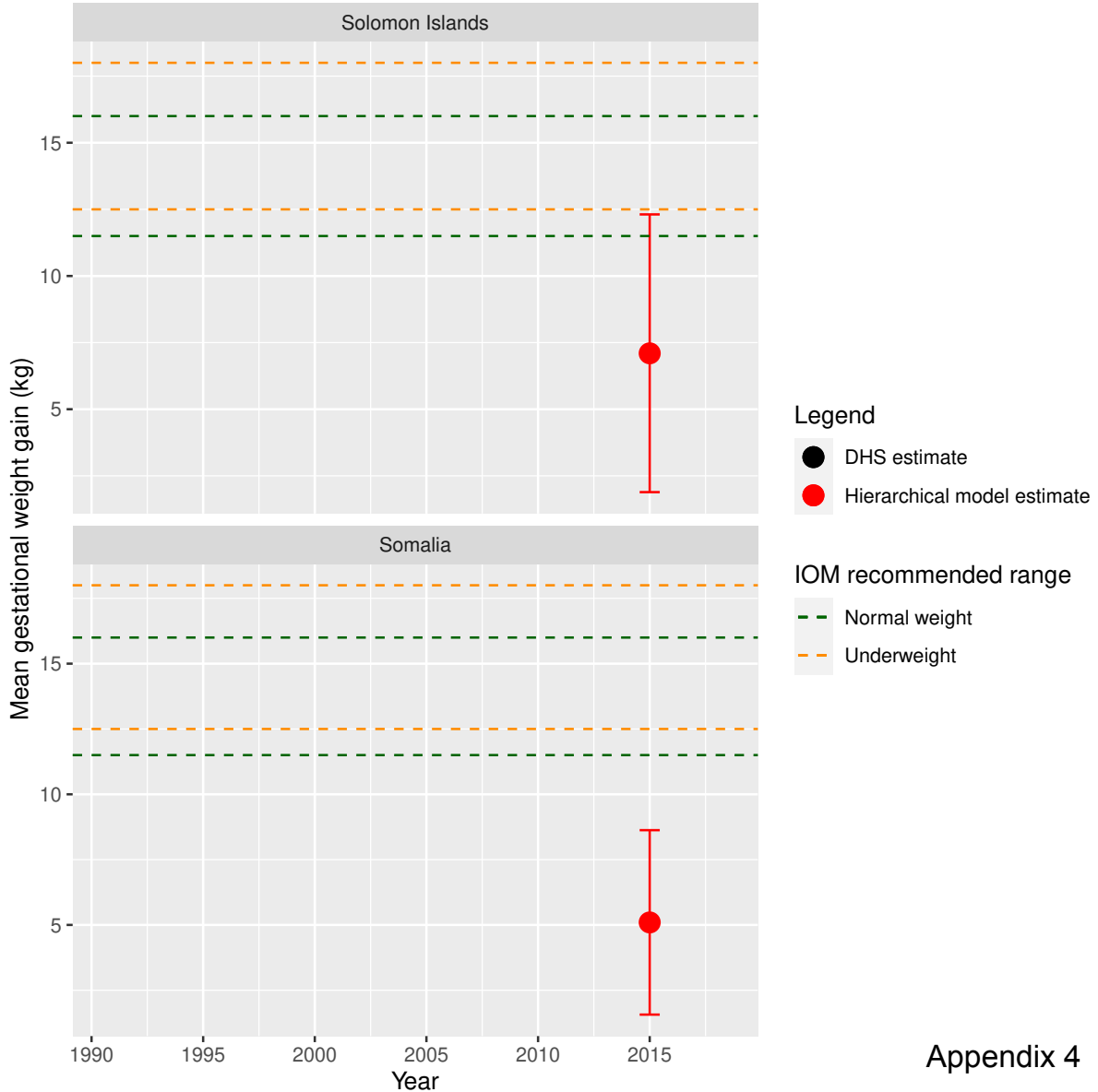

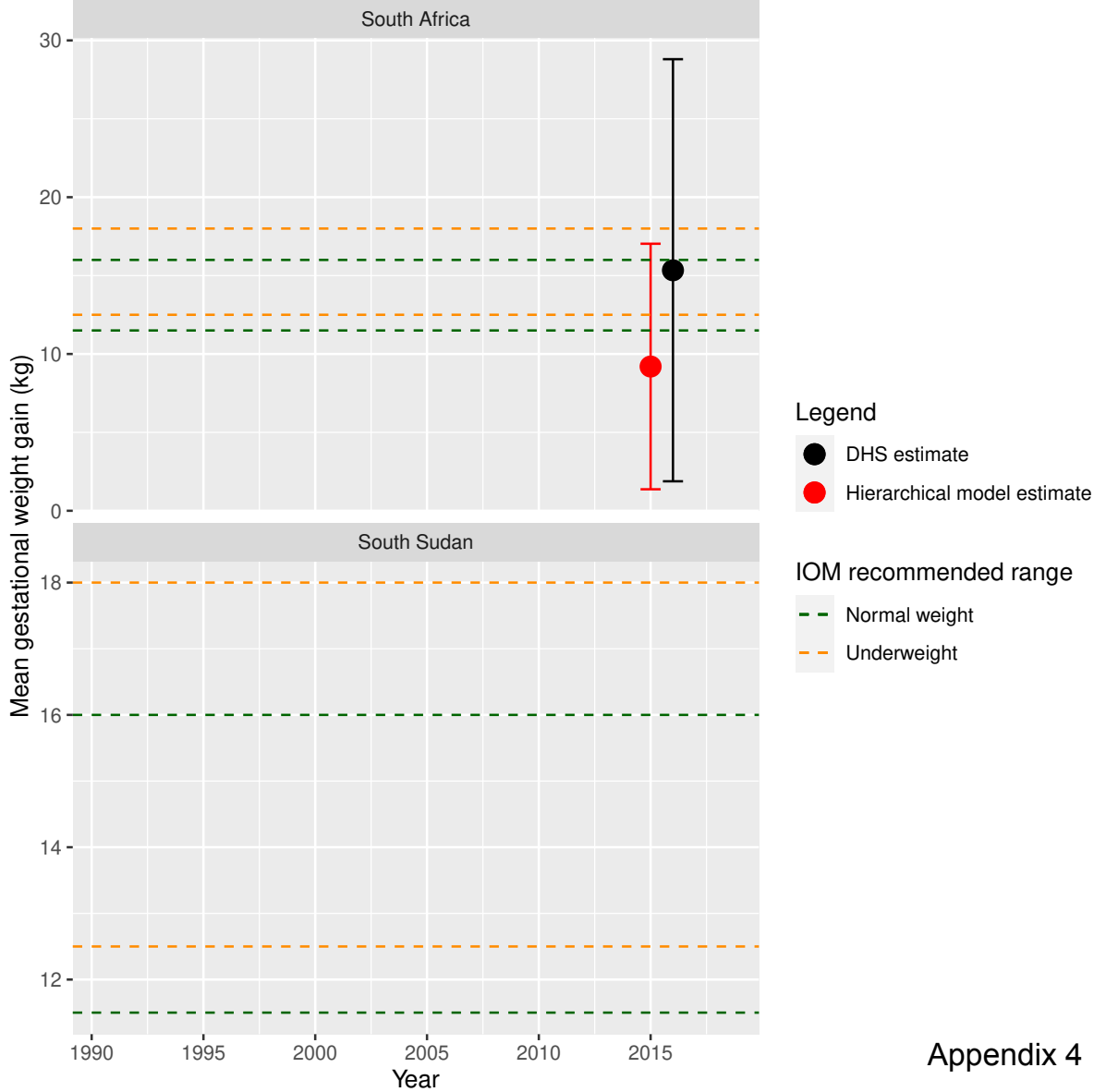

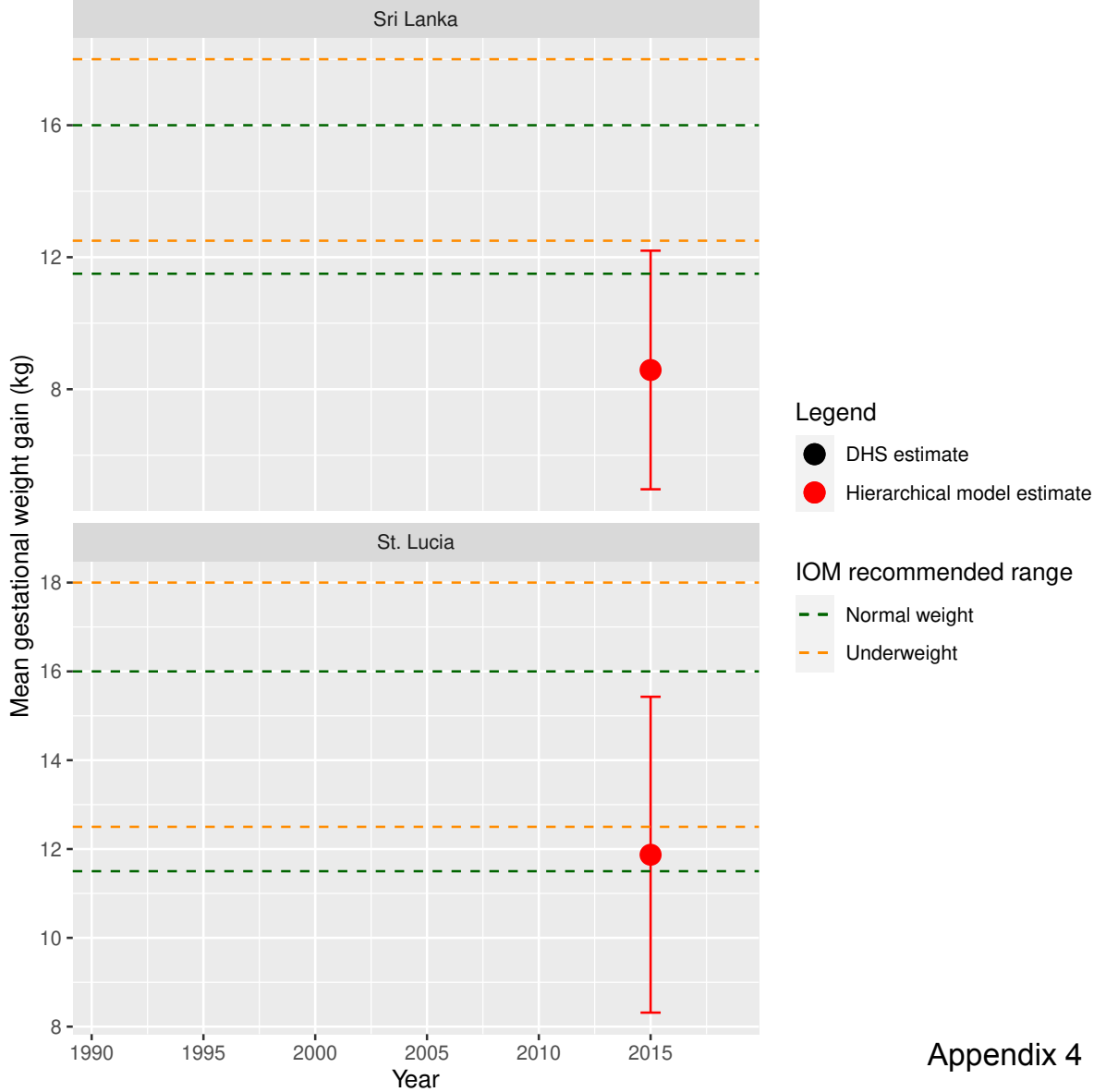

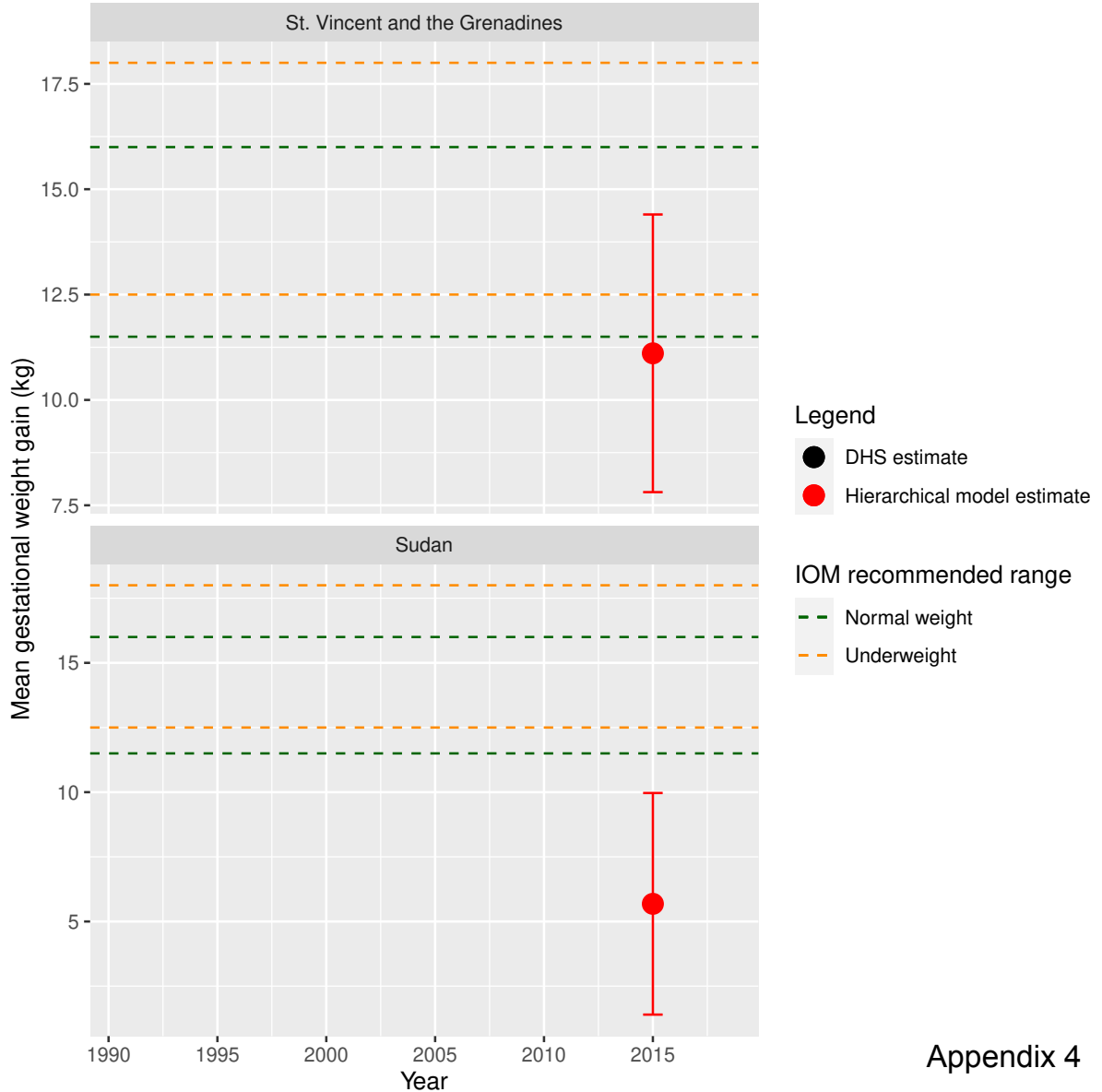

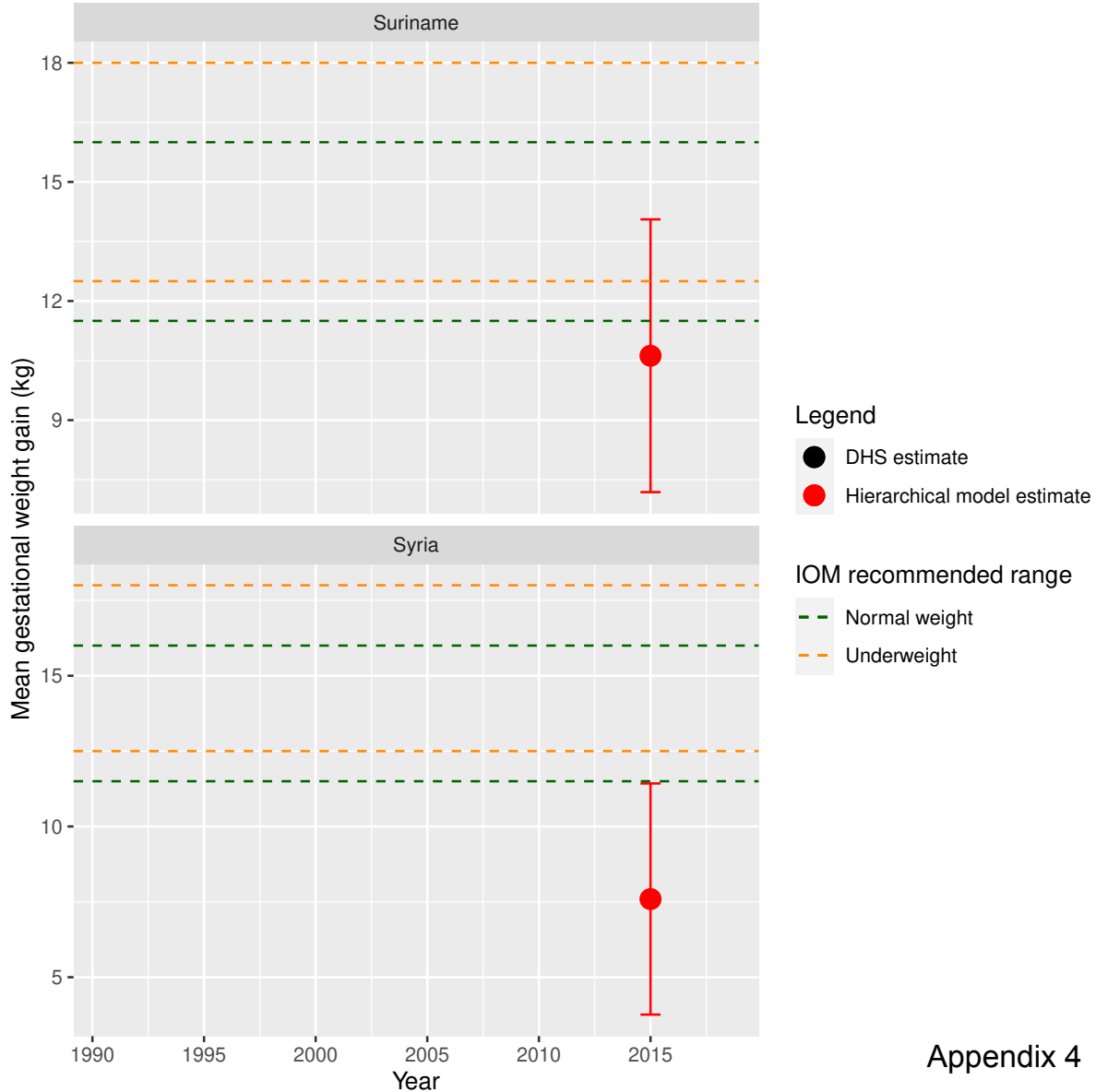

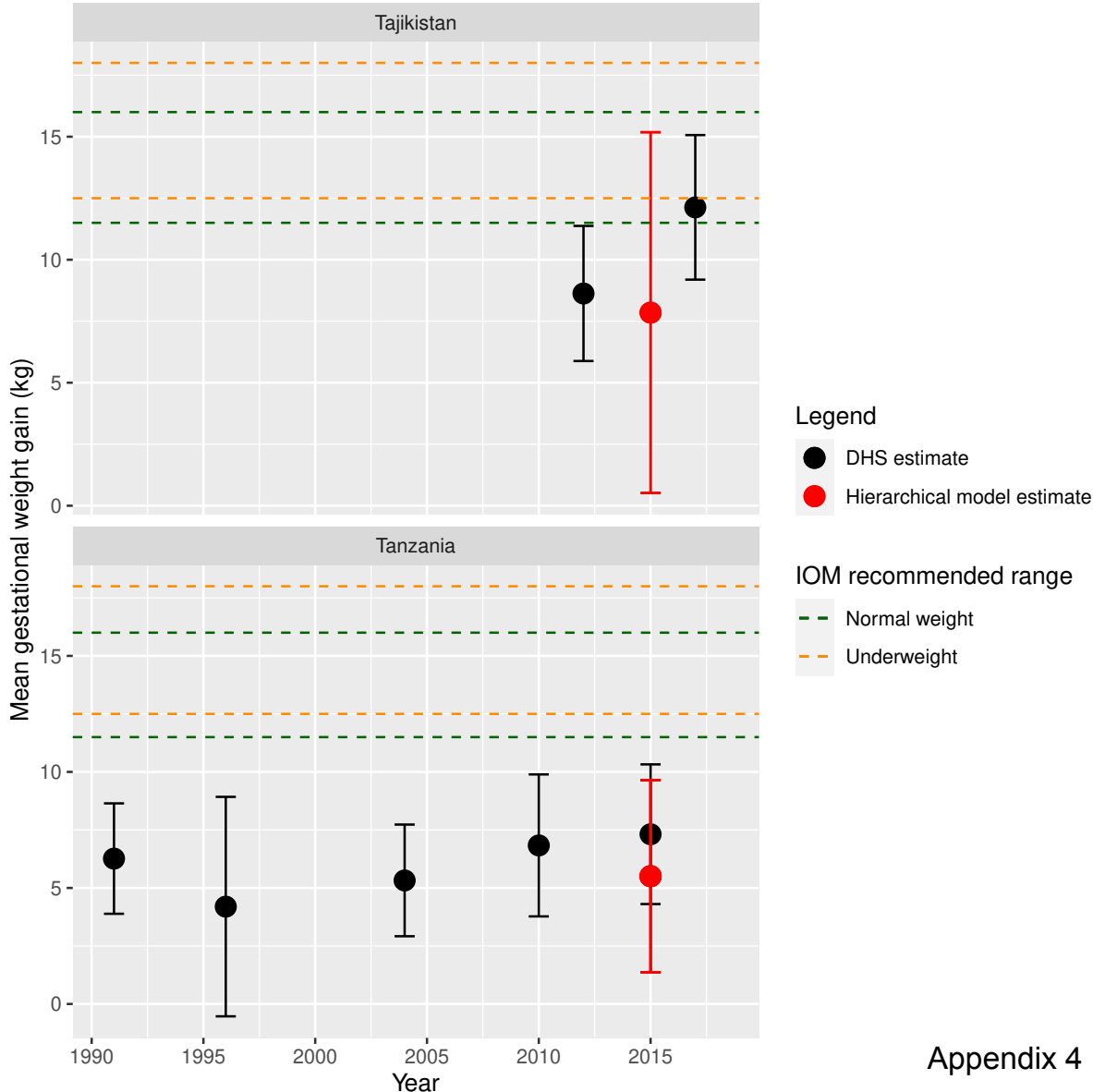

Appendix 4

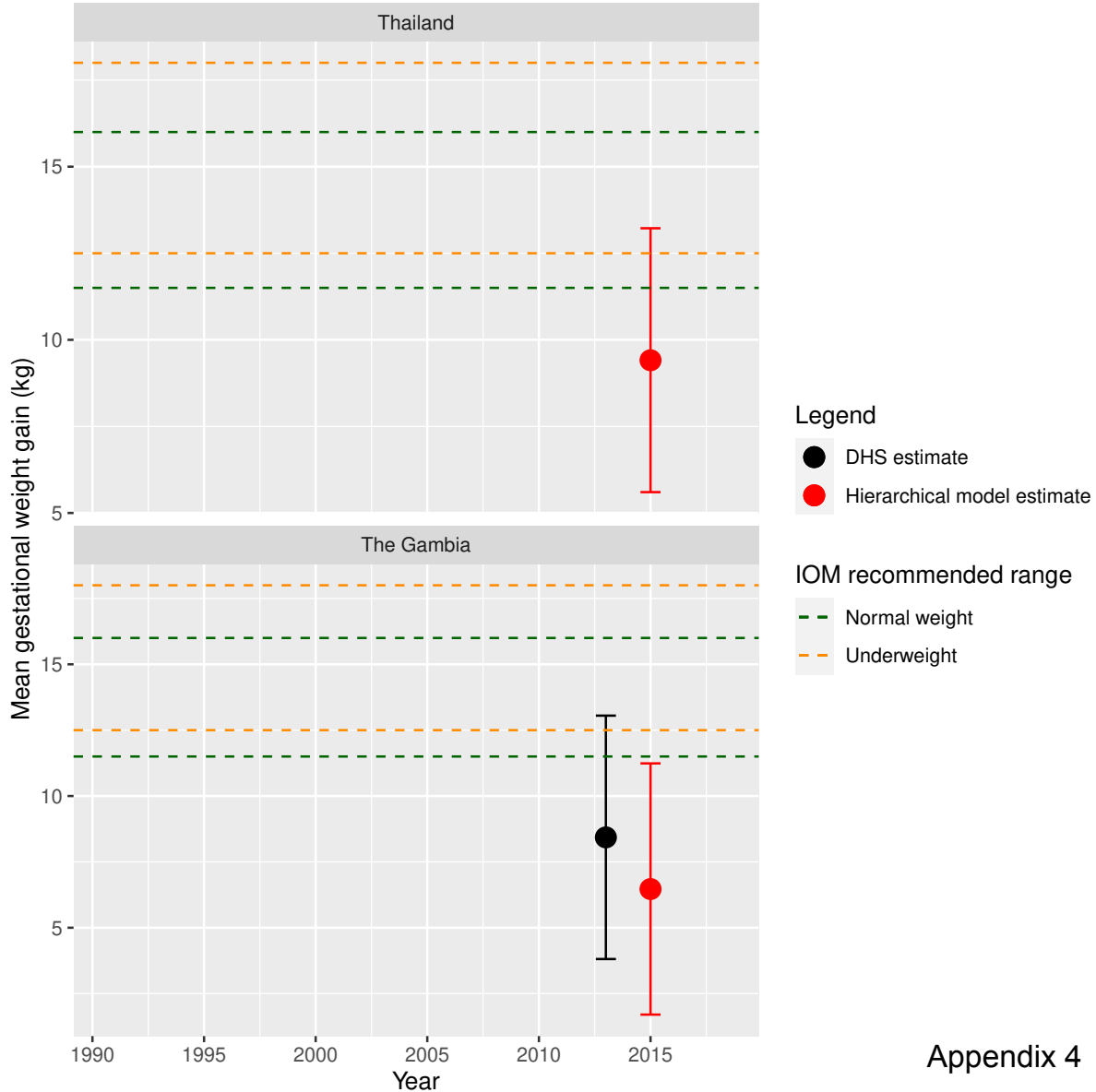

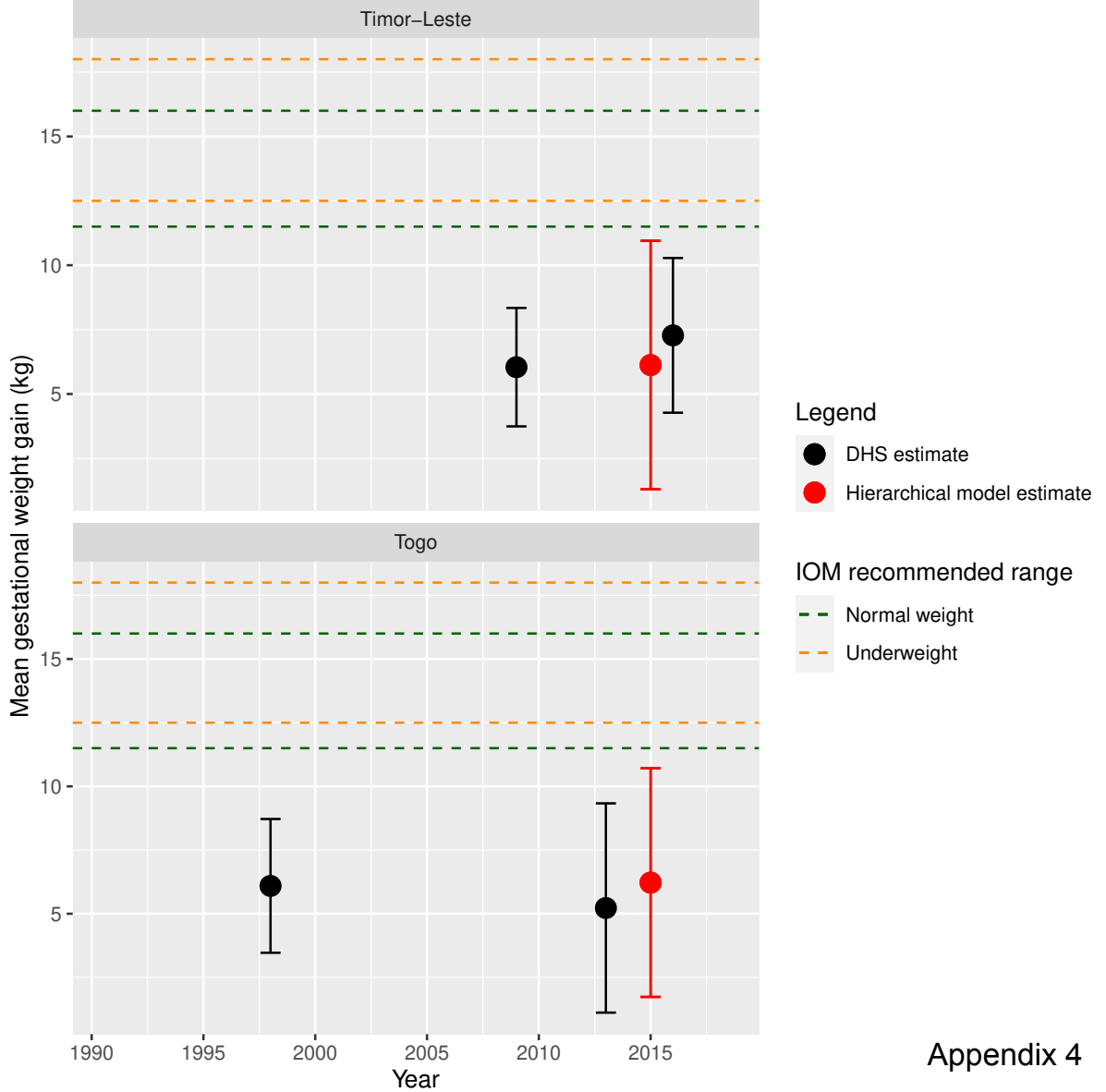

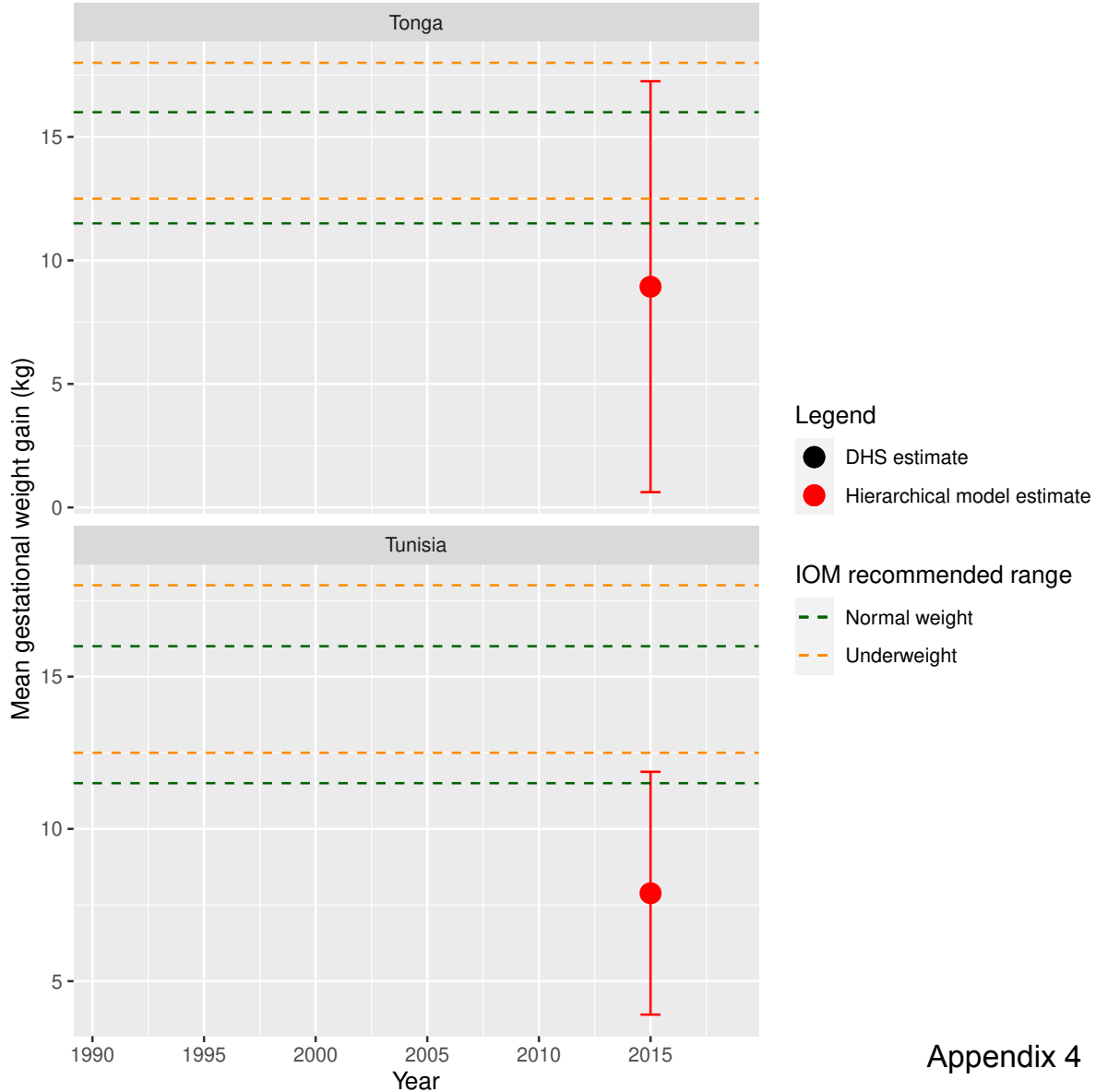

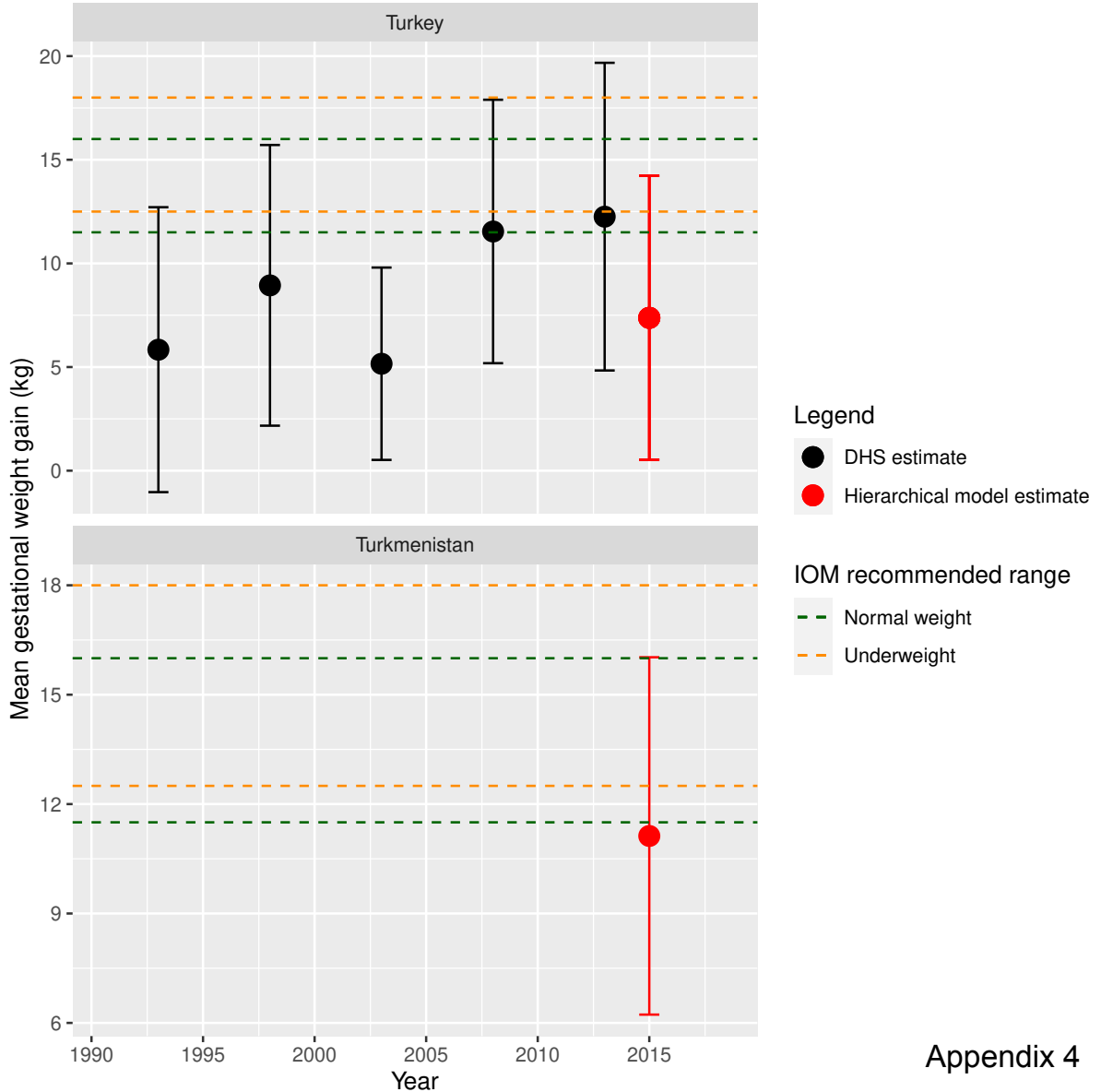

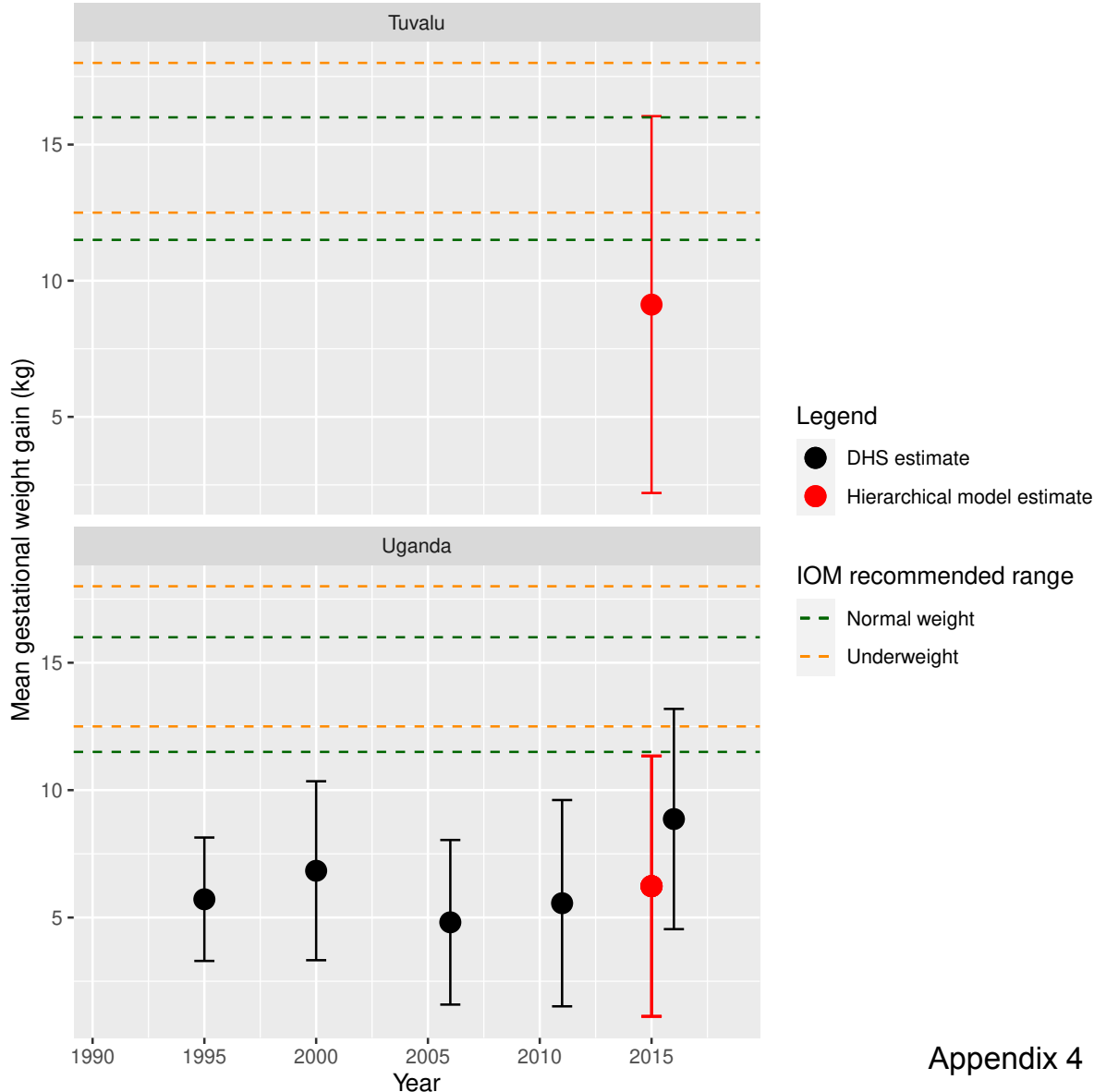

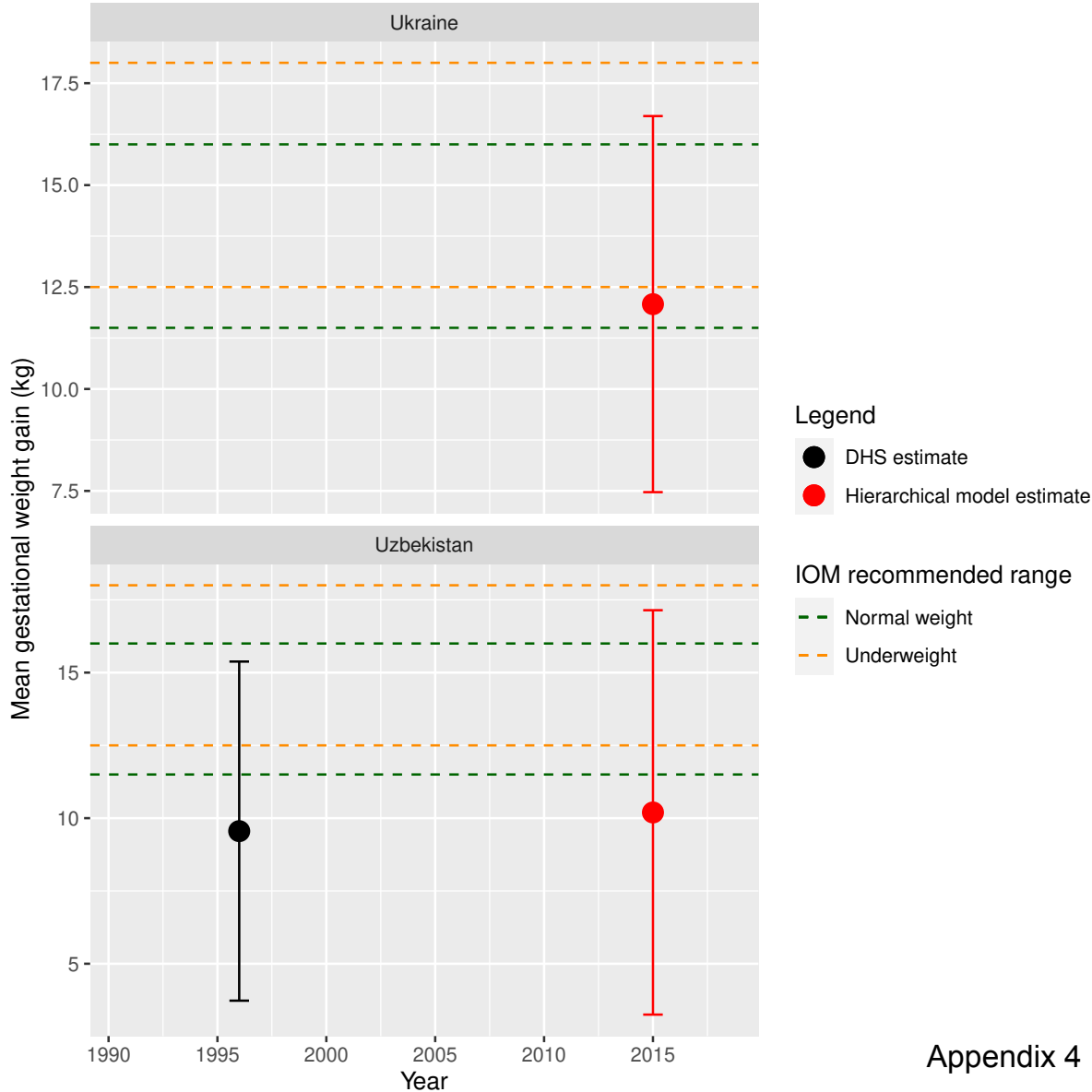

Appendix 4

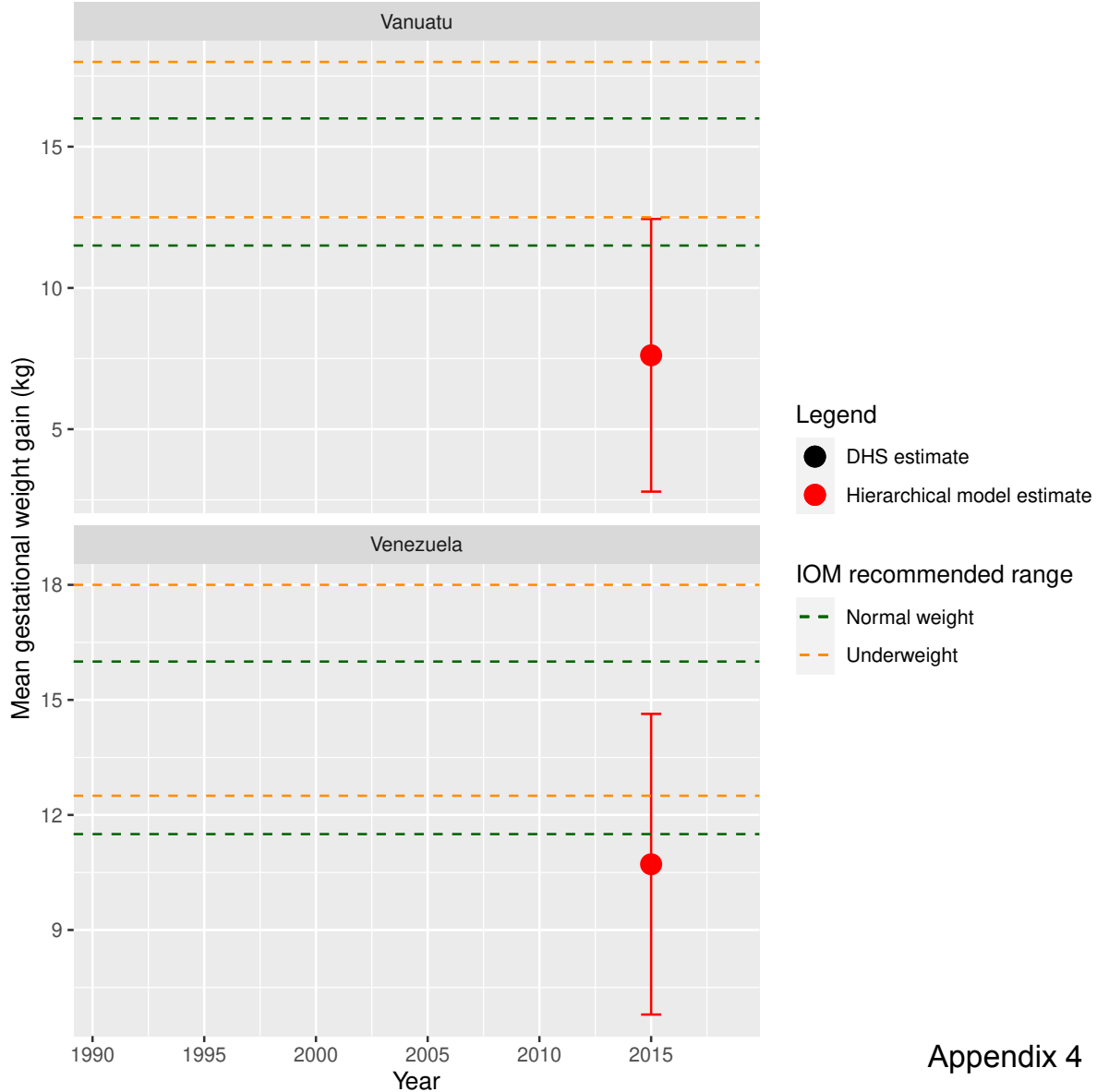

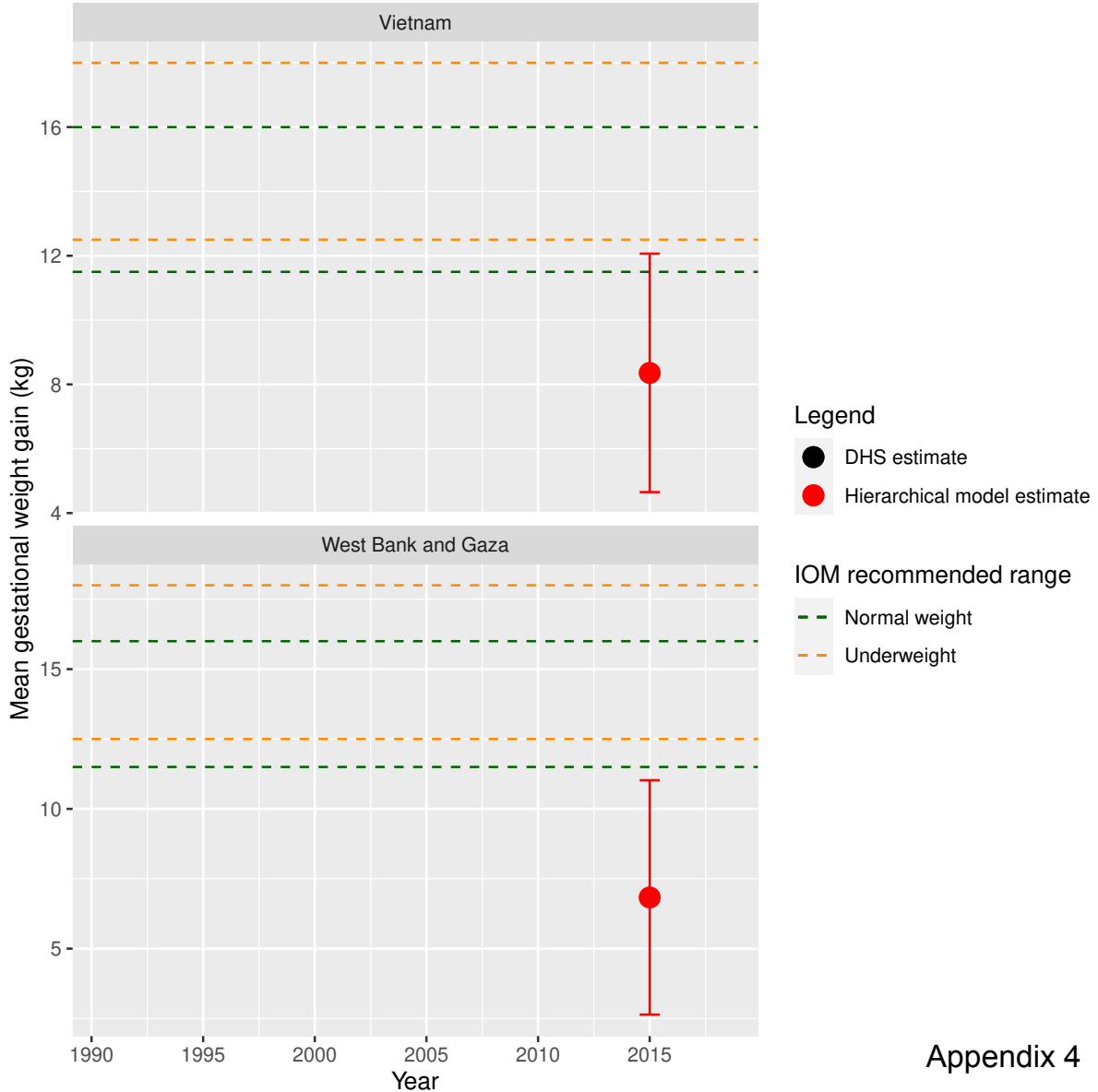

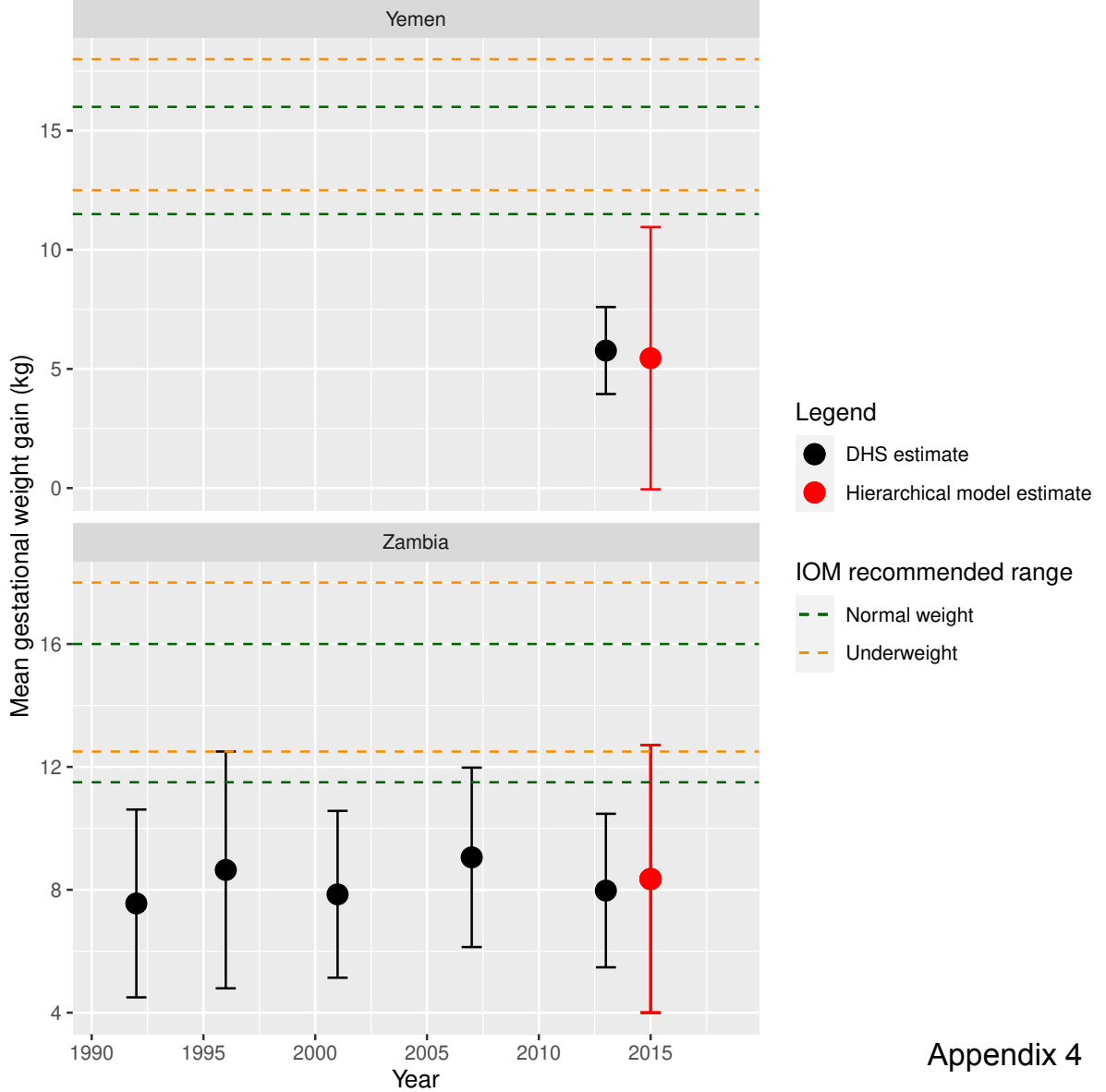

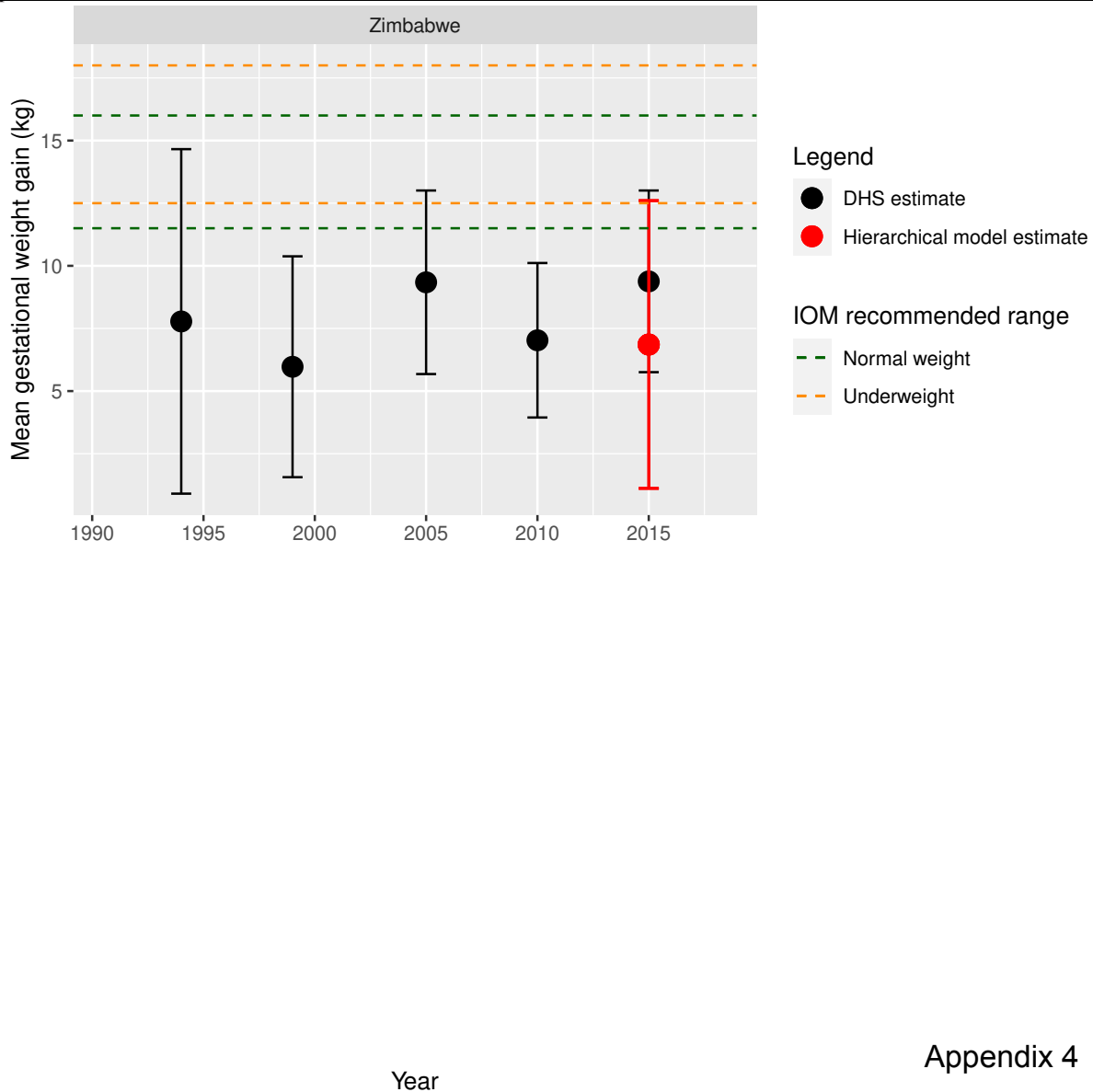

Supplement: Supplementary data [file bmjgh-2020-003423supp004.pdf]
